# The *Hemileia vastatrix* effector HvEC‐016 suppresses bacterial blight symptoms in coffee genotypes with the *S*_*H*_1 rust resistance gene

**DOI:** 10.1111/nph.14334

**Published:** 2016-12-05

**Authors:** Thiago Maia, Jorge L. Badel, Gustavo Marin‐Ramirez, Cynthia de M. Rocha, Michelle B. Fernandes, José C. F. da Silva, Gilson M. de Azevedo‐Junior, Sérgio H. Brommonschenkel

**Affiliations:** ^1^ Departamento de Fitopatologia and National Institute for Plant‐Pest Interactions/Instituto de Biotecnologia Aplicada a Agropecuária‐BIOAGRO Universidade Federal de Viçosa Viçosa MG 36570‐000 Brazil

**Keywords:** avirulence protein, coffee rust, effector delivery, effector prediction, *Hemileia vastatrix*, necrosis suppression, plant immunity, *Pseudomonas syringae* pv. *garcae*

## Abstract

- A number of genes that confer resistance to coffee leaf rust (*S*_*H*_1–*S*_*H*_9) have been identified within the genus *Coffea*, but despite many years of research on this pathosystem, the complementary avirulence genes of *Hemileia vastatrix* have not been reported.
- After identification of *H. vastatrix* effector candidate genes (*HvEC*s) expressed at different stages of its lifecycle, we established an assay to characterize HvEC proteins by delivering them into coffee cells via the type‐three secretion system (T3SS) of *Pseudomonas syringae* pv. *garcae* (*Psgc*).
- Employing a calmodulin‐dependent adenylate cyclase assay, we demonstrate that *Psgc* recognizes a heterologous *P. syringae* T3SS secretion signal which enables us to translocate HvECs into the cytoplasm of coffee cells. Using this *Psgc*‐adapted effector detector vector (EDV) system, we found that HvEC‐016 suppresses the growth of *Psgc* on coffee genotypes with the *S*_*H*_1 resistance gene. Suppression of bacterial blight symptoms in *S*_*H*_1 plants was associated with reduced bacterial multiplication. By contrast, HvEC‐016 enhanced bacterial multiplication in *S*_*H*_1‐lacking plants.
- Our findings suggest that HvEC‐016 may be recognized by the plant immune system in a *S*_*H*_1‐dependent manner. Thus, our experimental approach is an effective tool for the characterization of effector/avirulence proteins of this important pathogen.

## Introduction

Coffee leaf rust, caused by the biotrophic pathogen *Hemileia vastatrix*, is the most important fungal disease affecting coffee production. Although the use of fungicides can control the disease efficiently, the economically most favorable alternative is the use of resistant cultivars because it reduces the cost of production and has no negative impact on the environment. However, the fungus overcomes coffee resistance very rapidly, which makes it difficult for the producer to avoid the use of fungicides (Várzea & Marques, 2005). To date, 49 races of the pathogen have been described (Gichuru *et al*., 2012). Such a large number of races is consistent with the high genetic variability reported for the fungus (Nunes *et al*., 2009; Maia *et al*., 2013). In addition, these various races provide an important framework for understanding the genetics of coffee resistance against rust.

Challenge of coffee plants with different races of *H. vastatrix* resulted in the identification of at least nine genes (*S*_*H*_1–*S*_*H*_9) that confer resistance to leaf rust (Rodrigues‐Junior *et al*., 1975; Bettencourt & Rodrigues‐Junior, 1988), of which only the *S*_*H*_ *3* locus has been mapped and shown to correspond to a complex of three to five genes on a homeologous chromosome from three different *Coffea* genomes (Ribas *et al*., 2011). *S*_*H*_ genes generally confer a post‐haustorial resistance that results in the hypersensitive response (Martins & Moraes, 1996; Silva *et al*., 2002; Ramiro *et al*., 2009). The assumption that the gene‐for‐gene theory (Flor, 1942) applies to the *Coffea*–*H. vastatrix* pathosystem has been used to infer the possible genotypes of different races of the pathogen (Noronha‐Wagner & Bettencourt, 1967). Hence, it can be assumed that the determination of an incompatible interaction between a particular race of the fungus and a resistance host genotype is based on the recognition of the product of avirulence (*Avr*) genes in the pathogen.

It is now widely accepted that pathogens secrete effector proteins to promote acquisition of nutrients and to subvert host defense responses (Dodds *et al*., 2009; Panstruga & Dodds, 2009). Some of these effector proteins are recognized directly or indirectly by plant resistance proteins, eliciting a series of responses referred to as effector triggered immunity (ETI) that in many cases culminate in a programmed cell death known as the hypersensitive response (HR) (Stergiopoulos & de Wit, 2009; Dodds & Rathjen, 2010). Effector proteins recognized by the plant immune system affect the pathogen's virulence phenotype, and therefore, some are referred to as Avr proteins and their coding genes as *Avr* genes. In the absence of their corresponding resistance proteins, effectors can contribute to virulence (Grant *et al*., 2006; Jones & Dangl, 2006).

Effector complements of numerous filamentous plant pathogens have been predicted based on particular characteristics of their amino acid sequences. The presence of a signal for secretion, a small size, and absence of additional targeting sequences or transmembrane domains are common characteristics among predicted effectors (Kamoun, 2006; Kämper *et al*., 2006; Haas *et al*., 2009; Stergiopoulos & de Wit, 2009; Kemen *et al*., 2011; Saunders *et al*., 2012; Sperschneider *et al*., 2015). Amino acid motifs have been identified in particular taxonomic groups of filamentous pathogens, most notoriously the RxLR (Birch *et al*., 2006; Jiang *et al*., 2008), CHxC (Kemen *et al*., 2011) and LFLAK motifs in oomycetes (Haas *et al*., 2009), and the [YFC]xW motif in rust (Hacquard *et al*., 2012; Talhinhas *et al*., 2014) and powdery mildew fungi (Godfrey *et al*., 2010; Spanu *et al*., 2010). Nonetheless, candidate effector proteins from a vast number of fungi have been assumed based on their lack of similarity of the mature protein to any amino acid sequence deposited in the databases (Cantu *et al*., 2011, 2013; Duplessis *et al*., 2011; Fernandez *et al*., 2012; Hacquard *et al*., 2012; Saunders *et al*., 2012; Garnica *et al*., 2013; Zheng *et al*., 2013; Bruce *et al*., 2014; Link *et al*., 2014; Nemri *et al*., 2014; Talhinhas *et al*., 2014). Candidate effectors that are expressed preferentially during infection have been prioritized for functional analysis. Despite the rapid progress in effector prediction, the verification of candidates as *bona fide* effectors using functional analysis is lagging behind.

Recently, at least 500 putative *H. vastatrix* secreted proteins were predicted using bioinformatics pipelines (Fernandez *et al*., 2012; Cristancho *et al*., 2014; Talhinhas *et al*., 2014). In this predicted secretome, the authors identified proteins showing similarity to haustorially expressed secreted proteins (HESPs) from the flax rust fungus *Melampsora lini* (Catanzariti *et al*., 2006), and to the bean rust transferred protein 1 (RTP1), found previously in other rust fungi (Kemen *et al*., 2005; Vieira *et al*., 2012; Pretsch *et al*., 2013). Eighty‐two putative secreted proteins were found to be highly enriched in cysteine residues, with nearly 60% of them containing the [YFC]xW motif (Talhinhas *et al*., 2014). However, a large number of these reported sequences are truncated and no functional analysis of candidate *H. vastatrix* effectors has been performed.

The lack of efficient methods for genetic manipulation of both organisms and the obligate biotrophy of the pathogen have limited the use of traditional methods to validate effector and *S*_*H*_ genes in the *Coffea–H. vastatrix* pathosystem. An interesting alternative to overcome this limitation is to use candidate effectors as molecular probes to detect specific phenotypic alterations when expressed in particular host genotypes, which may be indicative of an effector function (Vleeshouwers *et al*., 2008). For instance, effector proteins with avirulence activity can be identified in these assays because their recognition by host resistance proteins elicits a rapid tissue collapse. Even though *Agrobacterium*‐mediated transient expression is an alternative method, it is still difficult to routinely apply it to the *Coffea–H. vastatrix* pathosystem due to the low susceptibility of coffee mesophyll cells to infection by *Agrobacterium* (van Boxtel *et al*., 1995). A method used extensively to express effectors from obligate biotrophic pathogens in the host cytoplasm is the effector detector vector (EDV) system (Sohn *et al*., 2007). This strategy was shown to be successful for cloning the *Hyaloperonospora arabidopsidis* avirulence gene *ATR39* (Goritschnig *et al*., 2012) and for identifying effectors that suppress plant defense responses (Cabral *et al*., 2011; Fabro *et al*., 2011; Badel *et al*., 2013; Liu *et al*., 2016).

Delivery of effector candidates using the EDV system requires a nonpathogenic (e.g. *Pseudomonas fluorescens* EtHAn) or an adapted pathogenic bacterium expressing a type‐three secretion system (T3SS) for protein translocation into the host cytoplasm. An interesting possibility to implement an EDV system in the *Coffea*–*H. vastatrix* pathosystem is to use *Pseudomonas syringae* pv. *garcae* (*Psgc*), the causal agent of bacterial leaf blight of coffee (Amaral *et al*., 1956). This bacterial disease has been reported in several countries in America, Asia and Africa (Amaral *et al*., 1956; Ramos & Shavdia, 1976; Oliveira *et al*., 1991; Korobko & Wondimagegne, 1997; Belan *et al*., 2013). Symptoms caused by the bacterium are characterized by irregular dark‐brown patches on leaves that may or may not be surrounded by a yellow ring (Zambolim *et al*., 2005; Pozza *et al*., 2010). Application of a *Psgc*‐adapted EDV system to functionally characterize effectors will represent an important advancement in our ability to better understand the molecular mechanisms underlying the *Coffea*–*H. vastatrix* interaction. In this study we identify additional candidate *H. vastatrix* effectors based on bioinformatics prediction from transcriptomic data obtained from different prebiotrophic and biotrophic phases of the *H. vastatrix* infection cycle. We confirm eukaryotic secretion, provide information about the intron/exon structure for a select group of these candidates and identify an *H. vastatrix* effector that suppresses the necrosis caused by *Psgc* 1202 in coffee plants carrying the *S*_*H*_1 resistance gene.

## Materials and Methods

Single pustule *H. vastatrix* isolate Hv‐01, previously characterized as race II in our laboratory, was reproduced on seedlings of cultivar Catuaí Vermelho IAC 44 (*Coffea arabica*) and used for expressed sequence tags (ESTs) generation from different stages of the fungus lifecycle (Supporting Information Methods S1–S4). A bioinformatics pipeline was designed in order to predict secreted proteins of unknown function, rich in cysteine, and specific to *H*. *vastatrix* (Methods S5). For obtaining biological confirmation of the secretion prediction made by bioinformatics tools, the secretion of effector candidate proteins from *H*. *vastatrix* was confirmed in yeast (Methods S6). Information about the intron–exon structure of a select group of effector candidate genes was accessed by aligning the genomic DNA and cDNA sequences (Methods S7). Expression profiles of candidate *H*. *vastatrix* effector genes were accessed by RT‐PCR and real‐time quantitative polymerase chain reaction (RT‐qPCR) (Methods S8, S9). Methods for functional analysis of effectors using EDV system and *Psgc* 1202 are described in Methods S10–S12. All *H. vastatrix* effector candidate sequences predicted have been deposited in the NCBI database (GenBank) with the accession numbers KX670432–KX670525.

## Results

### Candidate effectors predicted from the pre‐biotrophic phase of the *H. vastatrix* infection cycle

**Figure 1:**
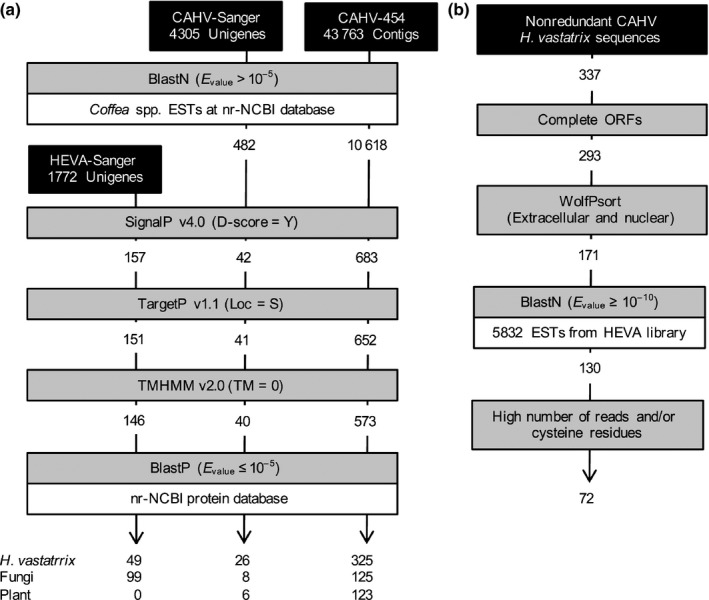
Bioinformatics pipeline used to predict the *Hemileia vastatrix* secretome. (a) Analysis of sequences obtained from HEVA and CAHV libraries made from germinated urediniospores of isolate Hv‐01, and from leaves of Catuaí Vermelho IAC 44 (*Coffea arabica*) infected with Hv‐01, respectively. (b) Analysis of unique sequences obtained from cDNA libraries made from the biotrophic phase of the infection cycle (after filtering out sequences obtained from germinated urediniospores and common sequences found in both CAHV‐Sanger and CAHV‐454 libraries). ORF, open reading frame.

Expressed sequence tags from germinating urediniospores at 16 h of incubation (Fig. S1) were used for the cDNA library construction named HEVA‐Sanger (Fig. 1; Notes S1). In total, 146 proteins that contained a secretion signal and no transmembrane domains were predicted. Of the 146 predicted secreted proteins, 49 (33.6%) have no significant similarity (BlastP *E*‐value >10^−5^) to sequences deposited in the nonredundant NCBI (nr‐NCBI) databases (Fig. 1a). Fifty‐eight predicted proteins (39.7%) show significant similarity with proteins of unknown function, including *Melampsora larici‐populina* secreted proteins (Table S1). Twenty‐nine predicted proteins (19.9%) show similarity with hydrolytic enzymes, including glycoside hydrolases, chitin deacetylase, carbohydrate esterases and proteases (Table S1). Ten (6.9%) predicted proteins show similarity to proteins with other functions, including superoxide dismutase and disulfide isomerase. Two sequences (1.4%) were deposited in the nr‐NCBI databases already as *H. vastatrix* proteins; these correspond to HvEC‐033 and HvEC‐045 (Table S2).

The 49 proteins that do not show significant similarity to any sequence deposited in the nr‐NCBI databases can correspond to secreted proteins unique to *H. vastatrix*. We hypothesized that these proteins are candidate effectors expressed during the prebiotrophic phase of the *H. vastatrix* lifecycle and therefore we named them *H. vastatrix* effector candidates (HvECs). We define HvECs as secreted proteins with no significant similarity to amino acid sequences present in other organisms, that is, proteins identified only in *H. vastatrix*. Among the 49 proteins with no similarity to sequences in other organisms, 34 had full‐length sequences and vary from 71 to 289 amino acids; 17 contain four or more cysteine residues in the mature peptide sequence (Table S2). No significant Pfam domains (*E*‐value cut‐off 1 × 10^−5^) were found in these candidate effectors.

We used BlastN to compare the open reading frame (ORF) sequences of the 34 full‐length ESTs with those reported previously (Fernandez *et al*., 2012; Cristancho *et al*., 2014; Talhinhas *et al*., 2014) in order to identity novel candidate *H. vastatrix* effectors. Our analysis revealed that HvEC‐032 and HvEC‐120 (highlighted yellow in Table S2) were not reported by these authors. In addition, 15 ORFs show similarity with ESTs previously reported (Fernandez *et al*., 2012; Cristancho *et al*., 2014; Talhinhas *et al*., 2014), but the sequences reported by those authors were truncated (highlighted blue in Table S2). We also confirmed the full‐length sequence of 17 candidate effectors previously reported (not highlighted in Table S2).

### Candidate effectors predicted from the biotrophic phase of the *H. vastatrix* infection cycle

In order to obtain a substantial set of ESTs from the *H. vastatrix* biotrophic phase, we constructed and analyzed two independent cDNA libraries. We initially sequenced 12 290 clones from a cDNA library (CAHV‐Sanger) made from infected coffee leaves at 12 d post‐inoculation (dpi) using Sanger technology. We obtained 4305 unigenes with average size of 469 bp (Notes S1). We also sequenced a normalized cDNA library (CAHV‐454) obtained from infected leaves at different time points during infection (48 and 72 h post‐inoculation (hpi), and 9 dpi) using 454‐GS‐FLX technology. Sampling at different time points was designed in order to maximize the identification of transcripts at several early stages of the interaction. Assembly of 629 890 reads resulted in 43 763 contigs with an average size of 867 bp and an average depth of coverage of 5.3X (Notes S1).

Seventy‐five percent of contigs from the CAHV‐454 library and 70% of the unique sequences from the CAHV‐Sanger library show homology (*E*‐value < 10^−5^) with sequences from *Coffea* spp. (Fig. 1a). Sequences that do not show homology with coffee ESTs were subjected to a bioinformatics pipeline in order to predict HvECs. Fifty‐seven percent (325 sequences) of the 573 predicted secreted proteins from the CAHV‐454 library do not show similarity with proteins deposited in nr‐NCBI databases and can be considered unique to *H. vastatrix* (Fig. 1a). A total of 125 secreted proteins show similarity to fungal proteins, including two predicted to be similar to rust transferred protein (RTP1) from *Uromyces fabae* (Kemen *et al*., 2005). Our analysis revealed a third *H. vastatrix* protein with significant similarity to RTP1, but our pipeline did not detect it as a secreted protein. These three proteins are referred to hereafter as HvRTP‐01A (contig CAHV_rep_c13089), HvRTP‐01B (CAHV_c1569) and HvRTP‐01C (CAHV_c5217), respectively. Three *H. vastatrix* proteins predicted to be secreted show similarity to haustorially expressed secreted proteins (HESPs) from *M. lini* (Catanzariti *et al*., 2006). These predicted secreted proteins included two similar to HESP‐178 and one similar to HESP‐379 (Table S3).

Bioinformatics analysis of the secretome predicted in both biotrophic libraries (CAHV‐454 and CAHV‐Sanger combined; no similarity in the nr‐NCBI database) (Fig. 1b) allowed the identification of 130 full‐length ORFs that encode soluble and secreted proteins with no similarity to any of the 5832 ESTs originated from germinated urediniospores and presumably expressed *in planta*. In order to establish a prioritization scheme to undertake functional analysis of the predicted effectors, we selected the 72 sequences with the higher number of reads per contig and/or sequences that code for cysteine‐rich proteins. PCR amplification using cDNA synthesized from RNA obtained from different structures of the fungus, as well as using genomic DNA, demonstrated that 13 of the 72 sequences were amplified from noninoculated plants and were considered as nonannotated coffee genes (Notes S2). The other 60 sequences (including two variants of *HvEC‐004*) were amplified exclusively from fungal DNA and were considered authentic *HvEC*s (Notes S2; Table S4). Primer sequences and amplicon sizes are shown in Tables S5 and S6.

**Figure 2:**
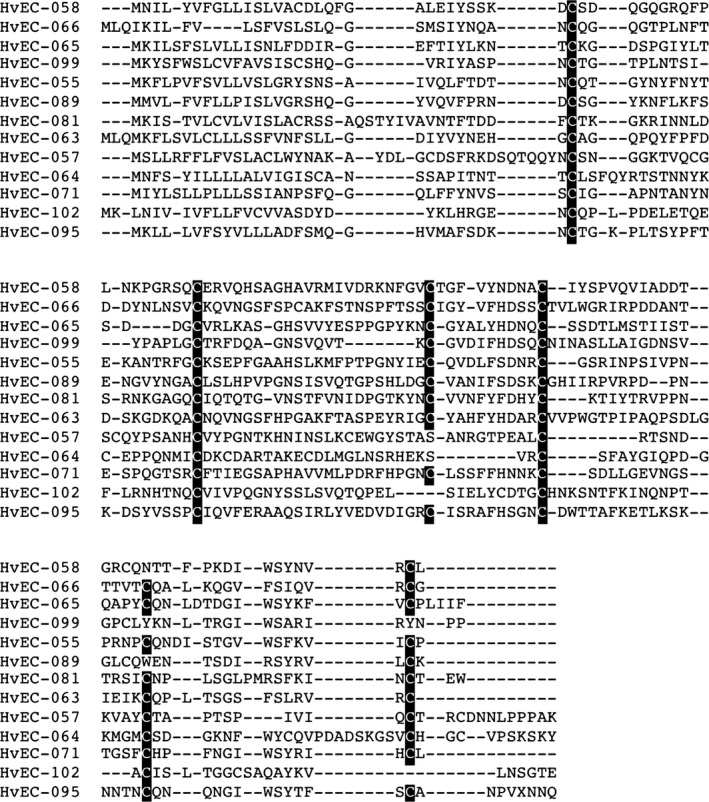
Alignment of 13 complete amino acid sequences from cysteine‐rich HvECs exhibiting a regular spacing pattern of cysteine residues. Cysteine residues are shown in black boxes. Alignment was done using Kalign2 (Lassmann *et al*., 2009).

Thirty‐seven of the 60 HvECs contain > 4 cysteine residues and 30 HvECs contain ≥ 5% cysteine residues in their predicted protein sequences (Table S4). Thirteen HvECs whose mature peptide contain > 5% cysteine residues and whose size range from 107 to 126 amino acids showed conservation of at least four of their cysteine residues (Fig. 2). We also included within the selected HvECs five (HvEC‐002, HvEC‐006, HvEC‐053, HvEC‐054, and HvEC‐074) that do not contain cysteine residues in their mature peptides (Table S4) taking into account that not all effector proteins are rich in cysteine residues (Dodds *et al*., 2004; Catanzariti *et al*., 2006; Gout *et al*., 2006; Bowen *et al*., 2009). None of the selected candidate effectors contain Pfam domains in their primary sequences, except for HvEC‐056 whose N‐terminus shows significant similarity (*E*‐value = 7.8 ×10^−6^) to the CFEM (PF05730) domain, which is composed of eight invariably spaced cysteine residues and is specific to fungi (Kulkarni *et al*., 2003).

In order to validate our prediction of the secretion signal, seven selected candidate effectors from the HEVA‐Sanger library and 11 from the CAHV‐libraries were cloned *in frame* with the invertase sequence in the expression vector pYST‐1 (Lee *et al*., 2006) and screened for secretion in yeast. *Saccharomyces cerevisiae* BY4742 (YIL162w) expressing the chimeric invertase grew on selective medium containing sucrose as the sole carbon source (Fig. S2), demonstrating that the predicted secretion signals are functional in yeast.

### Expression profiles of candidate *H. vastatrix* effectors

We used RT‐PCR in order to identify candidate *H. vastatrix* effectors expressed preferentially during its interaction with the coffee plant. Each of the 60 *HvEC*s selected from the biotrophic phase libraries was amplified by PCR from samples obtained from dormant and germinated urediniospores, as well as from infected coffee leaves collected at 24 hpi and 12 dpi using gene‐specific primers (Notes S2). In addition, we evaluated the expression *in planta* of the three genes coding for proteins similar to RTP1 identified in the *H. vastatrix* transcriptome. Our results showed that 35 *HvEC*s and *HvRTP‐01C* accumulated transcripts in fungal structures during prebiotrophic and biotrophic phases (Notes S2). Twenty‐four *HvEC*s, *HvRTP‐01A* and *HvRTP‐01B* had transcript accumulation exclusively and/or preferentially during the interaction of the fungus with the plant (Notes S2).

**Figure 3:**
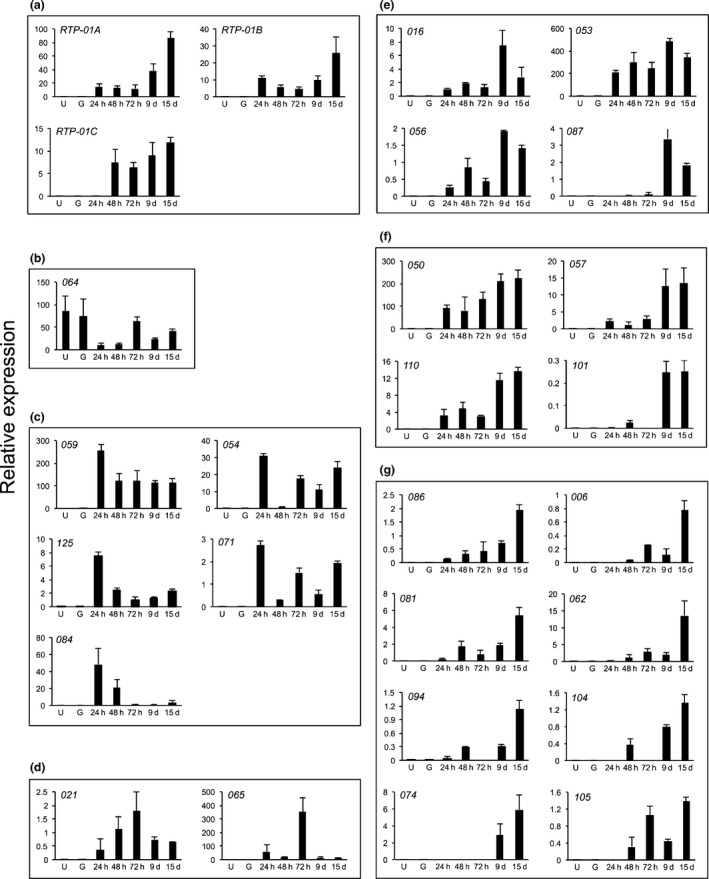
Expression patterns of a select group of candidate *Hemileia vastatrix* effectors from biotrophic phase libraries. Transcript accumulation was determined by real‐time quantitative polymerase chain reaction (RT‐qPCR) in samples obtained from dormant urediniospores (U), urediniospores germinated on polystyrene plates (G), infected leaves at 24 h, 48 h and 72 h post‐inoculation (hpi), and 9 d and 15 d post‐inoculation (dpi). The candidate effectors in (a) were grouped based on homology and in (b–g) based on their highest peak of transcript accumulation. (a) Candidate effectors coding for proteins similar to RTP1 from *Uromyces fabae* (Kemen *et al*., 2005). (b) *HvEC* with highest peaks in dormant and germinated urediniospores. (c) *HvECs* with highest peaks at 24 hpi. (d) *HvECs* with highest peak at 72 hpi. (e) *HvECs* with highest peak at 9 dpi. (f) *HvECs* with highest peaks at 9 and 15 dpi. (g) *HvECs* with highest peak at 15 dpi. Bars represent mean relative expression (transcript accumulation of the *HvEC* normalized using the mean transcript accumulation of the *H. vastatrix* endogenous genes cytochrome c oxidase subunit III, glyceraldehyde‐3‐phosphate dehydrogenase, and β‐tubulin). Vertical bars indicate SEM relative expression. Effector names are indicated using a simplified nomenclature without the initial *HvEC*.

**Figure 4:**
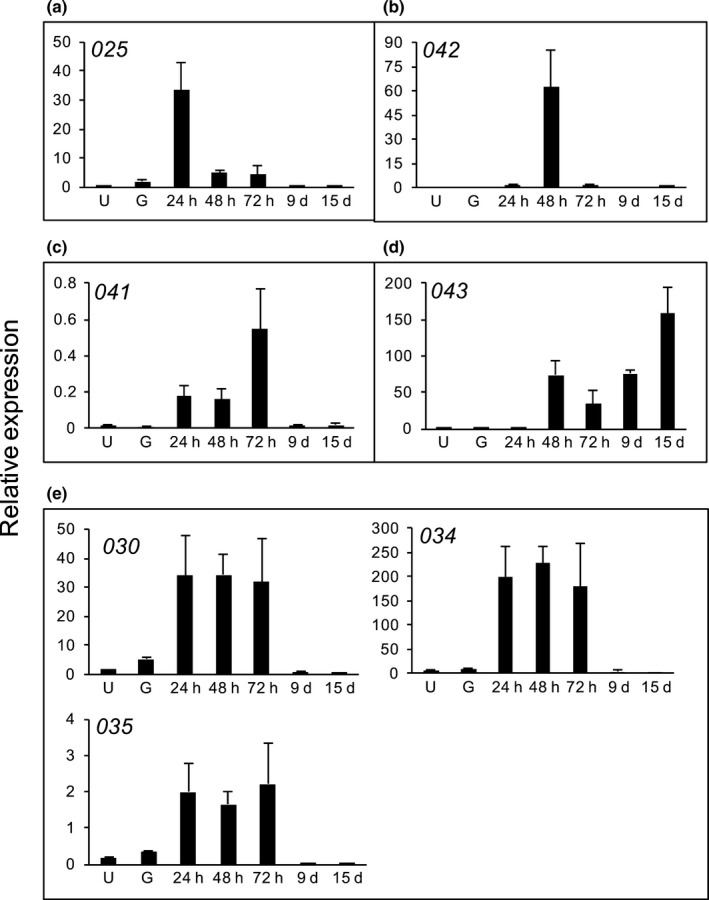
Expression patterns of seven *HvECs* from the germinated urediniospores library. Transcript accumulation was determined by real‐time quantitative polymerase chain reaction (RT‐qPCR) in samples obtained from dormant urediniospores (U), urediniospores germinated on polystyrene plates (G), infected leaves at 24 h, 48 h and 72 h post‐inoculation (hpi), 9 d and 15 d post‐inoculation (dpi). The candidate effectors were grouped based on their highest peak of transcript accumulation: (a) highest peak at 24 hpi; (b) highest peak at 48 hpi; (c) highest peak at 72 hpi; (d) highest peak at 15 dpi; and (e) highest peaks at 24, 48 and 72 hpi. Bars represent mean relative expression (transcript accumulation of the *HvEC* normalized using the mean transcript accumulation of the *Hemileia vastatrix* endogenous genes cytochrome c oxidase subunit III, glyceraldehyde‐3‐phosphate dehydrogenase, and β‐tubulin). Vertical bars indicate SEM relative expression. Effector names are indicated using a simplified nomenclature without the initial *HvEC*.

In order to investigate the expression profile of these 24 *HvEC*s, *HvRTP‐01A*,* HvRTP‐01B* and *HvRTP‐01C*, we ran RT‐qPCR on samples collected from three biological replicates of infected plants at 24, 48 and 72 hpi, and 9 and 15 dpi. We also included in our analysis samples from dormant and germinated urediniospores (Fig. 3). Expression profiles observed for *HvRTP1* homologs were similar to those previously reported for *H. vastatrix* (Fernandez *et al*., 2012) and consistent with differential expression observed for *M*. *larici*‐*populina RTP1* homologs (Hacquard *et al*., 2012) (Fig. 3a). *HvEC*s showed different expression patterns and could be assigned to six groups according to their peaks of highest transcript accumulation: in urediniospores (Fig. 3b); at 24 hpi (Fig. 3c); at 72 hpi (Fig. 3d); at 9 dpi (Fig. 3e); at 9 and 15 dpi (Fig. 3f); and at 15 dpi (Fig. 3g). In general, the selected *HvEC*s did not show transcript accumulation in urediniospores (except for *HvEC‐064*). Therefore, they were confirmed to be associated with the biotrophic phase of the *H. vastatrix* infection cycle. Because *HvEC‐064* showed an expression profile that indicates that it is preferentially expressed in urediniospores, we subjected to RT‐qPCR the seven *HvEC*s identified in germinated urediniospores and whose secretion was validated in yeast (Fig. S2; Table S2). Our results indicate that their expression is further induced *in planta* (Fig. 4).

### Intron–exon structure of *H. vastatrix* HvEC genes

Aimed at determining whether genes coding for candidate effectors contain introns, we amplified 35 of them by PCR from *H. vastatrix* Hv‐01 genomic DNA. We obtained amplicons ranging from 216 (*HvEC‐043*) to 1014 (*HvEC‐016*) nucleotides (Notes S3). We obtained two amplicons for *HvEC‐035* (375 and 625 bp, respectively) indicating the presence of at least two homologous genes in the *H. vastatrix* Hv‐01 genome. In order to determine the intron–exon junctions, the ORFs predicted from cDNA were aligned with the genomic sequences (Notes S4). Twenty‐four of the 35 genomic sequences analyzed have introns, most of them (15 sequences) with two introns (Notes S3, S4). Canonic GT/AC splicing sites were identified flanking 50 predicted introns, which corresponds to 84.7% of the total number of introns analyzed (Notes S4). The *HvEC‐016* gene consists of five exons (Notes S4). The number of exons observed in *HvEC‐016* is consistent with the mean exon number per gene identified in complete genome sequences of the rust fungi *M. larici*‐*populina* and *Puccinia graminis* f. sp. *tritici* (Duplessis *et al*., 2011).

### *Pseudomonas syringae* pv. *garcae* recognizes T3SS signals and secretes adenylate cyclase into plant cells

We established an effector translocation system based on the EDV system (Sohn *et al*., 2007). In this system, the candidate effector is fused *in frame* to the secretion signal of the *P. syringae* effector AvrRps4 under the control of the *avrRps4* promoter.

**Figure 5:**
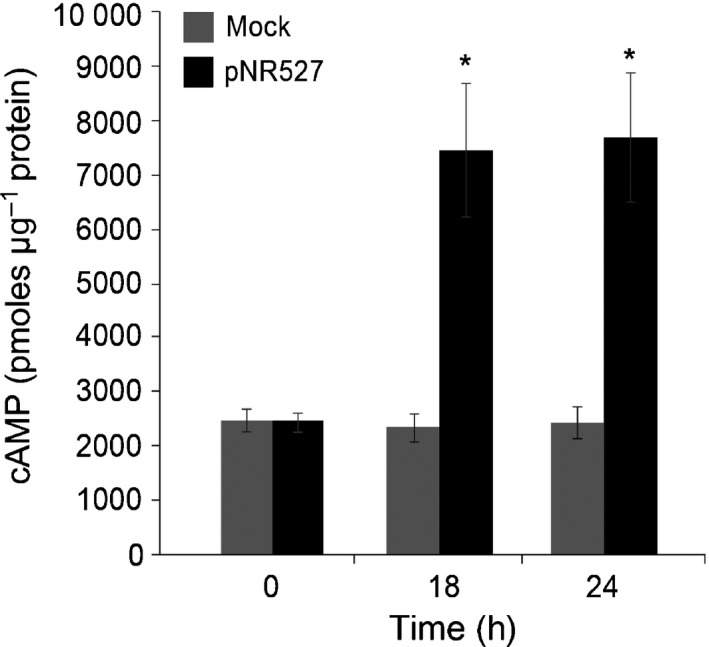
*Pseudomonas syringae* pv. *garcae* (*Psgc*) 1202 translocates a heterologous reporter protein into *Coffea arabica* cells via the T3SS. Coffee leaf samples were taken at 0, 18 and 24 h post‐infiltration with suspensions at 4 × 10^7^ colony forming units (CFU) ml^−1^ of *Psgc* 1202 carrying the pNR527 expression vector. The mock treatment consisted of infiltration of 10 mM MgCl_2_ without bacteria. Intracellular cAMP in coffee leaves was determined using ELISA. Bars represent mean of three biological replicates and the vertical bars indicate ± SE. Asterisks indicate statistically significant differences between the two means within the same time point (Student's *t*‐test, *P *<* *0.05). The experiment was repeated two times with similar results.

We first investigated whether *Psgc* 1202 was able to translocate the AvrRpm1 T3SS::CyaA fusion expressed in pNR527 (Upadhyaya *et al*., 2014) into cells of Catuaí Vermelho IAC 44 plants. We did not use the T3SS signal of AvrRps4 to test adenylate cyclase (CyaA) translocation due to the nucleocytoplasmic localization of this effector in the plant cell (Wirthmueller *et al*., 2007; Upadhyaya *et al*., 2014). We therefore used the T3SS signal of AvrRpm1 because the colocalization of this effector with calmodulin in the plasma membrane makes it appropriate for CyaA‐based translocation assays (Upadhyaya *et al*., 2014). A significant accumulation of cAMP was observed in leaves infiltrated with *Psgc* 1202 (pNR527) at 18 and 24 hpi, whereas low levels were detected in leaves inoculated with 10 mM MgCl_2_ (Fig. 5). These results demonstrate that *Psgc* 1202 is able to recognize the AvrRpm1 T3SS signals and to translocate effector proteins into cells of coffee plants using this signal sequence.

### HvEC‐016 attenuated the disease symptoms caused by *P. syringae* pv. *garcae* in coffee plants carrying the *S*_*H*_1 resistance gene

**Figure 6:**
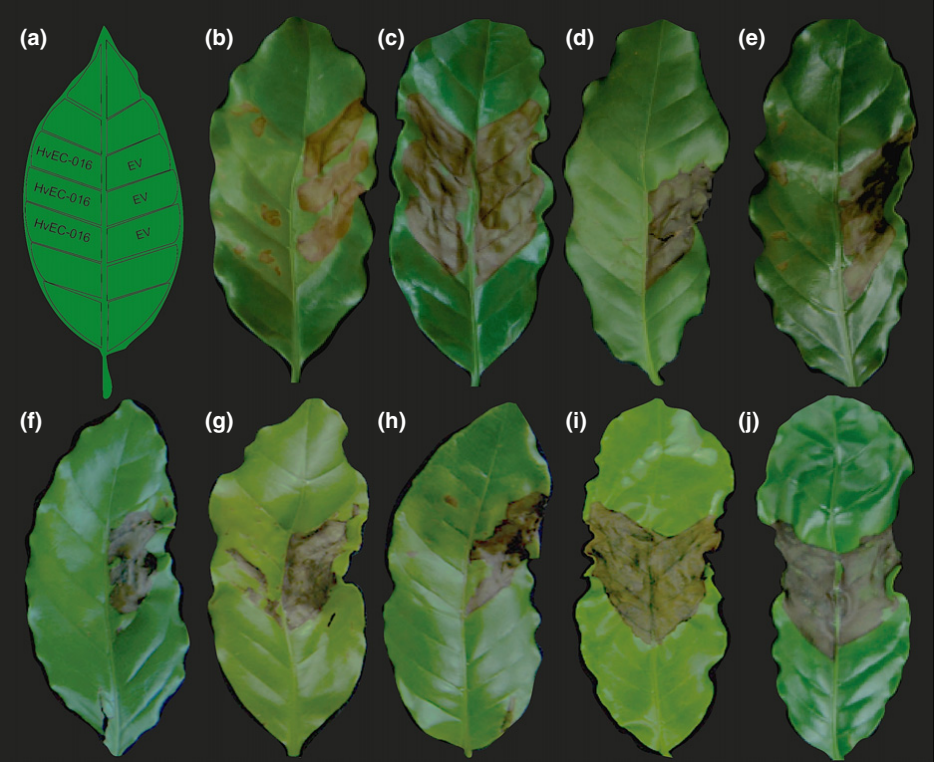
HvEC‐016 suppresses necrosis caused by *Pseudomonas syringae* pv. *garcae* (*Psgc*) 1202 in coffee genotypes carrying the *S*_*H*_1 resistance gene. Symptoms observed in leaves of coffee genotypes carrying different rust resistance genes from a subset of differentials used to identify *Hemileia vastatrix* races. (a) Schematic representation of coffee leaf infiltration with *Psgc* 1202 expressing either pEDV6::*HvEC‐016* (left panel) or empty pEDV6 (right panel). (b) 128/2 Dilla & Algue, resistance gene *S*_*H*_1. (c) ‘Catuaí Vermelho IAC 44’, resistance gene *S*_*H*_5. (d) 134/4 S.12 Kaffa, resistance genes *S*_*H*_1*,*4. (e) 87/1 Geisha, resistance genes *S*_*H*_1*,*5. (f) 1006/10 KP 532, resistance genes *S*_*H*_1*,*2*,*5. (g) H153/2, resistance genes *S*_*H*_1*,*3*,*5. (h) HW 17/12, resistance genes *S*_*H*_1*,*2*,*4*,*5. (i) H147/1, resistance genes *S*_*H*_2*,*3*,*4*,*5. (j) H420/10, resistance genes *S*_*H*_5*,*6*,*7*,*9. Leaves were infiltrated with bacterial suspensions at 2 × 10^7^ colony forming units (CFU) ml^−1^. Pictures were taken at 6 d post‐infiltration. The experiment was conducted more than three times with similar results.

We tested the hypothesis that some HvECs delivered by the *Psgc* 1202 T3SS into coffee genotypes can alter the normal phenotype caused by the bacterium in an *S*_*H*_‐dependent manner. We infiltrated *Psgc* 1202 expressing individual *HvEC*s into coffee genotypes used to differentiate *H. vastatrix* races, each genotype containing one or different combinations of known *S*_*H*_ genes (Table 1). We delivered 30 effector candidates using the EDV system and found that only HvEC‐016 consistently and reproducibly suppressed or attenuated the disease symptoms caused by *Psgc* 1202 on coffee genotypes carrying the *S*_*H*_1 resistance gene (Fig. 6). The mature HvEC‐016 is a protein of 201 amino acids, containing seven cysteine residues (Table S4). When the coffee genotypes Catuaí, H147/1 and H420/10 were challenged with *Psgc* 1202 expressing *HvEC‐016*, we observed the development of water‐soaked lesions at 48 hpi, and then tissue necrosis similar to bacterial blight of coffee, which began to develop 72 hpi (Fig. 6c,i,j). By contrast, the same bacterial suspension was unable to cause symptoms in coffee genotypes containing the resistance gene S_*H*_1, alone or in combination with other *S*_*H*_ genes (Fig. 6b,d–h).

**Table 1:** Differential reaction of coffee genotypes toward *Hemileia vastatrix* Hv‐01, *Psgc* 1202 (pEDV6), and *Psgc* 1202 (pEDV6::*HvEC‐016*)

Coffee genotypes^a^	*S*_*H*_ genes	Hv‐01	*Psgc* 1202	
			pEDV6	pEDV6::*HvEC‐016*
128/2 – Dilla & Algue	**1**	R	S	**R**
Bourbon – ‘Catuaí’	5	S	S	S
1343/269 – H. Timor	6	R	S	S
134/4 – S 12 Kaffa	**1**,4	R	S	**R**
87/1 – Geisha	**1**,5	R	S	**R**
32/1 – DK 1/5	2,5	R	S	S
33/1 – S 288‐23	3,5	R	S	S
110/5 – S 4 Agaro	4,5	R	S	S
1006/10 – KP 532	**1**,2,5	R	S	**R**
H153/2	**1**,3,5	R	S	**R**
H152/3	2,4,5	R	S	S
H419/20	5,6,9	R	S	S
HW 17/12	**1**,2,4,5	R	S	**R**
H147/1	2,3,4,5	R	S	S
H420/10	5,6,7,9	R	S	S
644/18 ‐ H. Kawisari	?	R	S	S

The experiment was performed three times with similar results.

a Coffee genotypes used as differentials for *H. vastatrix* races from the Plant Pathology Department at the Universidade Federal de Viçosa. S, susceptible; R, resistant; ?, *S*_*H*_ genes that have not been identified. S*_H_*1 gene and its respective phenotype, when challenged by *Psgc* expressing HvEC‐016, are in bold.

### HvEC‐016 restricts multiplication of *P. syringae* pv. *garcae* in coffee genotypes carrying the *S*_*H*_1 resistance gene

**Figure 7:**
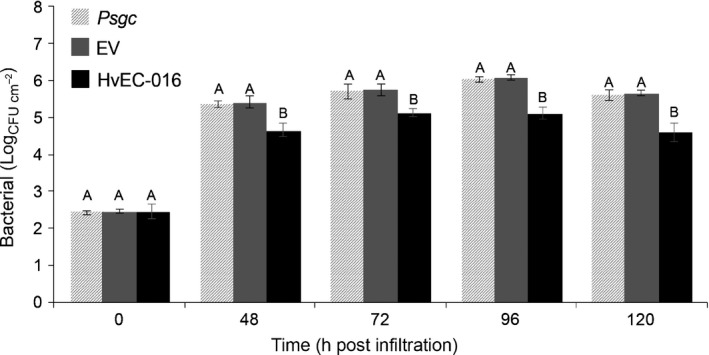
HvEC‐016 suppresses the ability of *Pseudomonas syringae* pv. *garcae* (*Psgc*) 1202 to multiply in *S*_*H*_1‐containing Dilla & Algue plants (*Coffea arabica*). Leaves were infiltrated with bacterial suspensions of *Psgc* 1202 wild‐type (light grey bars), *Psgc* 1202 expressing the empty vector (dark grey bars) or *Psgc* 1202 expressing pEDV6::*HvEC‐016* (black bars) at 1 × 10^4^ CFU ml^−1^. Bars represent mean of bacterial numbers recovered from three biological replicates of infected leaves at the indicated time points after infiltration (h post‐inoculation, hpi). Vertical lines indicate ± SE. For each time point, means labelled with different letters are significantly different (ANOVA and Tukey's test; *P *<* *0.05). The experiment was conducted three times with similar results.

We then investigated whether the suppression of necrosis caused by expression HvEC‐016 in coffee genotypes carrying *S*_*H*_1 was associated with restriction of bacterial multiplication *in planta*. We infiltrated leaves of the coffee genotype Dilla & Algue, which only expresses the *S*_*H*_1 resistance gene, with *Psgc* 1202 carrying pEDV6::*HvEC‐016* and compared its population with those reached by *Psgc* 1202 wild‐type (WT) and *Psgc* 1202 carrying the empty vector at different time points between 0 and 120 hpi. Although we did not observe any rapid development of macroscopic tissue collapse suggestive of HR elicitation in *S*_*H*_1‐containing plants inoculated with *Psgc* 1202 expressing *HvEC‐016*, HvEC‐016 caused a significant reduction in the bacterial population at all time points (Fig. 7). The bacterial numbers in leaves inoculated with *Psgc* 1202 expressing *HvEC‐016* were approximately one log lower than in those inoculated with *Psgc* 1202 WT or *Psgc* 1202 expressing the empty vector. These results suggested a possible association between the phenotypic change caused by HvEC‐016 and expression of *S*_*H*_1 in the plant, resulting in increased resistance against bacterial infection. These results demonstrate that our experimental approach of effector delivery into the coffee cytoplasm using the EDV system was successful for the identification of candidate *H. vastatrix* effectors potentially recognized by the plant immune system.

**Figure 8:**
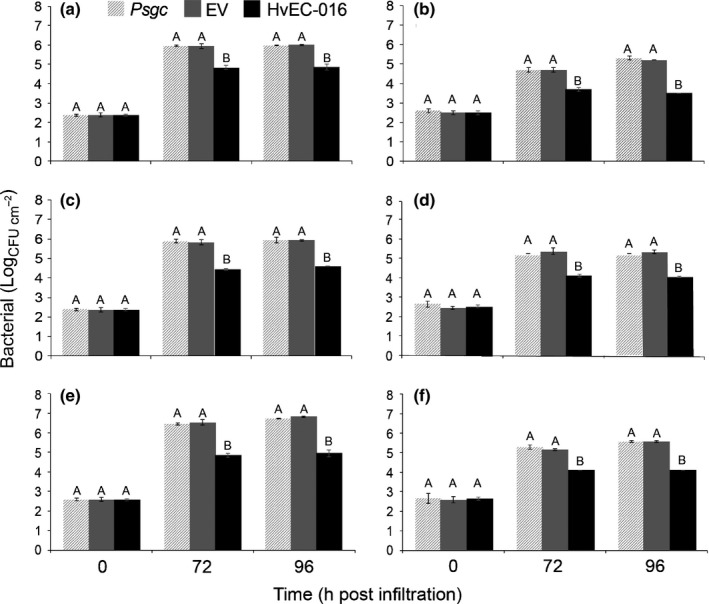
HvEC‐016 restricts multiplication of *Pseudomonas syringae* pv. *garcae* (*Psgc*) 1202 in *S*_*H*_1‐carrying coffee genotypes with different genetic backgrounds. Leaves were infiltrated with bacterial suspensions of *Psgc* 1202 wild‐type (light grey bars), *Psgc* 1202 carrying empty vector (dark grey bars), and *Psgc* 1202 expressing pEDV::*HvEC‐016* (black bars) at 1 × 10^4^ colony forming units (CFU) ml^−1^. Bars represent mean of bacterial numbers recovered from three biological replicates of infected leaves at the indicated time points after infiltration (h post‐inoculation, hpi). Vertical lines indicate errors of the mean values. For each time point, means labelled with different letters are significantly different (ANOVA and Tukey's test; *P *<* *0.05). (a) 134/4 S.12 Kaffa (*S*_*H*_1,4); (b) 87/1 Geisha (*S*_*H*_1,5); (c) 1006/10 KP 532 (*S*_*H*_1,2,5); (d) H153/2 (*S*_*H*_1,3,5); (e) 635/3 (*S*_*H*_1,4,5); (f) HW 17/12, (*S*_*H*_1,2,4,5). The experiment was repeated two times with similar results.

In order to further scrutinize a possible association between the restriction in bacterial multiplication caused by *HvEC‐016* and *S*_*H*_1, we inoculated 134/4 S.12 Kaffa, 87/1 Geisha, 1006/10 KP 532, H153/2, 635/3 and HW 17/12, six additional coffee accessions with different genetic backgrounds, but expressing *S*_*H*_1. These plant accessions were classified previously into different coffee physiological groups (Notes S5). In all cases, the number of bacterial cells recovered from leaves inoculated with *Psgc* 1202 (pEDV6::*HvEC‐016*) were significantly lower than those recovered from leaves inoculated with *Psgc* 1202 WT or *Psgc* 1202 carrying the empty vector (Fig. 8). We observed that different accessions sustain different levels of bacterial growth. For instance, although the highest bacterial numbers recovered from 134/4 S.12 Kaffa, 1006/10 KP 532 and 635/3 were in the order of 10^6 ^colony forming units (CFU) ml^−1^, those recovered from 87/1 Geisha, H153/2 and HW 17/12 were in the order of 10^5^ CFU ml^−1^, suggesting that these genotypes have different levels of basal resistance against *Psgc* 1202 (Fig. 8). Nonetheless, such a difference in basal resistance did not compromise the restriction of bacterial multiplication caused by HvEC‐016. These results strongly suggest that the restriction of necrosis caused by HvEC‐016 is associated with expression of *S*_*H*_1.

### HvEC‐016 enhances multiplication of *P. syringae* pv. *garcae* in coffee genotypes lacking the *S*_*H*_1 resistance gene

**Figure 9:**
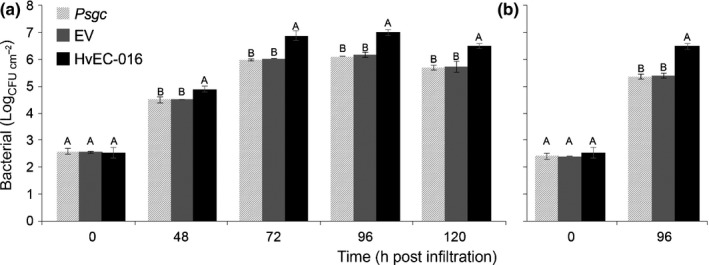
HvEC‐016 enhances the ability of *Pseudomonas syringae* pv. *garcae* (*Psgc*) 1202 to multiply in *S*_*H*_1‐lacking coffee genotypes (*Coffea arabica*). (a) ‘Catuaí Vermelho IAC 44’ (*S*_*H*_5). (b) ‘Caturra’ (*S*_*H*_5). Leaves were infiltrated with bacterial suspensions of *Psgc* 1202 wild‐type (light grey bars), *Psgc* 1202 carrying empty vector (EV, dark grey bars), or *Psgc* 1202 expressing pEDV::*HvEC016* (black bars) at 1 × 10^4^ colony forming units (CFU) ml^−1^. Bars represent mean of bacterial numbers recovered from three biological replicates of infected leaves at the indicated time points after infiltration (h post‐inoculation, hpi). Vertical lines indicate ± SE values. For each time point, means labelled with different letters indicate statistically significant difference (ANOVA and Tukey's test; *P *<* *0.05). The experiment was conducted three times with similar results.

We investigated subsequently whether HvEC‐016 could enhance the ability of *Psgc* 1202 to multiply in coffee plants lacking the *S*_*H*_1 resistance gene. We inoculated Catuaí plants with *Psgc* 1202 expressing *HvEC‐016* and compared its bacterial population to those reached by *Psgc* 1202 WT and *Psgc* 1202 expressing the empty vector at several time points after infiltration. Catuaí contains *S*_*H*_5, but not *S*_*H*_1. HvEC‐016 caused a significant increase of the bacterial cell numbers at all time points compared with the control strains (Fig. 9a). This enhancement of bacterial multiplication does not seem to be associated to the *S*_*H*_‐independent genetic background because it was also caused by HvEC‐016 in Caturra plants, which also only carry *S*_*H*_5 (Fig. 9b). These observations indicate that HvEC‐016 contributes to *Psgc* 1202 multiplication *in planta*. We did not detect any alteration in the severity of the disease symptoms caused by *Psgc* 1202 expressing *HvEC‐016* in *S*_*H*_1‐lacking plants as compared with the control strains. Expression of *HvEC‐016* in *Psgc* 1202 does not cause any change in the ability of the bacterium to grow *in vitro*, demonstrating that the observed plant phenotypes are not associated with altered bacterial physiology (Fig. S3).

## Discussion

We set out to undertake an alternative approach that combined conventional and high‐throughput sequencing technologies to generate high‐quality data and to predict the *H. vastatrix* secretome from transcriptome data. We also implemented a tissue sampling strategy to increase the chances to obtain effector sequences from several different stages of the coffee rust infection. Here we report a total of 94 *Hemileia*‐specific candidate effector genes, referred to as *HvEC*s, of which 14 have not been reported previously. We provide for 34 earlier partial effectors full‐length sequences, and confirm 46 effector sequences already published (Fernandez *et al*., 2012; Cristancho *et al*., 2014; Talhinhas *et al*., 2014). We identified genes encoding proteins with significant similarity to the previously reported RTP1 (Kemen *et al*., 2005; Vieira *et al*., 2012; Pretsch *et al*., 2013) and HESP proteins (Dodds *et al*., 2006; Barrett *et al*., 2009) in the *H. vastatrix* secretome, providing validation for our prediction.

Most *HvEC*s genes selected for further characterization in this study encode amino acid sequences enriched in cysteine residues. Thirteen of the HvECs showed similar cysteine spacing patterns. In *M. larici‐populina* several effector families subject to positive selection were shown to have similar cysteine spacing patterns despite low amino acid similarity among their members (Hacquard *et al*., 2012). The disulfide bonds are likely necessary to maintain the protein structure, whereas the variable regions can provide the sequences required to play specific effector functions (Hacquard *et al*., 2012). In this scenario, significant changes in the amino acid sequences other than in the position of the cysteine residues can be tolerated without altering the folding topology, making cysteine‐rich proteins good candidates for involvement in recognition and specificity (Templeton *et al*., 1994; Povolotskaya & Kondrashov, 2010). A high number of cysteine residues could play an important role in effector evolution allowing a rapid diversification and contributing to the emergence of new pathotypes. Among the rust fungi, *H. vastatrix* is the species with the longest latent period (*c*. 20 d between infection and sporulation). In addition, its pustules can produce urediniospores for up to 5 months (McCain & Hennen, 1984). A highly diversified repertoire of *H. vastatrix* effectors may be associated with defence suppression in order to establish an intimate relationship with the coffee plant for long periods of time.

In agreement with the lack of recognizable domains in most effector proteins previously predicted in other fungal pathogens, we detected a Pfam domain in only one of the candidate *H. vastatrix* effectors. Screening of 19 avirulence proteins for Pfam domains showed that only the *Cladosporium fulvum* effectors Avr4 and Ecp6 have significant similarity with Pfam domains, Chitin Binding Module (PF14776) and LysM (PF01476) domains, respectively (Saunders *et al*., 2012). The presence of functional domains in effector proteins can aid in deciphering their functions. For instance, it is now well established that Avr4 and Ecp6 are chitin‐binding proteins that play important roles in hyphal protection and perturbation of chitin‐triggered immunity during *C. fulvum* infection, respectively (van den Burg *et al*., 2006; van Esse *et al*., 2007; de Jonge *et al*., 2010). However, the CFEM domain (acronym for common in several fungal extracellular membrane) found in candidate effector HvEC‐056 is not associated with a known biochemical activity or overall sequence similarity, but with the positions of eight conserved cysteine residues in the mature peptide (Kulkarni *et al*., 2003). Although the biochemical function for this domain has not been elucidated, CFEM‐containing proteins have been identified in the secretomes of several plant pathogenic fungi, including *Sclerotinia sclerotiorum* (Guyon *et al*., 2014), *Cronartium quercuum* f. sp. *fusiforme* (Pendleton *et al*., 2014), *Fusarium virguliforme* (Abeysekara & Bhattacharyya, 2014) and *Melampsora* spp. (Joly *et al*., 2010).

Transcript accumulation profiles indicated that expression of most *HvEC*s analysed in this study are observed as early as 24 hpi and remain active until 15 dpi. Histology observations of compatible interactions between *H. vastatrix* and *Coffea* plants have shown that at 24 hpi the fungal structures most frequently observed in infection sites are appressoria, primary hyphae and primary haustoria; at 48 and 72 hpi most infection sites are occupied by haustorial mother cells and secondary haustoria; and at 15 dpi, 90% of infection sites are occupied by secondary haustoria (Matsuoka & Vanetti, 1993; Silva *et al*., 2002, 2008; Ganesh *et al*., 2006; Ramiro *et al*., 2009; Vieira *et al*., 2012). It is tempting to speculate that *H. vastatrix* begins to modulate the host physiology as early as during penetration of stomata, with many effectors being secreted from primary hyphae, appressoria and/or primary haustoria. Other effectors, like those induced at later times, may be haustorium‐specific because their induction is correlated with the presence of these structures in infected tissue. Yet, other effectors expressed at late stages could prime urediniospores for their encounter with the coffee leaf surface. The expression patterns of *HvEC*s are very variable with effectors showing highest peaks at different time points after infection, suggesting that they play key roles at different stages during the infection process. In any case, the majority of *HvEC*s analyzed in this study were expressed preferentially (highest peaks of relative transcript accumulation), presumably after the differentiation of haustoria in the mesophyll. Expression of effectors *HvEC‐050*,* HvEC‐053* and *HvEC‐059* was high throughout the whole infection process, suggesting that high gene expression levels of these effectors are required to establish and maintain a biotrophic lifestyle. *Hemileia vastatrix* genes coding for proteins similar to RTP1 showed different patterns of transcript accumulation suggesting that they can be expressed from different fungal structures as well. Immunofluorescence microscopy studies demonstrated that RTP1 homologs from *M*. *larici*‐*populina* are expressed not only in the haustorium as previously reported for *U. fabae* (Kemen *et al*., 2005), but also in intercellular hyphae in the mesophyll layer of the plant tissue (Hacquard *et al*., 2012). More recently, Kemen *et al*. (2013) showed that the RTP1 protein exhibits variable localization in the extra‐haustorial matrix and in the interior of the host cell during infection. It is intriguing that expression of *HvRTP‐01C*, which was not detected by our bioinformatics pipeline as a secreted protein, is strongly induced at 48 hpi and remains high until 15 dpi. Lack of a signal for secretion in HvRTP1‐01C suggests that it could also play a role during the infection process from within the fungal cell to stabilize and protect the haustorium from degradation.

We successfully adapted the EDV system (Sohn *et al*., 2007) for delivery of candidate *H. vastatrix* effector proteins into coffee cells. *Psgc* 1202 translocated adenylate cyclase by recognition of the T3SS signal of AvrRpm1 present in the chimera of the well‐characterized construct pNR527 (Upadhyaya *et al*., 2014). This result is consistent with the presence of T3SS genes in the recently published draft genome sequence of *Psgc* ICMP 4323 (Thakur *et al*., 2016). Furthermore, a gene coding for a protein exhibiting 74.2% identity to AvrRps4 is also present in the *Psgc* ICMP 4323 genome. The T3SS signal sequence of this protein shows 67% identity to that of AvrRps4, supporting the notion that *Psgc* delivers an AvrRps4‐family protein into coffee cells during infection.

Delivery of a select group of HvECs into coffee plants using the EDV system revealed that HvEC‐016 suppresses the necrosis caused by *Psgc* in genotypes containing the *S*_*H*_1 resistance gene. The suppression of necrotic symptoms in *S*_*H*_1‐genotypes was associated with a decrease in bacterial multiplication. Both suppression of necrotic symptoms and restriction of bacterial growth were observed in coffee accessions belonging to different physiological groups, but carrying the *S*_*H*_1 gene. We observed that a higher level of basal resistance against *Psgc* 1202 infection in some coffee accessions did not compromise the reduction in bacterial cell numbers caused by HvEC‐016 in *S*_*H*_1 plants. These results suggest that HvEC‐016 may be recognized by the immune system of the coffee plant in an *S*_*H*_ *1*‐dependent manner, halting the development of the necrotic symptom characteristic of bacterial blight, and making this *H. vastatrix*‐secreted protein a good candidate for avirulence protein. Nonetheless, our results do not allow us to rule out the possibility that the suppression of bacterial blight symptoms could be due to the involvement of a yet unidentified factor present in the *S*_*H*_ *1*‐carrying plant genotypes. Currently, due to unavailability of genetic transformation methods for both coffee and *H*. *vastatrix* it will be difficult to corroborate this hypothesis. The *S*_*H*_1 gene has not yet been cloned and thus, conventional transient coexpression assays in other plant species, such as *Nicotiana benthamiana*, are not possible at the moment. Notably, *H. vastatrix* isolate 178a (race XIV), which is also avirulent on *S*_*H*_ *1* plants, carries the same allele of *HvEC‐016* as that of isolate Hv‐01 (race II) (Fernandez *et al*., 2012; Talhinhas *et al*., 2014). Also, consistent with the expression profile observed in our study, *HvEC‐016* was identified in all three libraries of isolate 178a (interaction, appressoria and germinating urediniospores) screened by these authors. An alternative interpretation for the suppression of necrotic symptoms in *S*_*H*_1 plants is that HvEC‐016 suppresses the activity of some *Psgc* 1202 effectors rendering the bacterium less virulent. This idea is consistent with reports indicating that effectors from pathogens belonging to different kingdoms interact with the same subset of highly connected cellular hubs (protein complexes) to promote parasitism (Mukhtar *et al*., 2011). Our observations that *Psgc* 1202 expressing *HvEC‐016* supresses necrotic symptoms and reduces bacterial multiplication in *S*_*H*_1 plants with diverse genetic backgrounds and that HvEC‐016 contributes to *Psgc* 1202 multiplication in *S*_*H*_1‐lacking genotypes not only rule out a possible interference of HvEC‐016 with the function of bacterial effectors, but also strengthen the possibility of this candidate being a *bona fide H. vastatrix* effector. To support this finding, we are currently developing large coffee populations segregating for *S*_*H*_1. These populations will be phenotyped for both resistance to Hv‐01 and HvEC‐016 suppression of bacterial blight symptoms. In addition, we are trying to obtain naturally occurring strains or EMS‐induced mutants of *H. vastatrix* able to overcome *S*_*H*_1 resistance. Cosegregation of resistance to Hv‐01 with HvEC‐016 suppression of bacterial blight, as well as identification of sequence polymorphisms in *HvEC‐016* alleles associated with breaking of *S*_*H*_1 resistance will allow us to conclude that *HvEC‐016* is indeed an *H. vastatrix* avirulence gene corresponding to *S*_*H*_1.

The EDV system has been used widely to functionally characterize effectors from a variety of filamentous plant pathogens (Sohn *et al*., 2007; Cabral *et al*., 2011; Fabro *et al*., 2011; Goritschnig *et al*., 2012; Badel *et al*., 2013; Caillaud *et al*., 2013; Sharma *et al*., 2013; Liu *et al*., 2016). These bacterial delivery systems translocate effectors directly into the cytoplasm of plant cells in which they play their biological roles. Hence, their recognition by resistance proteins must occur inside the plant cell. Most cytoplasmic R proteins identified so far are nucleotide‐binding site and leucine rich repeat proteins (NBS‐LRR) (Rafiqi *et al*., 2009; Takken & Tameling, 2009). Using bacterial delivery systems, Goritschnig *et al*. (2012) identified the *Hevea arabidopsidis* avirulence gene *ATR39* among a large set of candidate effectors and were able to clone its cognate resistance gene *RPP39*, coding for a CC‐NBS‐LRR protein. Moreover, a candidate avirulence effector (PGTAUSPE‐10‐1) from *P. graminis* f. sp. *tritici* was identified using a similar bacterial delivery system based on *P. fluorescens* EtHAn. PGTAUPE‐10‐1 elicited an HR specifically on wheat line W3534 expressing the resistance gene *Sr22* (Upadhyaya *et al*., 2014). Analysis of the *S*_*H*_ *3* locus in *C. arabica* demonstrated the presence of candidate genes coding for CC‐NBS‐LRR proteins (Ribas *et al*., 2011), supporting the notion that *H. vastatrix* avirulence proteins are recognized in the cytoplasm of coffee cells. Several studies have demonstrated that ETI can be reconstructed by co‐expression of cognate avirulence and resistance genes in heterologous plants (van der Hoorn *et al*., 2000; Abramovitch *et al*., 2003; Armstrong *et al*., 2005). Cloned *H. vastatrix* avirulence genes may be used as molecular probes to detect specific phenotypic interactions with candidate coffee NBS‐LRRs in heterologous systems speeding up the cloning of corresponding resistance genes. Also, heterologous expression of avirulence proteins will allow to determine their subcellular localization (Rafiqi *et al*., 2010; Petre *et al*., 2015) and to identify their potential plant targets (Selin *et al*., 2016; Toruño *et al*., 2016).

## Author contributions

S.H.B., T.M. and J.L.B. planned and designed the research experiments; T.M., G.M‐R., C.d.M.R., M.B.F., J.C.F.d.S. and G.M.d.A‐J. performed experiments; T.M., J.L.B. and S.H.B. analysed data; and T.M. and J.L.B. wrote the manuscript.

## Supporting information

Please note: Wiley Blackwell are not responsible for the content or functionality of any Supporting Information supplied by the authors. Any queries (other than missing material) should be directed to the *New Phytologist* Central Office.

**Fig. S1 **Light microscopy pictures showing urediniospores of *Hemileia vastatrix* Hv‐01 germinated on polystyrene plates for 16 h.
**Fig. S2** Secretion of HvECs in yeast.
**Fig. S3** HvEC‐016 does not alter multiplication of *Pseudomonas syringae* pv. *garcae* 1202 *in vitro*.
**Methods S1** Urediniospores germination.
**Methods S2** HEVA‐Sanger library construction and sequence analysis.
**Methods S3** CAHV‐Sanger library construction and sequence analysis.
**Methods S4** CAHV‐454 library construction and sequence analysis.
**Methods S5** Secretome prediction.
**Methods S6** Yeast secretion assay.
**Methods S7** Identification of *HvEC*s introns.
**Methods S8** Nonquantitative RT‐PCR.
**Methods S9** Quantitative RT‐PCR.
**Methods S10** Bacterial strains and EDV constructions.
**Methods S11** Plant material and assays.
**Methods S12** CyaA assay.
**Notes S1 **Sequencing statistics for cDNA libraries from *Hemileia vastatrix* prebiotrophic and biotrophic phases of the infection cycle.
**Notes S2** Fungal origin and expression of candidate *Hemileia vastatrix* effectors.
**Notes S3** Number of introns of a select group of *HvEC* genes.
**Notes S4 **Alignment of genomic and cDNA nucleotide sequences of candidate *Hemileia vastatrix* effector genes.
**Notes S5** Coffee genotypes carrying *S*_*H*_1 used to determine the effect of HvEC‐016 on multiplication of *Pseudomonas syringae* pv. *garcae* 1202.

**Table S1** Similarity of *Hemileia vastatrix* secreted proteins expressed in germinated urediniospores to other fungal proteins
**Table S2** Selected *HvEC*s with full‐length ORFs identified in the HEVA‐Sanger library
**Table S3** Predicted proteins from the *Hemileia vastatrix* secretome showing significant similarity to haustorially‐expressed proteins from other rust fungi
**Table S4** Selected *HvEC*s with full‐length ORFs identified in CAHV libraries
**Table S5 **Primers used to amplify full‐length *HvECs* from cDNA by PCR to validate the secretion of corresponding proteins in yeast, and/or from genomic DNA to determine their number of introns
**Table S6 **Primers used for RT‐PCR
