# Supplementary material for: The Hemileia vastatrix effector HvEC‐016 suppresses bacterial blight symptoms in coffee genotypes with the SH1 rust resistance gene
Source: New Phytol. 2016 Dec 5;213(3):1315–29. doi: 10.1111/nph.14334 (PMC6079635; doi:10.1111/nph.14334)
Supplement: Supplementary file 1 — Fig. S1 Light microscopy pictures showing urediniospores of Hemileia vastatrix Hv‐01 germinated on polystyrene plates for 16 h. Fig. S2 Secretion of HvECs in yeast. Fig. S3 HvEC‐016 does not alter multiplication of Pseudomonas syringae pv. garcae 1202 in vitro. Methods S1 Urediniospores germination. Methods S2 HEVA‐Sanger library construction and sequence analysis. Methods S3 CAHV‐Sanger library construction and sequence analysis. Methods S4 CAHV‐454 library construction and sequence analysis. Methods S5 Secretome prediction. Methods S6 Yeast secretion assay. Methods S7 Identification of HvECs introns. Methods S8 Nonquantitative RT‐PCR. Methods S9 Quantitative RT‐PCR. Methods S10 Bacterial strains and EDV constructions. Methods S11 Plant material and assays. Methods S12 CyaA assay. Notes S1 Sequencing statistics for cDNA libraries from Hemileia vastatrix prebiotrophic and biotrophic phases of the infection cycle. Notes S2 Fungal origin and expression of candidate Hemileia vastatrix effectors. Notes S3 Number of introns of a select group of HvEC genes. Notes S4 Alignment of genomic and cDNA nucleotide sequences of candidate Hemileia vastatrix effector genes. Notes S5 Coffee genotypes carrying S H1 used to determine the effect of HvEC‐016 on multiplication of Pseudomonas syringae pv. garcae 1202. [file NPH-213-1315-s001.pdf]

New Phytologist Supporting Information Figs S1–S3, Methods S1–S12 and Notes S1–S5

Article title: The *Hemileia vastatrix* effector HvEC-016 suppresses bacterial blight symptoms in coffee genotypes with the S_H1 rust resistance gene

Authors: Thiago Maia, Jorge L. Badel, Gustavo M. Ramírez, Cynthia de M. Rocha, Michelle B. Fernandes, José C. F. da Silva, Gilson M. de Azevedo-Junior and Sérgio H. Brommonschenkel

Article acceptance date: 16 October 2016

The following Supporting Information is available for this article:

Fig. S1 Light microscopy pictures showing urediniospores of *Hemileia vastatrix* Hv-01 germinated on polystyrene plates for 16 h.

Fig. S2 Secretion of HvECs in yeast.

Fig. S3 HvEC-016 does not alter multiplication of *Pseudomonas syringae* pv. *garcae* 1202 *in vitro*.

Table S1 Similarity of *Hemileia vastatrix* secreted proteins expressed in germinated urediniospores to other fungal proteins (separate Excel file)

Table S2 Selected *HvEC*s with full-length ORFs identified in the HEVA-Sanger library (separate Excel file)

Table S3 Predicted proteins from the *Hemileia vastatrix* secretome showing significant similarity to haustorially-expressed proteins from other rust fungi (separate Excel file)

Table S4 Selected *HvEC*s with full-length ORFs identified in CAHV libraries (separate Excel file)

Table S5 Primers used to amplify full-length *HvECs* from cDNA by PCR to validate the secretion of corresponding proteins in yeast, and/or from genomic DNA to determine their number of introns (separate Excel file)

Table S6 Primers used for RT-PCR (separate Excel file)

Methods S1 Urediniospores germination.

Methods S2 HEVA-Sanger library construction and sequence analysis.

Methods S3 CAHV-Sanger library construction and sequence analysis.

Methods S4 CAHV-454 library construction and sequence analysis.

Methods S5 Secretome prediction.

Methods S6 Yeast secretion assay.

Methods S7 Identification of *HvEC*s introns.

Methods S8 Nonquantitative RT-PCR.

Methods S9 Quantitative RT-PCR.

Methods S10 Bacterial strains and EDV constructions.

Methods S11 Plant material and assays.

Methods S12 CyaA assay.

Notes S1 Sequencing statistics for cDNA libraries from *Hemileia vastatrix* prebiotrophic and biotrophic phases of the infection cycle.

Notes S2 Fungal origin and expression of candidate *Hemileia vastatrix* effectors.

Notes S3 Number of introns of a select group of *HvEC* genes.

Notes S4 Alignment of genomic and cDNA nucleotide sequences of candidate *Hemileia vastatrix* effector genes.

Notes S5 Coffee genotypes carrying S_H1 used to determine the effect of HvEC-016 on multiplication of *Pseudomonas syringae* pv. *garcae* 1202.

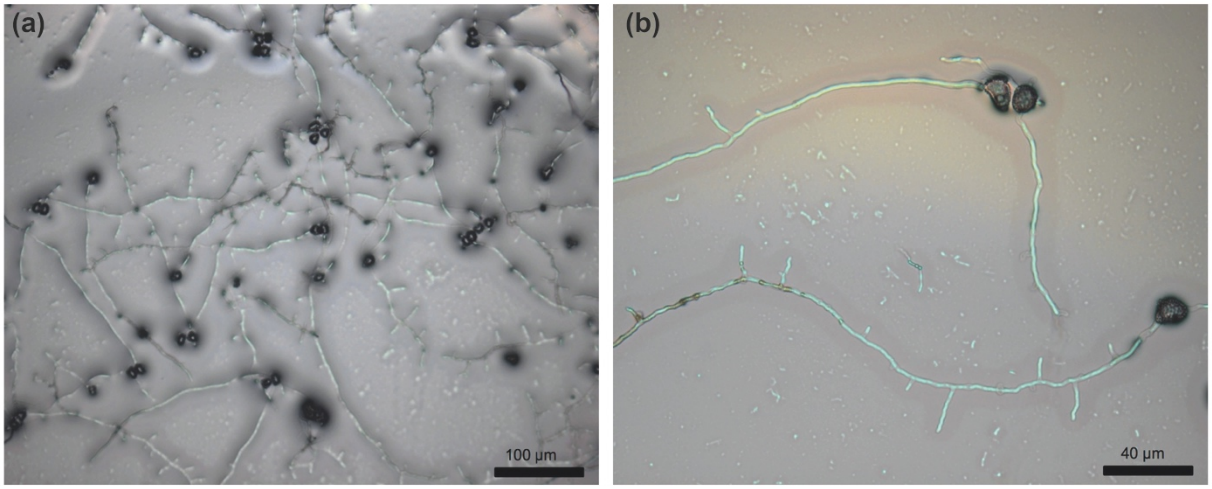

Fig. S1 Light microscopy pictures showing urediniospores of *Hemileia vastatrix* Hv-01 germinated on polystyrene plates for 16 h. (a) Urediniospores and germ tubes adhered to the bottom of the plate. (b) Long and branched germ tubes emerged from urediniospores.

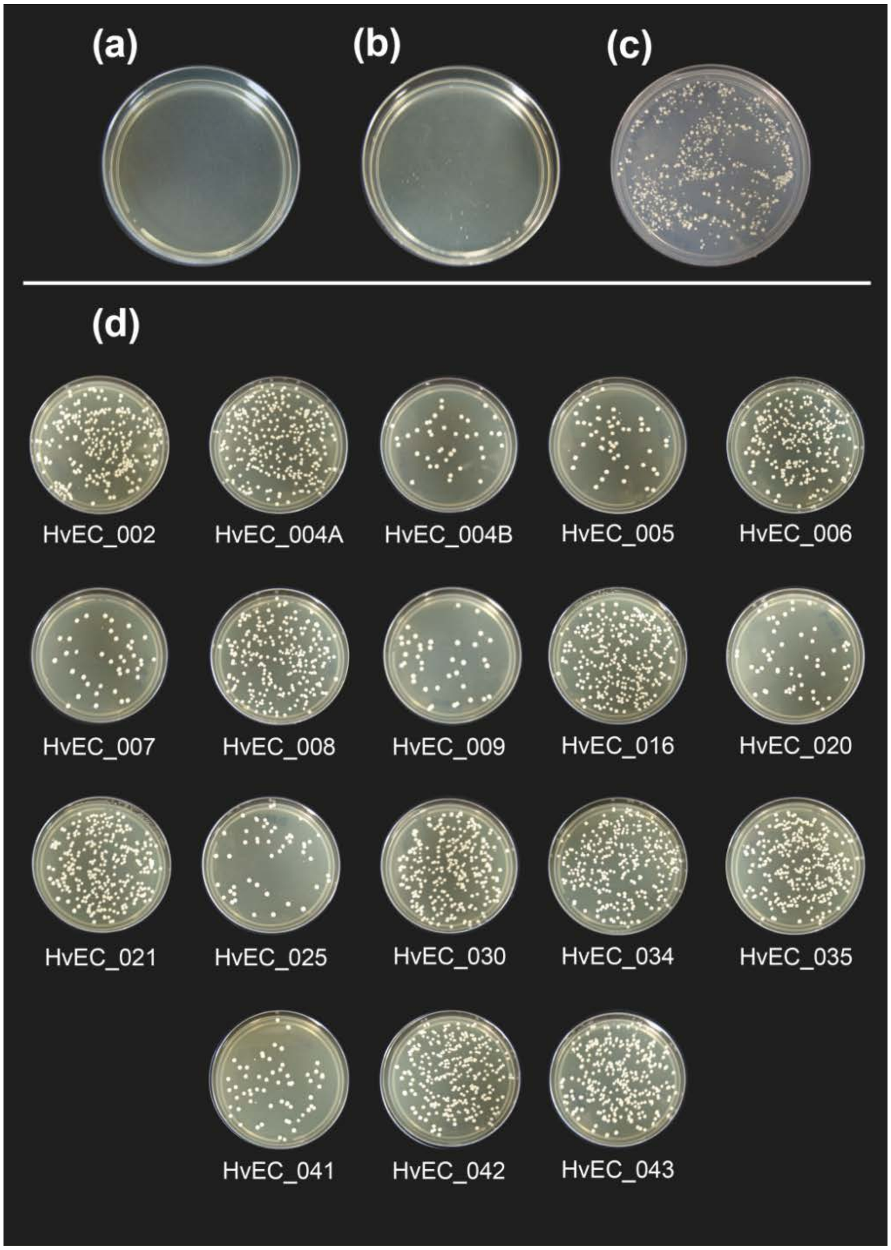

Fig. S2 Secretion of HvECs in yeast**.** (a) Nontransformed *Saccharomyces cerevisiae* strain BY4742 accession YIL162w (*MATα*, *SUC2*, *his3Δ*, *leu2Δ*, *lysΔ*, *ura3Δ*) plated on selective YPS medium (5.0 g l^{-1} yeast extract, 10.0 g l^{-1} peptone, 10% sucrose, 10.0 g l^{-1} agar). (b) Yeast transformed with empty pYST-1 vector plated on selective YPS medium. (c) Yeast transformed with empty pYST-1 plated on minimal medium SD-Leu. (d) Yeast transformants expressing selected *HvECs* cloned in frame with the invertase gene in vector pYST-1 grown on selective YPS medium.

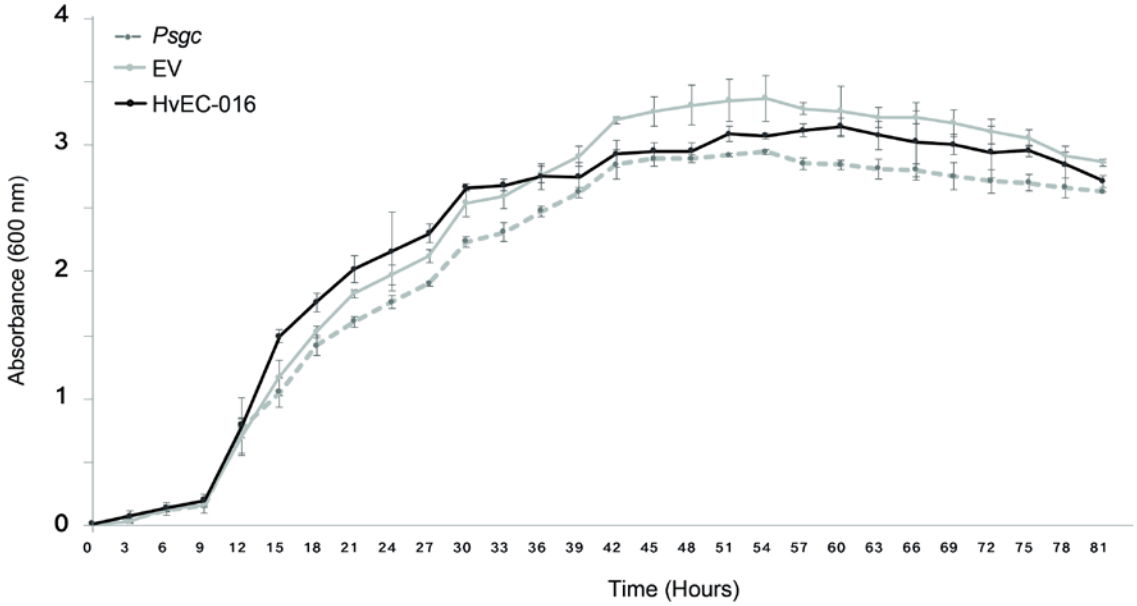

Fig. S3 HvEC-016 does not alter multiplication of *Pseudomonas syringae* pv. *garcae* 1202 *in vitro*. *Psgc* 1202 wild type (*Psgc*), *Psgc* 1202 carrying empty vector (EV) and *Psgc* 1202 expressing pEDV::*HvEC-016* (HvEC-016) were grown in liquid King's B medium amended with appropriate antibiotics. Optical density at 600 nm was measured at indicated times. Vertical lines indicate standard errors of three replicates. The experiment was conducted two times with similar results.

Methods S1 Urediniospores germination.

Single pustule *H. vastatrix* isolate Hv-01, previously characterized as race II in our laboratory, was multiplied on leaves of *C. arabica* var. Catuaí Vermelho IAC 44 (carrying S_H5). Two grams of fresh urediniospores were washed in sterile distilled water containing 0.05% Tween 80 (Sigma-Aldrich, St Louis, MO, USA). Urediniospores were homogeneously distributed in polystyrene Petri dishes containing a film of sterile distilled water and incubated at 22°C under darkness. Germination was confirmed by light microscopy at 16 hours of incubation. About 80% of urediniospores produced long germ tubes (Fig. S1). Germinated spores were collected, frozen in liquid nitrogen, and stored at -80°C until total RNA extraction.

Methods S2 HEVA-Sanger library construction and sequence analysis.

Total RNA was extracted from germinated urediniospores according to Bilgin *et al*. (2009). mRNA was purified from total RNA with the NucleoTrap® mRNA Midi Kit (Macherey-Nagel, Düren, Germany), according to the manufacturer's instructions. A unidirectional cDNA library was made using the Creator™ SMART™ cDNA library construction kit (Clontech, Mountain View, CA, USA) using 0.5 µg de polyA RNA. cDNA was digested with *Sfi*I (New England Biolabs, Beverly, MA, USA) and size-fractionated by electrophoresis. Digested fragments larger than 500 bp were ligated into pDNR-LIB (Clontech) and transformed into *Escherichia coli* EC-100 (Epicentre, Madison, WI, USA). A total of 7,200 recombinant clones were sequenced from the 5' end using the M13 universal primer. Sequencing reactions were carried out using the DYEnamicTM ET Dye Terminators kit (GE Healthcare, Pittsburgh, PA, USA), and readings were performed in an automatic MegaBACE 1000 Sequencing System (GE Healthcare). EST sequences with a Phred (Ewing & Green, 1998) score higher than 20 and longer than 70 nucleotides were retained for further analysis. Quality-trimmed sequences were assembled with the CAP3 assembler (Huang & Madan, 1999). A customized Perl script was used to predict ORFs.

Methods S3 CAHV-Sanger library construction and sequence analysis.

Hemileia vastatrix inoculum was prepared as previously described (Maia *et al*., 2013). Six-month-old Catuaí Vermelho IAC 44 plants were inoculated with fresh *H. vastatrix* Hv-01 urediniospores and samples of infected leaves were collected at 12 dpi. Unidirectional cDNA library construction and sequence analysis were performed essentially as described for the HEVA-Sanger library. A total of 9,828 recombinant clones were sequenced and 4,305 unigenes were obtained. Unigenes were compared with 254,546 ESTs of *Coffea* spp. deposited in nonredundant NCBI databases (www.ncbi.nlm.nih.gov/genbank) using BlastN (Altschul *et al*., 1997). Sequences with no significant homology (E-value < 10-5) with *Coffea* spp. ESTs were predicted their ORFs using a customized Perl script and included in further analysis.

Methods S4 CAHV-454 library construction and sequence analysis.

Total RNA was isolated from three biological replicates of Catuaí leaves infected with *H. vastatrix* Hv-01 at 48 and 72 hpi, and 9 dpi according to Bilgin *et al*. (2009). An equimolar mixture of RNA from all samples was precipitated and sent to Eurofins MWG Operon (Huntsville, AL, USA) for construction of a normalized random-primed cDNA library and pyrosequencing using the 454 GS-FLX Titanium platform (Roche, Branford, CT, USA). A total of 629,890 reads were grouped into 43,763 contigs using the MIRA assembler v3.4 (Chevreux *et al*., 2004). Singlets were not included in further analysis. The plant or fungal origin of the contigs was determined according to the procedures described for the CAHV-Sanger library. Six-frame ORF predictions were conducted using a customized Perl script.

Methods S5 Secretome prediction.

A bioinformatics pipeline was designed in order to predict secreted proteins of unknown function, rich in cysteine, and specific to *H. vastatrix*. Predicted amino acid sequences from each library (HEVA-Sanger, CAHV-454 and CAHV-Sanger) were initially subjected to SignalP v4.0 (Petersen *et al*., 2011) and then to TargetP v.1.1 (Emanuelsson *et al*., 2000) in order to select those carrying a signal peptide and lacking a mitochondrial targeting peptide. A customized Perl

script was used to remove the signal peptide and the sequences corresponding to the mature peptide were subjected to TMHMM v2.0 (Krogh *et al*., 2001) to filter out proteins containing transmembrane domains. Then, predicted secreted proteins from each library were queried against the nr-NCBI protein database using BlastP (Altschul *et al*., 1997) and those showing no significant similarity (E-value higher than 10^{-5}) were retained. Candidates containing full-length sequences were subjected to WolfPsort (Horton *et al*., 2007) and only sequences with extracellular and/or nuclear localization predictions were selected. Predicted proteins from CAHV-454 and CAHV-Sanger libraries were compared with each other using BlastP and those showing 100% identity were reduced to a single one. In addition, predicted proteins from CAHV-Sanger and CAHV-454 libraries showing significant similarity (E-value < 10^{-10}) with those from HEVA-Sanger were removed from the final set. The number of cysteine residues in each predicted mature peptide was determined using a customized Perl script. Significant Pfam domains (E-values lower than 10^{-5}) were scanned using the PFAM search server (Finn *et al*., 2010). ORF sequences from the final set of predicted effectors were compared with sequences previously reported (Fernandez *et al*., 2012; Cristancho *et al*., 2014; Talhinhas *et al*., 2014) using BlastN (Altschul *et al*., 1997).

Methods S6 Yeast secretion assay.

Full-length *HvEC* ORFs were amplified by PCR using forward and reverse HvEC-YST primers (Table S5) containing restriction sites for *Eco*RI and *Not*I, respectively. The amplicons were digested with *Eco*RI and *Not*I (Invitrogen, Carlsbad, CA, USA) and ligated into the pYST-1 vector (Lee *et al*., 2006) previously digested with the same enzymes. The ligation reactions were transformed into *E. coli* DH5α and transformants selected on solid LB containing ampicillin (150 μg ml^{-1}). Plasmid DNA was isolated from transformants using the NucleoSpin® Plasmid kit (Macherey-Nagel, Düren, Germany) and sequenced with primers Y-5 (5’-TCCTCGTCATTGTTCTCGTTCC-3’) and Y-3 (5’-CCTTTTATCCAAGCGGCC-3’) in order to confirm that the *HvECs* were in frame with invertase. *Saccharomyces cerevisiae* strain BY4742 accession YIL162w (*MAT*α, *SUC*2, *his*3Δ, *leu*2Δ, *lys*Δ, *ura*3Δ) was transformed with pYST-1::*HvEC* constructs or the empty vector according to Gietz & Woods (2002). Transformants were selected on solid YPS (5.0 g l^{-1} yeast extract, 10.0 g l^{-1} peptone, 10%

sucrose, 10.0 g l^{-1} agar) containing gentamicin (150 µg ml^{-1}). Control yeast strain carrying empty vector was also plated on synthetic defined medium lacking leucine (SD-Leu).

Methods S7 Identification of *HvECs* introns.

Genomic DNA from *H. vastatrix* Hv-01 was extracted from fresh urediniospores according to Maia *et al*. (2013). Forward and reverse HvEC-YST primers (Table S5) were used to amplify *HvECs* by PCR, and amplicons cloned into pGEM-T Easy (Promega, Madison, WI, USA) and sequenced using universal M13 primers. Intron-exon junctions were identified by aligning the genomic DNA and cDNA sequences using ClustalW (Thompson *et al*., 1994).

Methods S8 Nonquantitative RT-PCR.

Total RNA was isolated from dormant and germinated urediniospores, and from non-inoculated and inoculated coffee leaves at 24 hpi and 12 dpi (Bilgin *et al*., 2009). RNA was treated with RNase-free DNase I (Qiagen, Valencia, CA, USA) and purified using RNeasy CleanUp Kit (Qiagen). Ten micrograms of total RNA were used to synthesize cDNA using the Oligo(dT)12-18 primer with the SuperScript First-Strand Synthesis System for RT-PCR kit (Invitrogen, Carlsbad, CA, USA) in a total volume of 40 µl. The efficiency of cDNA synthesis was estimated using the endogenous genes ubiquitin and β-tubulin for coffee and *H. vastatrix*, respectively. Each RT-PCR reaction consisted of 94°C for 2 min, 30 cycles of 94°C for 30 s, 60°C for 30 s and 72°C for 30 s, followed by a final extension at 72°C for 5 min. Primer sequences and amplicon sizes are shown in Table S6.

Methods S9 Quantitative RT-PCR.

RNA isolation and cDNA synthesis were conducted essentially as described for RT-PCR with the same primer sets. Gene expression was quantified according to the comparative method $2^{-\Delta\Delta Ct}$ (Livak & Schmittgen, 2001). For quantification of gene expression at each time point (24, 48, and 72 h, and 9 and 15 d after inoculation) the mean Ct value resulting from three biological replicates and two technical replicates at each sampling time was used. The expression of each

candidate effector gene was then normalized with the three endogenous fungal genes glyceraldehyde-3-phosphate dehydrogenase (*HvGAPDH*), cytochrome c oxidase subunit III (*HvCytIII*) and β-tubulin (*Hvβ-tub*), which have been validated for quantification of coffee rust gene expression (Vieira *et al*., 2011). qPCR reactions were carried out in a 7500 Real Time PCR Systems (Applied Biosystems, Grand Island, NY, USA), programed for initial denaturation at 95°C for 10 min, followed by 40 cycles at 95°C for 15 s and 60°C for 30 s. Each reaction was run in a total volume of 20 µl containing 40 ng cDNA, 8 mM of each primer and 12 µl SYBR® Green PCR Master Mix (Applied Biosystems). The specificity of the amplification was determined using a dissociation curve with a gradient of temperature ranging from 60°C to 95°C at a rate of 1°C per 30 s.

Methods S10 Bacterial strains and EDV constructs.

HvECs without secretion sequences were cloned into pENTR D-TOPO (Invitrogen, Carlsbad, CA, USA) and then recombined into pEDV6 (Badel *et al*., 2013; Fabro *et al*., 2011) using the Gateway® LR Clonase™ II Enzyme Mix kit (Invitrogen) according to the manufacturer's instructions. In-frame fusions with the secretion signal of *AvrRps4* and integrity of the effector sequence of all pEDV6::*HvEC* constructs were confirmed by sequencing with universal M13 primers. pEDV6 plasmids expressing *HvECs* were mobilized from *E. coli* DH5α to *Psgc* 1202 by standard triparental mating using *E. coli* HB101 (pRK2013) as helper strain. Empty pEDV6 vector was maintained in *E. coli* DB3.1. Transformed *Psgc* 1202 cells were selected on solid King's B medium containing rifampicin (100 µg ml^{-1}) and gentamicin (25 µg ml^{-1}).

Methods S11 Plant material and assays.

Plants of coffee accessions differential for *H. vastatrix* physiological races used in infiltration assays were obtained by vegetative propagation by stake rooting and/or grafting using Catuaí Vermelho IAC 44 as rootstock. After 4 months of nursery, plants were transferred to the glasshouse when the coffee seedlings had two or three pairs of leaves and grown in 5-l plastic pots containing a mix of soil and manure supplemented by liquid fertilization. *Psgc* 1202 wild type and *Psgc* 1202 carrying pEDV6::*HvEC* constructs or empty vector were grown on solid

King's B medium containing appropriate antibiotics at 28°C overnight, washed once and resuspended in sterile 10 mM $MgCl_2$. In order to test whether expression of *HvECs* in *Psgc* 1202 could alter its ability to cause disease on coffee plants expressing a subset of S_H resistance genes, fully expanded tender leaves were infiltrated with bacterial suspensions at 2×10^7 CFU ml^{-1} using a needless syringe and symptoms evaluated daily until 10 dpi. The experiment was repeated at least three times with similar results. To determine bacterial multiplication *in planta* bacterial suspensions at 1×10^4 CFU ml^{-1} were infiltrated into fully expanded tender leaves of plants carrying or not the S_H1 resistance gene. Leaves from three independent plants were infiltrated with each bacterial strain. Four 1-cm diameter disks were collected from each infiltrated leaf at several time points after inoculation using a cork borer. Leaf disks were ground in 10 mM $MgCl_2$, the macerate serially diluted and plated on solid King's B medium containing appropriate antibiotics and the number of colony forming units (CFU) counted.

Methods S12 CyaA assay.

Plasmid pNR527 (expressing AvrRpm1 T3SS::Cya) (Upadhyaya *et al*., 2014) was transformed into *Psgc* 1202 by electroporation. *Psgc* 1202 (pNR527) was grown on solid King's B medium at 28°C for 24 h, the cells resuspended in 10 mM $MgCl_2$ and the suspension adjusted to 4×10^7 CFU ml^{-1}. The bacterial suspension was infiltrated into young fully expanded leaves of Catuaí Vermelho IAC 44 plants using a needless syringe. Leaves inoculated with 10 mM $MgCl_2$ were used as controls. One-centimetre diameter leaf discs were collected from the inoculated leaves at 0, 18, and 24 h after infiltration. The discs were frozen in liquid nitrogen and kept at -80°C until processing. Three independent samples, each consisting of four leaf discs, from different inoculated plants were analysed. cAMP extraction and protein quantification were conducted as previously described (Carper-Lindley *et al*., 2002; Upadhyaya *et al*., 2014) with some modifications. Briefly, leaf discs were macerated in liquid nitrogen, 325 µl 1.1 M HClO4 was added, the suspension vortexed and centrifuged at maximum speed for 10 min. Three hundred microlitres of supernatant was neutralized with 40 µl 6M K2CO3. The pellet was used for protein quantification using the Bio-Rad Protein Assay kit II (Bio-Rad, Hercules, CA, USA). The supernatant was centrifuged for 8 min at maximum speed and 200 µl were collected. Ten-

microlitre aliquots were used for cAMP quantification using the Cyclic AMP EIA kit (Cayman Chemical Company, Ann Arbor, MI, USA).

Notes S1 Sequencing statistics for cDNA libraries from *Hemileia vastatrix* pre-biotrophic and biotrophic phases of the infection cycle.

454 PYROSEQUENCING			
CAHV-454 (biotrophic phase)			
Total number of reads			719,563
Number of reads assembled in contigs			629,890
Number of contigs assembled			43,763
Mean number of reads per contig			14.4
Mean contig size			867 bp
Total number of bases analysed			38 Mb
Mean contig coverage			5.3X
SANGER SEQUENCING	Singlets	Contigs	Unigenes
HEVA (pre-biotrophic phase): 5,832 ESTs			
Number of sequences	1,101	671	1,772
Mean size (bp)	503	636	553
Number of bases analysed (Mb)	0.55	0.43	0.98
CAHV-Sanger (biotrophic phase): 9,828 ESTs			
Number of sequences	3,301	1,004	4,305
Mean size (bp)	421	626	469
Number of bases analysed (Mb)	1.4	0.6	2.0

Notes S2 Fungal origin and expression of candidate *Hemileia vastatrix* effectors.

HvECs identified in CAHV libraries were amplified by PCR using as template Hv-01 genomic DNA (D) and by RT-PCR using as template cDNA obtained from dormant (S) and germinated (G) urediniospores, non-inoculated leaves (NI), leaves at 24 hpi (24 h), and leaves at 12 dpi (12 d). Endogenous *H. vastatrix* genes β-tubulin (*HvβTub*), cytochrome c oxidase subunit III (*HvCytIII*) and gliceraldehyde-3-phosphate dehydrogenase (*HvGADPH*) were used as positive controls for *H. vastatrix* amplification. Pathogenesis-related protein 1b (*CaPR1b*) and ubiquitin (*CaUbq*) genes were used as positive controls for coffee amplification. Sterile Milli-Q water (W) was used as negative control for amplification. Among the 72 genes tested, 13 showed amplification from non-inoculated plants and were considered as non-annotated coffee genes (indicated by one asterisk). Twenty-four genes that showed stronger amplification from cDNA obtained from infected leaf tissue were subjected to RT-qPCR (indicated by two asterisks).

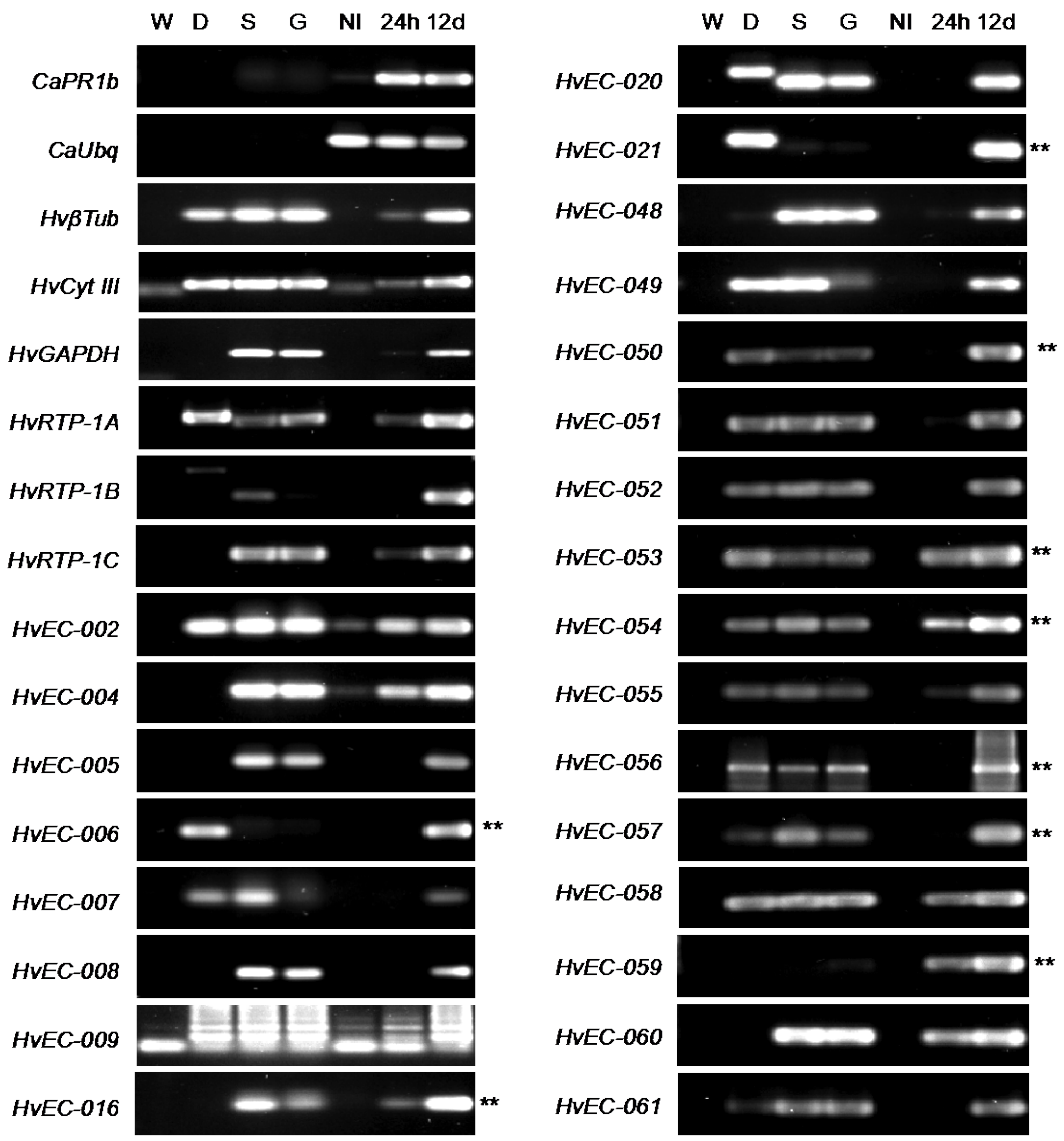

W D S G NI 24h 12d
CaPR1b
CaUbq
HvβTub
HvCyt III
HvGAPDH
HvRTP-1A
HvRTP-1B
HvRTP-1C
HvEC-002
HvEC-004
HvEC-005
HvEC-006 **
HvEC-007
HvEC-008
HvEC-009
HvEC-016 **
W D S G NI 24h 12d
HvEC-020
HvEC-021 **
HvEC-048
HvEC-049
HvEC-050 **
HvEC-051
HvEC-052
HvEC-053 **
HvEC-054 **
HvEC-055
HvEC-056 **
HvEC-057 **
HvEC-058
HvEC-059 **
HvEC-060
HvEC-061

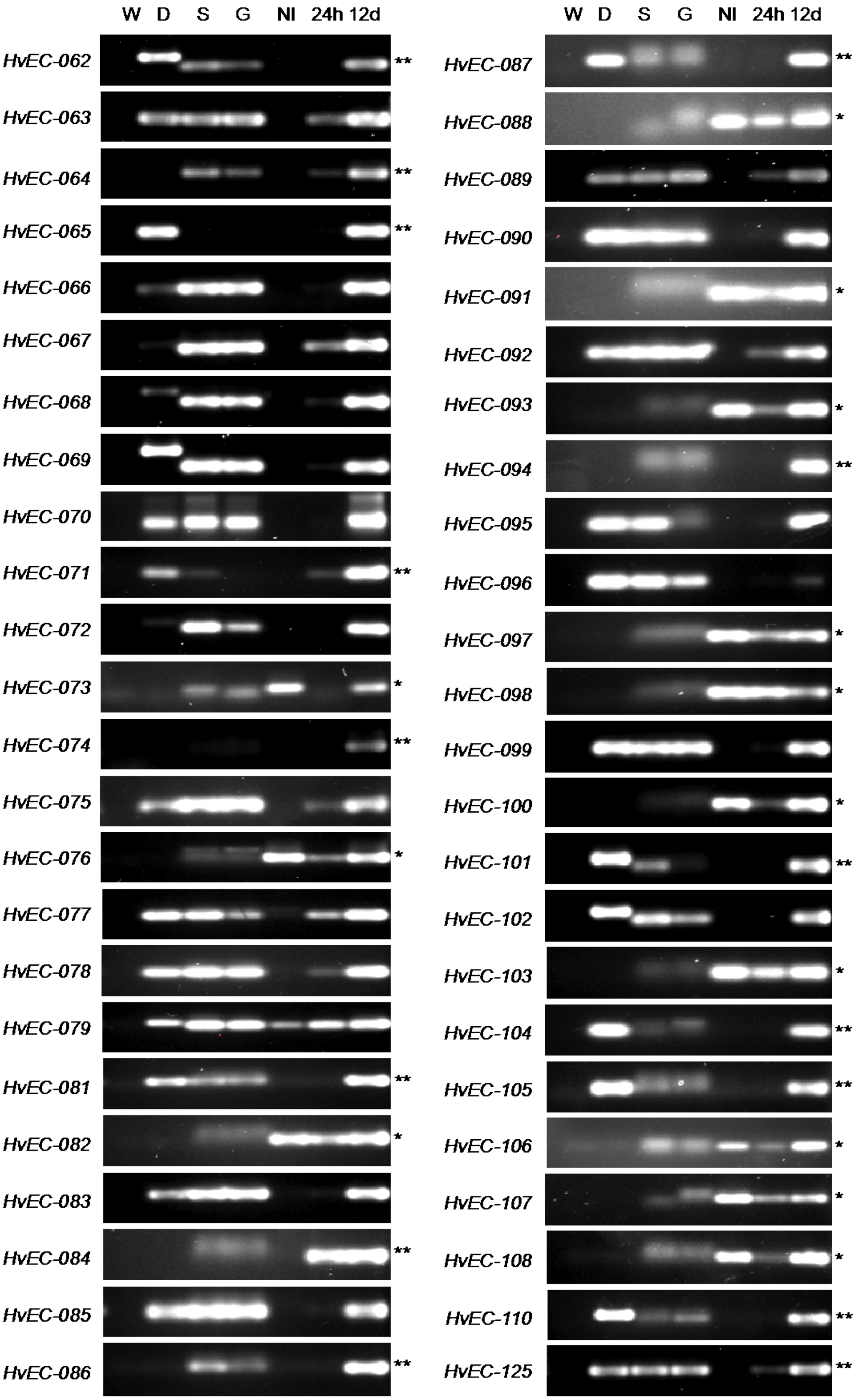

W D S G NI 24h 12d
HvEC-062 **
HvEC-063
HvEC-064 **
HvEC-065 **
HvEC-066
HvEC-067
HvEC-068
HvEC-069
HvEC-070
HvEC-071 **
HvEC-072
HvEC-073 *
HvEC-074 **
HvEC-075
HvEC-076 *
HvEC-077
HvEC-078
HvEC-079
HvEC-081 **
HvEC-082 *
HvEC-083
HvEC-084 **
HvEC-085
HvEC-086 **
W D S G NI 24h 12d
HvEC-087 **
HvEC-088 *
HvEC-089
HvEC-090
HvEC-091 *
HvEC-092
HvEC-093 *
HvEC-094 **
HvEC-095
HvEC-096
HvEC-097 *
HvEC-098 *
HvEC-099
HvEC-100 *
HvEC-101 **
HvEC-102
HvEC-103 *
HvEC-104 **
HvEC-105 **
HvEC-106 *
HvEC-107 *
HvEC-108 *
HvEC-110 **
HvEC-125 **

Notes S3 Number of introns of a select group of *HvEC* genes.

Candidate effector	cDNA size (bp)	Genomic DNA size (bp)	Number of introns
HvEC-002	294	294	0
HvEC-004A	582	706	2
HvEC-005	363	742	4
HvEC-006	396	396	0
HvEC-007	321	321	0
HvEC-008	345	885	2
HvEC-016	678	1014	4
HvEC-020	681	998	4
HvEC-021	585	857	4
HvEC-022	378	495	2
HvEC-024	246	384	2
HvEC-025	225	363	2
HvEC-026	225	360	2
HvEC-029	354	521	2
HvEC-030	384	582	2
HvEC-031	327	458	2
HvEC-032	249	399	2
HvEC-033	270	270	0
HvEC-034	327	459	2
HvEC-035A	375	375	0
HvEC-035B	375	625	3
HvEC-036	315	315	0
HvEC-037	264	332	1
HvEC-038	339	339	0
HvEC-039	270	350	1
HvEC-040	330	330	0
HvEC-041	339	338	0
HvEC-043	216	216	0
HvEC-045	384	663	4
HvEC-046	558	703	2
HvEC-047	450	557	2
HvEC-054	474	632	2

HvEC-057	381	707	4
HvEC-071	339	339	0
HvEC-087	609	770	2

Notes S4 Alignment of genomic and cDNA nucleotide sequences of candidate *Hemileia vastatrix* effector genes.

Red font indicates the canonical GT/AG splicing sites in exon-intron junctions. Alignments were done using ClustalW (Thompson *et al.*, 1994).

HvEC-002 alignment

```
CLUSTAL 2.1 multiple sequence alignment

  HvEC_gDNA_002      ATGAAGTTGACCCTATTTTTCATTGCGATTTTTGCATGTTTTTGCACTTTGGTGTCTTCC 60
  HvEC_cDNA_002      ATGAAGTTGACCCTATTTTTCATTGCGATTTTTGCATGTTTTTGCACTTTGGTGTCTTCC 60
                     ************************************************************

  HvEC_gDNA_002      ATCCCTGCTCCTGACACGGAAAACCACGTAAAGCCTGAAGGCGCCGATAAAAAATGGGGT 120
  HvEC_cDNA_002      ATCCCTGCTCCTGACACGGAAAACCACGTAAAGCCTGAAGGCGCCGATAAAAAATGGGGT 120
                     ************************************************************

  HvEC_gDNA_002      TTTCCCCCCTGGGTTTACCCTCTGTACAACACTTACCCCCTTTCCTATGACTCTCTCTAT 180
  HvEC_cDNA_002      TTTCCCCCCTGGGTTTACCCTCTGTACAACACTTACCCCCTTTCCTATGACTCTCTCTAT 180
                     ************************************************************

  HvEC_gDNA_002      CTCCGTCGATACTGGCCTAATTGGTGGAATTATATTCCAGCCGGATATGGAGGTGGAGTG 240
  HvEC_cDNA_002      CTCCGTCGATACTGGCCTAATTGGTGGAATTATATTCCAGCCGGATATGGAGGTGGAGTG 240
                     ************************************************************

  HvEC_gDNA_002      CTTTTGGATGCCAAGCAGCAAAATGCTAAAGCAAAAAGCCAAAAGCCAGAGTAA 294
  HvEC_cDNA_002      CTTTTGGATGCCAAGCAGCAAAATGCTAAAGCAAAAAGCCAAAAGCCAGAGTAA 294
                     ******************************************************
```

HvEC-004A alignment

```
CLUSTAL 2.1 multiple sequence alignment

  HvEC_gDNA_004A      ATGCAGTTCTCACTTTGCTCATTGCTGGCTCTGTTTGGTGGGTACTACCTTTCAAATCTT 60
  HvEC_cDNA_004A      ATGCAGTTCTCACTTTGCTCATTGCTGGCTCTGTTTG----------------------- 37
                      *************************************                       

  HvEC_gDNA_004A      CTGGTCCTCTCTCTGCTTACATTCCCTATCACTCTCAGCTTTCTTTTTCAGCGCCTCAGC 120
  HvEC_cDNA_004A      --------------------------------------CTTTCTTTTTCAGCGCCTCAGC 59
                                                            **********************

  HvEC_gDNA_004A      TTTGCCTAAAACAGAGGTTCTGTCCACCGTCTCGTAAGTTATGCCCATTCTCCATTAAAA 180
  HvEC_cDNA_004A      TTTGCCTAAAACAGAGGTTCTGTCCACCGTCTC--------------------------- 92
                      *********************************                           

  HvEC_gDNA_004A      AAAAACTTTTAACATATATTTATAACCACTTTCTAGTTCTCTTGACACTCGATCACTTGA 240
  HvEC_cDNA_004A      ------------------------------------TTCTCTTGACACTCGATCACTTGA 116
                                                          ************************

  HvEC_gDNA_004A      TACCGTCATCCAAAGCAACCATCTTTCGAAAAGAGATGAAGGATATACTCTTTATGGCGT 300
  HvEC_cDNA_004A      TACCGTCATCCAAAGCAACCATCTTTCGAAAAGAGATGAAGGATATACTCTTTATGGCGT 176
                      ************************************************************

  HvEC_gDNA_004A      GTGCATTTCATACGTTGCAGAAGGACTTCGCTTGCCTCTCTCGGATAAGTTTGTAAAGTA 360
  HvEC_cDNA_004A      GTGCATTTCATACGTTGCAGAAGGACTTCGCTTGCCTCTCTCGGATAAGTTTGTAAAGTA 236
                      ************************************************************

  HvEC_gDNA_004A      CATCGCAGAAGAGATATCGGATGCTATATCATCCGCTTGGAATTACCCATCCAATACTAT 420
  HvEC_cDNA_004A      CATCGCAGAAGAGATATCGGATGCTATATCATCCGCTTGGAATTACCCATCCAATACTAT 296
                      ************************************************************

  HvEC_gDNA_004A      CTTGCTAAGAAAAAATATCGCCCGATTCGGCTCCAGTAACCTGGACGACGCCCTCGATAA 480
  HvEC_cDNA_004A      CTTGCTAAGAAAAAATATCGCCCGATTCGGCTCCAGTAACCTGGGCGACGCCCTCGATAA 356
                      ******************************************** ***************

  HvEC_gDNA_004A      AGCTATAAAAGATATCTGTAATCAATATCCAGAGAATGATTATTATAGCGTTGAAAATTT 540
  HvEC_cDNA_004A      AGCTATAAAAGATATCTGTAATCAATATCCAGAGAATGATTATTATAGCGTTGAAAATTT 416
                      ************************************************************
```

```
HvEC_gDNA_004A      TGTTATAGATGTTGTGGGGAAGATCTATTCAGCATATGGTCTCGGGAGCCCCAATTTTGC 600
HvEC_cDNA_004A      TGTTATAGAAGTTGTGGGGAAGATCTATTCAGCATATGGTCTCGGGAGCCCCAATTTTGC 476
                    ********* **************************************************

HvEC_gDNA_004A      AGCACGAAAGAATAATCCTAGTGGTCTTTTGGCGTCCAATGCAAGGACGAATGTTAAGAA 660
HvEC_cDNA_004A      AGCACGAAAGAATAATCCTAGTGGTCTTTTGGCGTCCAATGCAAGGACGAATGTTAAGAA 536
                    ************************************************************

HvEC_gDNA_004A      GAAGAAACTTGTAACTTACCCTATTAAAGATCGTCATTTGGGTTAA 706
HvEC_cDNA_004A      GAAGAAACTTGTAACTTACCCTATTAAAGATCGTCATTTGGGTTAA 582
                    **********************************************
```

HvEC-005 alignment

```
CLUSTAL 2.1 multiple sequence alignment

HvEC_gDNA_005      ATGAAAAGAAATCAAGGAATTGTTCCAAAAATTCTCAACTTGG-TAGTTAGCATCCTCGC 59
HvEC_cDNA_005      ATGAAA-GAAATCAGTGTATTTTTCTATA--TTCTCAGCTTGGGTAGT-AGCATCCTCGC 56
                   ****** *******  * *** *** * *  ****** ***** **** ***********

HvEC_gDNA_005      CCCAATCTTGCCTCGATCTCAATTGATCTGGCAACTGGGAAAGCGTGGCGCCCGGATACG 119
HvEC_cDNA_005      CC-AATCTT--CTCGAT-TCAATTTATCTTGCA-CTGG--AAGCGTG-CGCCCGGATACG 108
                   ** ******  ****** ****** **** *** ****  ******* ************

HvEC_gDNA_005      CACAATGAACCGCCCCCCTGTAGGCCATTTTATTCTCTTCATACTTGATAAAGCCTGACA 179
HvEC_cDNA_005      CACAATGAACCACCCCCCT----------------------------------------- 127
                   *********** *******

HvEC_gDNA_005      CTCCTGAGTTACAGCACACGACTGCTGGAGTGAAGAGTATTTCTATGCCTGCGCCGGTGG 239
HvEC_cDNA_005      --------------CACACGACCGCTGGAGTGAAGAGTATTTCTATGCCTGCGCCGGTGG 173
                                 ******** *************************************

HvEC_gDNA_005      TTGCGACACCATTAGTAAGTATTTTATCGCTCACTTTTCATAATTTGTCTCTGAAGTCCT 299
HvEC_cDNA_005      TTGCGACACCATTA---------------------------------------------- 187
                   **************

HvEC_gDNA_005      TGAATTTCTTTTAGCTTCCAGAGATTGTAAACTTGGTGAAAAAACCCGTTCAAGCCAAGT 359
HvEC_cDNA_005      --------------CTTCCAGAGATTGTAAACTTGGTGAAAAAACCCGTTCAAGCCAAGT 233
                                 **********************************************

HvEC_gDNA_005      TTGTGATACGCTAGTTCGTGAATATATAAATACAACCATAGGTATGCGATAATTTTCCAG 419
HvEC_cDNA_005      TTGTGATACGCTAGTTCGTGAATATAAAAATACAACCATAG------------------- 274
                   ************************** **************

HvEC_gDNA_005      AATGGTTTCATTGTATTGATGTTCTATGATTTATTCAGTATGTGGAGTCGGGAATGAACA 479
HvEC_cDNA_005      --------------------------------------TATGTGGAGTCGGGAATGAACA 296
                                                         **********************

HvEC_gDNA_005      ATGGCTTTGTCAATCAGGGCCTACCTCCGTCCGTAAGTGATAACAGTCTATAGATATGCA 539
HvEC_cDNA_005      ATGGCTTTGTCAATCAGGGCCTACCTCCGTCC---------------------------- 328
                   ********************************

HvEC_gDNA_005      GCTGTTTGTGAAAACCTTGCTCTTTTTTTGCTCCTCATCCCCCCCCCCCCAAAAGCGGT 599
HvEC_cDNA_005      ------------------------------------------------------------


HvEC_gDNA_005      TTAAACTCATGTTTTTGGCGGAGAGTGGAAGGCAAAAACATAGCGAGCTTTTCCCAAACA 659
HvEC_cDNA_005      ------------------------------------------------------------


HvEC_gDNA_005      GCTGTATCTATTTAAATTACGTTTCTTACCCTCTAATTTTATTTCCAGAATGCTATGACT 719
HvEC_cDNA_005      ------------------------------------------------AATGCTATGACT 340
                                                                   ************

HvEC_gDNA_005      GCAGAGACGCCATCAAACATTAA 742
HvEC_cDNA_005      GCAGAGACGCCATCAAACATTAA 363
                   ***********************
```

HvEC-006 alignment

```
CLUSTAL 2.1 multiple sequence alignment
```

```
HvEC_gDNA_006      ATGATAAACTTCTTTTGCACCATTTTTGCAACCGTTATGATCGTCCAAATTTCTCTAGCC 60
HvEC_cDNA_006      ATGAAAAACTTCTTTTGCACCATTTTTGCAACCGTTATGATCGTCCAAATTTCTCTAGCC 60
                   **** *******************************************************

HvEC_gDNA_006      GCACCAACTTCTGCCCTGGTGTCTAAACCTTTAATTCATATTTTTAACGGTAGTAAAGTT 120
HvEC_cDNA_006      GTACCAACTTCTGCCCTGGTGTCTAAACCTTTAATTCATATTTTTAACGGTAGTAAAGGT 120
                   * ******************************************************** *

HvEC_gDNA_006      GTTTGGGCTTCATTAACTTCCTTTATTTATATTCCAAAGAAAGAATTATTTGTCGGGTCA 180
HvEC_cDNA_006      GTTTGGGCTTCATTAACTTCCTTTATTTATATTCCAAAGAAAGAATTATTTGTCGGGTCA 180
                   ************************************************************

HvEC_gDNA_006      CACAATCTTCACCTTAACTCGACCAGCGCTGTAAAGGAATTTGCAGATAGTGAGAGAATC 240
HvEC_cDNA_006      CACAATCTTCACCTTAACTCGACCAGCGCTGTAAAGGAATTTGCAGATAGTGAGAGAATC 240
                   ************************************************************

HvEC_gDNA_006      ATGCTTGGAAGAAATGGGTCCAGTGGGGCGAACGCCCTTGAGAAGAATGAAGCCAGAGGC 300
HvEC_cDNA_006      ATGCTTGGAAGAAATGGGGCCAGTGGGGCGAACGCCCTTGAGAAGAATGAAGCCAGAGGC 300
                   ****************** *****************************************

HvEC_gDNA_006      AACATGTCTACGAACTTGAATTTAGAAAAAATTCCTCCCAAAGCGCTTTCTGATGCTCCA 360
HvEC_cDNA_006      AACATGTCTACGAACTTGAATTTAGAAAAAATTCCTCCCAAAGCGCTTTCTGATGCTCCA 360
                   ************************************************************

HvEC_gDNA_006      AAAAAGCGCGCATCCTCTTCCATTGGCGTAGCTTGA 396
HvEC_cDNA_006      AAAAAGCGCGCATCCTCTTCCATTGGCGTAGCTTGA 396
                   ************************************
```

HvEC-007 alignment

CLUSTAL 2.1 multiple sequence alignment

```
HvEC_gDNA_007      ATGCGCTTTTTCTATCTCAGCTACACTTGCGTTCTGGGAGCCGCGCTCATGTCTTTTGGT 60
HvEC_cDNA_007      ATGCGCTTTTTCTATCTCAGCCACACTTGCGTTCTGGGAGCCGCGCTCATGTCTTTTGGT 60
                   ********************* **************************************

HvEC_gDNA_007      TCTCATGCTACCCCTCAGGATTGCATCAAGCCAGGTCCAGATTGTTTTGCGCAAATGGCT 120
HvEC_cDNA_007      TCTCATGCTACCCCTCAGGATTGCATCAAGCCAGGTCCAGATTGTTTTGCGCAAATGGCT 120
                   ************************************************************

HvEC_gDNA_007      AAACGCGATGTAGTTGATCTTCAACATATGCATGCTCATATTCCTCGAGACTTACCCAAC 180
HvEC_cDNA_007      AAACGCGATGTAGTTGATCTTCAACATATGCATGCTCATATTCCTCGAGACTTACCCAAC 180
                   ************************************************************

HvEC_gDNA_007      CCTAATACCCCTGACGTTGCGCGGGTGGAGCACGATCTAGGTAAAAACGGGAACACATTC 240
HvEC_cDNA_007      CCTAATACCCCTGACGTTGCGCGGGTGGAGCACGATCTAGGTAAAAACGGGAACACATTC 240
                   ************************************************************

HvEC_gDNA_007      GGGCATCACCTACAAGCTAGACAAAATGTTCAAGATAAGGATATATACGACGATGAAGCG 300
HvEC_cDNA_007      GGGCATCACCTACAAGCTAGACAAAATGTTCAAGATAAGGATATATACGACGATGAAGCG 300
                   ************************************************************

HvEC_gDNA_007      AACGCTGCAGGCGATGACTAG 321
HvEC_cDNA_007      AACGCTGCAGGCGATGACTAG 321
                   *********************
```

HvEC-008 alignment

CLUSTAL 2.1 multiple sequence alignment

```
HvEC_gDNA_008      ATGAAGTTTTTCCTCCCAATTTTCGTATTTTTATTGCATTGGCTACTGCTATAGAATAT 60
HvEC_cDNA_008      ATGAAGTTTTTCCTCCCAATTTTCGTATTTTTTTTGCATTGGCTACTGCTATAGAATAT 60
                   ******************************** **************************

HvEC_gDNA_008      GACTACAAGCCGCACACTGAGAGAAACTGTAGTCCATTAGCAGGCCAGGTAGAAACCCAG 120
HvEC_cDNA_008      GACTACAAGCCGCACACTGAGAAAAACTGTAGTCCATTAGCAGGCCAGGTAGAAACCCAG 120
                   ********************** *************************************

HvEC_gDNA_008      GAGTTTTTGCATAATGTTACCGGTACCTGTGTGTCAATCAAGAAGTCGGAATATTCAAGT 180
HvEC_cDNA_008      GAGTTTTTGCATAATGTTACCGGTACCTGTGTGTCAATCAAGAAGTCGGAATATTCAAGT 180
                   ************************************************************

HvEC_gDNA_008      TTGAGTGTAAAAAAGCCAACCACTAATCGTTAGTTGATTCCTATTGTTTTTGTTGGCTTT 240
HvEC_cDNA_008      TTGAGTGTAAAAAAGCCAACCACTAATC-------------------------------- 208
                   ****************************

HvEC_gDNA_008      CTACTGCAGAGTATAAACAGCTCTAACAATAACAATCATTTTCTTCGATGGAAATTTAGT 300
```

```
  HvEC_cDNA_008      -----------------------------------------------------------T 209
                                                                                *

  HvEC_gDNA_008      TGCTATCTATCTATACTGTGACCCTCACTGCTCAAACAAATCACACACTTTTATGATTGA 360
  HvEC_cDNA_008      TGCTATCTATCTATACTGTGACCCTCACTGCTCAAACAAATCACACACTTTTATGATTGA 269
                     ************************************************************

  HvEC_gDNA_008      CAAGGCTCCAACAAAGTGTATTGGCCTAACGGATTAGTAAGTGCTCTGACTTCCAAAGAT 420
  HvEC_cDNA_008      CAAGGCTCCAACAAAGTGTATTGGCCTAACGGATTA------------------------ 305
                     ************************************

  HvEC_gDNA_008      TCTTTTTTTCACAGGCGTCATAATGATTTTAGAGAAGGGAGGGGGAAGACTAGCTAGGCA 480
  HvEC_cDNA_008      ------------------------------------------------------------


  HvEC_gDNA_008      GTTTGTTGACAATCAGAGATTGACTTCTGAGAAAAGGCAGTTCAAAGGCAATCTTACAGC 540
  HvEC_cDNA_008      ------------------------------------------------------------


  HvEC_gDNA_008      TAATACGGGAATGCTTGCGGTTGTCTTGCCTATTATCTCCCGCTAAAAGCATAGCTCGTC 600
  HvEC_cDNA_008      ------------------------------------------------------------


  HvEC_gDNA_008      CGAACTCAACTTATGATTTTGGCGGGAAGATGGGATGCGAGGCAATCGCAAGCTTTCCAG 660
  HvEC_cDNA_008      ------------------------------------------------------------


  HvEC_gDNA_008      GATTAGTAGTATATACTTTCGCCTTGGTTTGAAAACCCAACTCGGGCTCAACAAGGAAAC 720
  HvEC_cDNA_008      ------------------------------------------------------------


  HvEC_gDNA_008      TCGCTAGCTAATCCCCCTCCCCTTACCCACGCAGATACAATTTCACGTAGCGATGATTAA 780
  HvEC_cDNA_008      ------------------------------------------------------------


  HvEC_gDNA_008      CTAACTGATTTTCGTAAATCTATTTAATGGAAACCGATTTTCATGATTTCAAATACGCTT 840
  HvEC_cDNA_008      ------------------------------------------------------------


  HvEC_gDNA_008      TTTAGTTGTCCAACCCAATCATTTGAAGTGGTTAACGCCAACTAA 885
  HvEC_cDNA_008      -----TTGTCCAACCCAATCATTTGAAGTGGTTAACGCCAACTAA 345
                          ****************************************
```

HvEC-016 alignment

```
CLUSTAL 2.1 multiple sequence alignment

HVEC_cDNA_016      ATGTTGGCCTCTACATTCTTCTTCATTTTCTTTGCTCTATGTAGT--------------- 45
HVEC_gDNA_016      ATGTTGGCCTCTACATTCTTCTTCATTTTCTTTGCTCTATGTAGTAAGGTCATTTTGATG 60
                   *********************************************

HVEC_cDNA_016      ----------------------------------------------TGGAACTCCATTAA 59
HVEC_gDNA_016      ATGATATGAAGAAGCCTATTGCTAATGTAATATTTCCTTTTCAGGTTGGAACTCCATTAA 120
                                                                 **************

HVEC_cDNA_016      TTTTGTAGCG-------------------------------------------------- 69
HVEC_gDNA_016      TTTTGTAGCGTAGGTCGATCAGCTATTGCCTTTTTGCTAATAAATGGCTAACTTTTGTTC 180
                   **********

HVEC_cDNA_016      --------------GCCCAAGACGCAACGGTGATCGCTCAAGAATACATTCAGGTCACTA 115
HVEC_gDNA_016      ATGTATTTTCTAGGGCCCAAGACGCAACGGTGATCGCTCAAGAATACATTCAGGTCACTA 240
                                 **********************************************

HVEC_cDNA_016      ATAGCATTCAGACTCAGATCTCGACATGCAAGCAACTCGTCGCCAGTAGCAGTGGGCGGC 175
HVEC_gDNA_016      ATAGCATTCAGACTCAGATCTCGACATGCAAGCAACTCGTCGCCAGTAGCAGTGGGCGGC 300
                   ************************************************************

HVEC_cDNA_016      CACAAGTCATCACCCAATTTTACCAGGTCATTCAATCGTTCCAAATAAAAATAAGGAGTA 235
HVEC_gDNA_016      CACAAGTCATCACCCAATTTTACCAGGTCATTCAATCGTTCCAAATAAAAATAAGGAGTA 360
                   ************************************************************

HVEC_cDNA_016      AATGTAAAGGTTGTGGTAGTGGAATCACTGCAGCGTGCCACC------------------ 277
HVEC_gDNA_016      AATGTAAAGGTTGTGGTAGTGGAATCACTGCAGCGTGCCACCAGGCGAGTTTTTTTTTTA 420
                   ******************************************

HVEC_cDNA_016      ------------------------------------------------------------
HVEC_gDNA_016      TTTATTTATTTATTTTCTTTTTATTTTTATTTTTATTATTATTTTTATTTTTTGGAAACT 480
```

```
HVEC_cDNA_016      ---------------------------AGAACCGAGTTGATGTTCGAATAAAGGAAGTTA 310
HVEC_gDNA_016      CAATGGCTAATTCAACCTCATGTTTCCAGAACCGAGTTGATGTTCGAATAAAGGAAGTTA 540
                                              *********************************

HVEC_cDNA_016      TTGATACATTCCAATCCCTCGTTATAACTATGTATGACACCCCAGGACTTCTTCAAGCTT 370
HVEC_gDNA_016      TTGATACATTCCAATCCCTCGTTATAACTATGTATGACACCCCAGGACTTCTTCAAGCTT 600
                   ************************************************************

HVEC_cDNA_016      GTATCAGT---------------------------------------------------- 378
HVEC_gDNA_016      GTATCAGTGCGTCTTTCACACTTCACTTTCGGTAGTTTGGCGAAATATTAACAACTTTCC 660
                   ********

HVEC_cDNA_016      -----------------------------------------TACGTCACAGTTGAGAAAA 397
HVEC_gDNA_016      ACCCTGATTCTATTCTGCTCCTCTTTGAACCTTGTGCAGGTTACGTCACAGTTGAGAAAA 720
                                                            *******************

HVEC_cDNA_016      TAACGGCTTCATACACTTTATTGATGAAAAATTGTCGGATTATTACTGGCTACCAAGTTA 457
HVEC_gDNA_016      TAACGGCTTCATACACTTTATTGATGAAAAATTGTCGGATTATTACTGGCTACCAAGTTA 780
                   ************************************************************

HVEC_cDNA_016      CACAATATGTTACGCAACTATTTATCACTACTATGCAAAATTGTGGAGTGTCTATTAGTG 517
HVEC_gDNA_016      CACAATATGTTACGCAACTATTTATCACTACTATGCAAAATTGTGGAGTGTCTATTAGTG 840
                   ************************************************************

HVEC_cDNA_016      CAGTGATAAGTGGAGTTGATAGCATCAGCATTAATACGATTATTGGTGGTTTAGGGATTG 577
HVEC_gDNA_016      CAGTGTTAAGTGGAGTTGATAGCATCAGCATTAATACGATTATTGGTGGTTTAGGGATTG 900
                   ***** ******************************************************

HVEC_cDNA_016      ATTTGAATATTGGCTTTGGGATTAATATTGGTGGAAGTGGAGGCGGGGGCGGGGGTGGTA 637
HVEC_GDNA_016      ATTTGAATATTGGCTTTGGGATTAATATTGGTGGAAGTGGAGGCGGGGGCGGGGGTGGTA 960
                   ************************************************************

HVEC_cDNA_016      TTTCTGTTGGAGGCAATATTGGTATTGGTATAAATATTTAG 678
HVEC_gDNA_016      TTTCTGTTGGAGGCAATATTGGTATTGGTATAAATATTTAG 1000
                   *****************************************
```

HvEC-020 alignment

```
CLUSTAL 2.1 multiple sequence alignment


  HvEC_gDNA_020      ATGTACACCAAAAGTCTCTTGGCTCTCAGTGCAGCCGCCTTTAGTAGGCTTCTAATCTCA 60
  HvEC_cDNA_020      ATGTACACCAAAAGTCTCTTGGCTCTCAGTGCAGCCGCCTTTA----------------- 43
                     *******************************************

  HvEC_gDNA_020      TTAACTGTTGGCCGGTTAGCACTAACTGAATAGCATTTTATTTCATTTAGCATTGTTGGG 120
  HvEC_cDNA_020      --------------------------------------------------CATTGTTGGG 53
                                                                       **********

  HvEC_gDNA_020      GTCAGTTCCAGCAGCCGCTGCACCGGCCGAGGAAACTGGCGCCCAGTATGGAGGCGACAT 180
  HvEC_cDNA_020      GTCAGTTCCAGCAGCCGCTGCACCGGCCGAGGAAACTGGCGCCCAGTATGGAGGCGACAT 113
                     ************************************************************

  HvEC_gDNA_020      CTCTGGAGGGTGAGTGCATACTGATTGATTGATGAGTCTTACCTGTAATAGGAGCTGACT 240
  HvEC_cDNA_020      CTCTGGAGG--------------------------------------------------- 122
                     *********

  HvEC_gDNA_020      GTTACAATGGGGCGGGATGACGCTATTGGCGGTGGTTTTGTCGGTAGCTCTNCAAATTGG 300
  HvEC_cDNA_020      -----------------------------------------------CTCTC-AAATTGG 134
                                                                    ****  *******

  HvEC_gDNA_020      CGGAGGGGCACTATGGTGGCGGTGGAGGTTACGGCGGTGGAGTCAACGGAGGTATCGGAG 360
  HvEC_cDNA_020      CGGAGGG-CACTATGGTGGCGGTGGAGGTTACGGCGGGGGAGTCAACGGAGGTATCGGAG 193
                     ******* ***************************** **********************

  HvEC_gDNA_020      GCAGCGTCAGCGGTGGAGTTACCTTCACCAGTGCTCTTTCCCCATTAAGAACGAACATTG 420
  HvEC_cDNA_020      GCAGCGTCAGCGGTGGAGTTACCTTCACCAGTGCTCTTTCCCCATTAAGAACGAACATTG 253
                     ************************************************************

  HvEC_gDNA_020      AGCAAGTGGAAAGCAGAGTTACAAGCGTGAGATCCTATGATCGCAGCTCTTCAGTGACTA 480
  HvEC_cDNA_020      AGCAAGTGGAAAGCAGAGTTACAAGCGTGAGATCCTATGATCGCAGCTCTTCAGTGACTA 313
                     ************************************************************

  HvEC_gDNA_020      TCATAAAATCTCTCCACTCTTTCTACGAATACTCTCAGAGAGTTGCTATATACCTGAGAT 540
  HvEC_cDNA_020      TCATAAAATCTCTCCACTCTTTCTACGAATACTCTCAGAGAGTTGCTATATACCTGAGAT 373
                     ************************************************************

  HvEC_gDNA_020      CCAATTACCGTAGTAGTAGGTCCATTGATGTAGTAAGTCTCCTTTATTTATACAACGGAG 600
```

```
HvEC_cDNA_020      CCAATTACCGTAGTAGTAGGTCCATTGATGTA---------------------------- 405
                   ********************************

HvEC_gDNA_020      AGCTCCTAAGTGGTGAACCAGTAAGATACCTCTTTTGTAGTCTTCAGTTGTTAATGTCTG 660
HvEC_cDNA_020      ----------------------------------------TCTTCAGTTGTTAATGTCTG 425
                                                           ********************

HvEC_gDNA_020      GAGCACCGTCTTGATTAAGTATCAAGCAATCATCAAACACGTCCGCCAAAGTCAATACTG 720
HvEC_cDNA_020      GAGCACCGTCTTGATTAAGTATCAAGCAATCATCAAACACGTCCGCCAAAGTCAATACTG 485
                   ************************************************************

HvEC_gDNA_020      GGGTGAATGCCGCGGTAGGTATTTCAATTGAAACTTGAATTTTTCAAATTAAATCTTGTT 780
HvEC_cDNA_020      GGGTGAATGCCGCG---------------------------------------------- 499
                   **************

HvEC_gDNA_020      AATTCACTCCAAACTTCGACTATTCGCATATTGTAGAGGAGGTTGAAAAAGTATCAGTTT 840
HvEC_cDNA_020      ------------------------------------AGGAGGTTGAAAAAGTATCAGTTT 523
                                                       ************************

HvEC_gDNA_020      CCATTGAAGAGATTTTCAAAATTACTACCCAAACCACCAGAAGAATCGGGATTTACGACA 900
HvEC_cDNA_020      CCATTGAAGAGATTTTCAAAATTACTACCCAAACCACCAGAAGAATCGGGATTTACGACA 583
                   ************************************************************

HvEC_gDNA_020      ACTTCAGACAACAACTCAGGGGTCGTGTCTCAGGCAGATTGGACTATCGATTGCTTTCGG 960
HvEC_cDNA_020      ACTTCAGACAACAACTCAGGGGTCGTGTCTCAGGCAGATTGGACTACCGATTGCTTTCGG 643
                   ********************************************** *************

HvEC_gDNA_020      ATGCGCGATGCTCCGTCAGACAATACATTGAAGGGTAA 998
HvEC_cDNA_020      ATGCGCGATGCTCCGTCAGACAATACATTGAAGGGTAA 681
                   **************************************
```

HvEC-021 alignment

```
CLUSTAL 2.1 multiple sequence alignment

HvEC_gDNA_021      ATGAAAATCAATATGTGGTGTATGAATCTTTTCCTTGTTGCTTTTGTCGGGGTGCGATCA 60
HvEC_cDNA_021      ATGAAAATCAATATGTGGTGTATGAATCTTTTCCTTGTTGCTTTTGTCGGGGTGCGATCA 60
                   ************************************************************

HvEC_gDNA_021      TCACCTCAAAACTTCGTAAAAAGGAGCCCCGAAAACGTTGCGTGCTCTACATTTTGGTCT 120
HvEC_cDNA_021      TCACCTCAAAACTTCGTAAAAAGGAGCCCCGAAAACGTTGCGTGCTCTACATTTTGGTCT 120
                   ************************************************************

HvEC_gDNA_021      CACGGGCAGATAACTGATGGAACCAATAGTGAAGTGGTGAGCTCAGTGAGACTTATAATG 180
HvEC_cDNA_021      CACGGGCAGATAACTGATGGAACCAATAGTGGA-----------AGTG------------ 157
                   ******************************* *           ****

HvEC_gDNA_021      ACTGGGTAACAATTACAGTTTTCACACATTGTGCTATTATTTTATAGGTGCTTTTTGCTC 240
HvEC_cDNA_021      -----------------------------------------------GTGCTTTTTGCTC 170
                                                                  *************

HvEC_gDNA_021      TGCAGGAGTAGATGCCAGCCTCTATTACTGCGACCTCAAACAATGTGAAGGCTCTATTTG 300
HvEC_cDNA_021      TGCAGGAGTAGATGCCAGCCTCTATTACTGCGACCTCAAACAATGTGAAGGCTCTATTT- 229
                   ***********************************************************

HvEC_gDNA_021      TATGTCTAAATCAGGTTGCTTAGCACCTGTGATTCTCAAGTTACACTTTATAACTGTCTT 360
HvEC_cDNA_021      ------------------------------------------------------------

HvEC_gDNA_021      TTAAAAAATATAGCTTGTAAAGGTTGTCTTAGTGGCGGGAGTCAAGCCAATACAACAGTC 420
HvEC_cDNA_021      -------------CTTGTAAAGGTTGTCTTAGTGGCGGGAGTCAAGCCAATACAACAGTC 276
                                *******************************************************

HvEC_gDNA_021      CAGGACATGGAATGCGTGGAATCATATATTCAACAAACTGCTGGTCTTGCAAGTAAGTAA 480
HvEC_cDNA_021      CAGGACATGGAATGCGTGGAATCATATATTCAACAAACTGCTGGTCTTGCAAG------- 329
                   *****************************************************

HvEC_gDNA_021      CTTAGTCGAGTCTTCTATATATTTAATTCTAATTTCACCTTTGATCCAATCTCTCTCAAG 540
HvEC_cDNA_021      ------------------------------------------------------------

HvEC_gDNA_021      GCTGTATTGACGACAACCATATTGTTGGAATTTGCCAACCAAAAGAATGTGTTAACGCTA 600
HvEC_cDNA_021      -CTGTATTGACGACAACCATATTGTTGGAATTTGCCAACCAAAAGAATGTGTTAACGCTA 388
                    ***********************************************************

HvEC_gDNA_021      AAAGTAGGATCCGATTTTGAGTTTATCTTCTTTTTTAGCCCATCTAACTATGCTTTTCTC 660
HvEC_cDNA_021      AAA--------------------------------------------------------- 391
                   ***
```

```
  HvEC_gDNA_021      TAGTGTGTCGCTCTTGCGTAAAATTGGACAGTGAATTCAACTCTAACTCGATTGATGAGA 720
  HvEC_cDNA_021      ---TGTGTCGCTCTTGCGTAAAATTGGACAGTGAATTCAACTCTAACTCAATTGATGAGA 448
                        ********************************************** **********

  HvEC_gDNA_021      AAGACAACAATCCGGCCCTTTCTCAAACTGCGAAACTAACTCGTCGTGGAAATGGTTTCG 780
  HvEC_cDNA_021      AAGACAACAATCCGGCCCTTTCTCAAACTGCGAAACTAACTCGTCGTGGAAATGGTTTCG 508
                     ************************************************************

  HvEC_gDNA_021      CAGCAGGAATGGGTGGTGAAGCGCAAGCTGGTTCTTCCGAGCAATTTTTCGGAGGAAGTT 840
  HvEC_cDNA_021      CAGCAGGAATGGGTGGTGAAGCGCAAGCTGGTTCTTCCGAGCAATTTTTCGGAGGAAGTT 568
                     ************************************************************

  HvEC_gDNA_021      TAGTTCTCCGTGCCTAA 857
  HvEC_cDNA_021      TAGTTCTCCGTGCCTAA 585
                     *****************
```

HvEC-022 alignment

```
CLUSTAL 2.1 multiple sequence alignment


  HvEC_gDNA_022      ATGTTGGCCTTTAACTTCGTAGCCGTTGCTCTTTTGGGTAAGTGATCAAGTCTTAAATGG 60
  HvEC_cDNA_022      ATGTTGGCCTTTAACTTCGTAGCCGTTGCTCTTTTGG----------------------- 37
                     *************************************

  HvEC_gDNA_022      ATTATCCTGTGTGCATTCCATTAATGTTTGCTATTCCAGCATTCTCTAGCGCTGCCCTAG 120
  HvEC_cDNA_022      ---------------------------------------CATTCTCTAGCGTTGCCCTAG 58
                                                            ************ ********

  HvEC_gDNA_022      CTTCGTAAGTATCGTTAGACTTTGTTCAACCATCATCAAACTGAATATATTATATGCAGA 180
  HvEC_cDNA_022      CTTCC------------------------------------------------------- 63
                     ****

  HvEC_gDNA_022      CCCGAGCCATGCCCTACATGCCCAGTCACACCACCCCCAGACTGTGGCGGTTCTTGCCCA 240
  HvEC_cDNA_022      CCCGAGCCATGCCCTACATGCCCAGTCACACCACCCCCAGACTGTGGCGGTTCTTGCCCA 123
                     ************************************************************

  HvEC_gDNA_022      TCCCCGTGCAATCCTTGTGCACAACAGATCCAAACTGCTTGCAACACATGTGGTGGATTT 300
  HvEC_cDNA_022      TCCCCGTGCAATCCTTGTGCACAACAGATCCAACCTGCTTGCAACACATGTGGTGGATTT 183
                     ********************************* **************************

  HvEC_gDNA_022      GATAACGGCGGTGGTTCTCAATTCGGTAATTACGGTGGTGGTCCTCCTGGCAATAATCTC 360
  HvEC_cDNA_022      GATAACGGCGGTGGTTCTCAATTCGGTAATTACGGTGGTGGTCCTCCTGGCAATAATCTC 243
                     ************************************************************

  HvEC_gDNA_022      CCGTTCGGTGGTCCAGGGGGTTTAGGTGGTCTAGGTGGTCCAGGTGGATTGCCTGGTGGC 420
  HvEC_cDNA_022      CCGTTCGGTGGTCCAGGGGGTTTAGGTGGTCTAGGTGGTCCAGGTGGATTGCCTGGTGGC 303
                     ************************************************************

  HvEC_gDNA_022      GGTGGTCTTGGCGGTGGTCTTGGCGGTGGTCTTTTATCAGGCCTGCTCAAAAGATCAAAA 480
  HvEC_cDNA_022      GGTGGTCTTGGCGGTGGTCTTGGCGGTGGTCTTTTATCAGGCCTGCTCAAAAGATCAAAA 363
                     ************************************************************

  HvEC_gDNA_022      TCGGAGGCTAAATAG 495
  HvEC_cDNA_022      TCGGAGGCTAAATAG 378
                     ***************
```

HvEC-024 alignment

```
CLUSTAL 2.1 multiple sequence alignment


HvEC_cDNA_024      ATGGCTTTGGTCGTATTGGCCGTATCGTTGCTCGTAATGCCATCATTCACGGAGGCGTTG 60
HvEC_gDNA_024      ATGGCTTTGGTCGTATTGGCCGTATCGTTGCTCGTAATGCCATCATTCACGGAGGCGTTG 60
                   ************************************************************

HvEC_cDNA_024      ACATTGTCGC-------------------------------------------------- 70
HvEC_gDNA_024      ACATTGTCGCGTAAGCTACTGCTTTGATTTGCGTCCTGTAGCTCCCATTTCAAATATCTG 120
                   **********

HvEC_cDNA_024      ------------------AATCAATGACCCCTTTATTGATCTTGACTACATGGT------ 106
HvEC_gDNA_024      ACAATTTTAACAACACAGAATCAATGACCCCTTTATTGATCTTGACTACATGGTAGGACT 180
                                     ************************************

HvEC_cDNA_024      ------------------------------------------------------------
HvEC_gDNA_024      TATGATTCTTTACATTTCCATTCAACATATTGTTGACATGCTTATCTTTTTTCCCTTTCT 240
```

```
HvEC_cDNA_024      ----TTACATGTTCAAGTTCGACTCCACTCACGGCAAATTCAAGGGTACCGTCACCGCTG 0162
HvEC_gDNA_024      AGGTTTACATGTTCAAGTTCGACTCCACTCACGGCAAATTCAAGGGTACCGTCACCGCTG 300
                       ********************************************************

HvEC_cDNA_024      AAGGCGGTAAACTCGTCATTGATGGAAAACCTATCAATGTCTTTGCCGAACGGGATCCTG 222
HvEC_gDNA_024      AAGGCGGTAAACTCGTCATTGATGGAAAACCTATCAATGTCTTTGCCGAACGGGATCCTG 360
                   ************************************************************

HvEC_cDNA_024      CTAACATCCCCTGGGGAAAAGTAG 246
HvEC_gDNA_024      CTAACATCCCCTGGGGAAAAGTAG 384
                   ************************
```

HvEC-025 alignment

```
CLUSTAL 2.1 multiple sequence alignment


HvEC_cDNA_025      ATGATTTTTTCAATGGTTTCCTTCTTGTTTTTGACTG----------------------- 37
HvEC_gDNA_025      ATGATTTTTTCAATGGTTTCCTTCTTGTTTTTGACTGGTAAATAGATGATTTCGATAATT 60
                   *************************************

HvEC_cDNA_025      ------------------------------------------TTTTCATTTCAAACCTCT 55
HvEC_gDNA_025      CAATTAGATATCAACACTTTAACCACTTACTTCTCATTACAGTTTTCATTTCAAACCTCT 120
                                                             ******************

HvEC_cDNA_025      CTGCCGCTCCAATCATTGAAGAAACGGATCCCTCCTCCAA-------------------- 95
HvEC_gDNA_025      CTGCCGCTCCAATCATTGAAGAAACGGATCCCTCCTCCAAGTCAATTACACTTTTCCTTT 180
                   ****************************************

HvEC_cDNA_025      ----------------------------------------------------CTCTCTTG 103
HvEC_gDNA_025      TTCATGATCCCTTTGTTCATTGATTTTATCCTTACTTTATGCTTCTTTCTAGCTCTCTTG 240
                                                                       ********

HvEC_cDNA_025      ATCGTCGAACGTTTCTTTATGGATATCCTGGTTACGGTTATTATTGGATATCCTGGTTAC 0162
HvEC_gDNA_025      ATCGTCGAACGTTTCTTTATGGATATCCTGGTTACGGTTATTATTGGATATCCTGGTTAC 300
                   ************************************************************

HvEC_cDNA_025      GGCTATAATGATAATTATTATGGTGCTGGAGTAGCAGCCGGTGCTGGGGTGGCCGTACTT 222
HvEC_gDNA_025      GGCTATAATGATAATTATTATGGTGCTGGAGTAGCAGCCGGTGCTGGGGTGGCCGTACTT 360
                   ************************************************************

HvEC_cDNA_025      TAA 225
HvEC_gDNA_025      TAA 363
                   ***
```

HvEC-026 alignment

```
CLUSTAL 2.1 multiple sequence alignment


HvEC_cDNA_026      ATGAATTTTTCAATTGTTTCCGTCTTGTTTTTGAGTG----------------------- 37
HvEC_gDNA_026      ATGAATTTTTCAATTGTTTCCGTCTTGTTTTTGAGTGGTAAATAGATGATTTCATTACTT 60
                   *************************************

HvEC_cDNA_026      ------------------------------------------TTTTCATTTCAAGCCTCT 55
HvEC_gDNA_026      CAATTCGATATCAACATTTTAACCTTATGCGTCCCATTCCAGTTTTCATTTCAAGCCTCT 120
                                                             ******************

HvEC_cDNA_026      CCGCCTCTCCAATCATCAAAGAAACTGATCCCTCCTCCAA-------------------- 95
HvEC_gDNA_026      CCGCCTCTCCAATCATCAAAGAAACTGATCCCTCCTCCAAGTCAGTTACACTTTTCTTTT 180
                   ****************************************

HvEC_cDNA_026      --------------------------------------------------CTCTCTTGAA 105
HvEC_gDNA_026      TTATGATCCGGTTGTTCATTTATTTTATCCTTATATTATCTTCTTTCTAGCTCTCTTGAA 240
                                                                     **********

HvEC_cDNA_026      CGTCGAACGTTTCTTTATGGATATCCTGGTTACGGTTATTATGGATATCCTGGTTACGGC 0165
HvEC_gDNA_026      CGTCGAACGTTTCTTTATGGATATCCTGGTTACGGTTATTATGGATATCCTGGTTACGGC 300
                   ************************************************************

HvEC_cDNA_026      TATAATGATAATTATTATGGTGCTGGAGTAGCAGCCGGTGCTGGGGTGGCCGTACTTTAA 225
HvEC_gDNA_026      TATAATGATAATTATTATGGTGCTGGAGTAGCAGCCGGTGCTGGGGTGGCCGTACTTTAA 360
                   ************************************************************
```

HvEC-029 alignment

```
CLUSTAL 2.1 multiple sequence alignment


HvEC_cDNA_029      ATGAGATCTTTCCTGGTATTTTTCTCCCTCGTGGTAGCCATTAGTGCAAAAGATTTTGTT 60
HvEC_gDNA_029      ATGAGATCTTTCCTGGTATTTTTCTCCCTCGTGGTAGCCATTAGTGCAAAAGATTTTGTT 60
                   ************************************************************

HvEC_cDNA_029      TACCAATTCTATAAAAACCAAAATTGTACGCTTCCTCGAATTTTTGGTCATGGACCAGTT 120
HvEC_gDNA_029      TACCAATTCTATAAAAACCAAAATTGTACGCTTCCTCGAATTTTTGGTCATGGACCAGTT 120
                   ************************************************************

HvEC_cDNA_029      ATTACCACCGAGGGCTCTTTTGAAGAATGTATTACCACAAAGGAGAAGAATGTTGAATTT 180
HvEC_gDNA_029      ATTACCACCGAGGGCTCTTTTGAAGAATGTATTACCACAAAGGAGAAGAATGTTGAATTT 180
                   ************************************************************

HvEC_cDNA_029      CATAGCATTTTGGTTGCTCCTCACGCACAGA----------------------------- 211
HvEC_gDNA_029      CATAGCATTTTGGTTGCTCCTCACGCACAGAGTATGTAACATTCGCCCGTTTCTAACTTG 240
                   *******************************

HvEC_cDNA_029      --------------------------------------------ACATTGAGGCTAATGT 227
HvEC_gDNA_029      AAAGTCCATGCTAACATTTGGTGTCACAACCATTCTTATCTCAGACATTGAGGCTAATGT 300
                                                               ****************

HvEC_cDNA_029      TGCAATTTACCCTCTACCCGGTTGCAAGGGCGCGAAATACATAACAAGACTTCCTCATGA 287
HvEC_gDNA_029      TGCAATTTACCCTCTACCCGGTTGCAAGGGCGCGAAATACATAACAAGACTTCCTCATGA 360
                   ************************************************************

HvEC_cDNA_029      CCACTGTGTATTGCCCGCAAAGGG------------------------------------ 311
HvEC_gDNA_029      CCACTGTGTATTGCCCGCAAAGGGGTAAGTGAATTTAATGATTACCCGTTTGATAAAATG 420
                   ************************

HvEC_cDNA_029      ----------------------------------------------------------CG 313
HvEC_gDNA_029      AATGGGAATCCCCGTTTTCTAATCCTACACTAATTCTTGTCCTCAATTCTAATTATAGCG 480
                                                                             **

HvEC_cDNA_029      AGATTGCCACTTCTTTTCGAGTCCTTCCTATTCGACATTGA 354
HvEC_gDNA_029      AGATTGCCACTTCTTTTCGAGTCCTTCCTATTCGACATTGA 521
                   *****************************************
```

HvEC-030 alignment

```
CLUSTAL 2.1 multiple sequence alignment


HvEC_cDNA_030      ATGAAATTCTCACTTCAAGTGATAGGTTTATTGGCAATTGTCGCTAGTGTTGCACTTGCT 60
HVEC_gDNA_030      ATGAAATTCTCACTTCAAGTGATAGGTTTATTGGCAATTGTCGCTAGTGTTGCACTTGCT 60
                   ************************************************************

HvEC_cDNA_030      GAGGATTTTAGTTGGAAGTCTTATTCGACAAAAGATTGCAGACCGCCAGCTGGTACTTTC 120
HVEC_gDNA_030      GAGGATTTTAGTTGGAAGTCTTATTCGACAAAAGATTGCAGACCGCCAGCTGGTACTTTC 120
                   ************************************************************

HvEC_cDNA_030      GAAACTGAAATCATCTTCACTGGAAGTTTCGACAAATGCATCAGCATATCAGCGAAAAAA 180
HVEC_gDNA_030      GAAACTGAAATCATCTTCACTGGAAGTTTCGACAAATGCATCAGCATATCAGCGAAAAAA 180
                   ************************************************************

HvEC_cDNA_030      CCGGCAGGAAGTATTAGATTTACACCAATGAAAAGCGGACCTT----------------- 223
HVEC_gDNA_030      CCGGCAGGAAGTATTAGATTTACACCAATGAAAAGCGGACCTTGTAAGTAAAAAACTTGG 240
                   *******************************************

HvEC_cDNA_030      ------------------------------------------------------------
HVEC_gDNA_030      ATTATTGATTTTTGGCACTTGATTCATCTTTGTTGTACATGTATTCAATGACATTTCTCT 300


HvEC_cDNA_030      ----------------------ATAAAACTCAGGTTTATTGCTCTGATAAATGTGGTGGC 261
HVEC_gDNA_030      CTTCCAAATGTTCAATTGGCAGATAAAACTCAGGTTTATTGCTCTGATAAATGTGGTGGC 360
                                         **************************************

HvEC_cDNA_030      GCGGTCAAGACTATAAATCACCCAGCGAAAAATGGTACTCAGTGTTTGTTGCCTTTGGGT 321
HVEC_gDNA_030      GCGGTCAAGACTATAAATCACCCAGCGAAAAATGGTACTCAGTGTTTGTTGCCTTTGGGT 420
                   ************************************************************

HvEC_cDNA_030      CCTCCACT---------------------------------------------------- 329
HVEC_gDNA_030      CCTCCACTGTAAGAATATCTCTTATTTCTTTTCAATGATATGAAAACCTATCTCTAAAAC 480
                   ********
```

```
HvEC_cDNA_030      -----------------------------------------------AAAAATCTATTGT 342
HVEC_gDNA_030      GTATTAATCTGAAATATTTTTATCTTTTCATTGCATTTTCTCTTTAGAAAAATCTATTGT 540
                                                                 *************

HvEC_cDNA_030      CCTGGTGCAGCATCCTTCCGAGCCAGCCCAATTTATGTTTGA 384
HVEC_gDNA_030      CCTGGTGCAGCATCCTTCCGAGCCAGCCCAATTTATGTTTGA 582
                   ******************************************
```

HvEC-031 alignment

```
CLUSTAL 2.1 multiple sequence alignment


HvEC_cDNA_031      ATGAAGTTATTCTATTACACTCTTTTATCGCTCGTGTTGGCCTTCTCCTTAATTCATAAC 60
HvEC_gDNA_031      ATGAAGTTATTCTATTACACTCTTTTATCGCTCGTGTTGGCCTTCTCCTTAATTCATAAC 60
                   ************************************************************

HvEC_cDNA_031      ACGAAGGCTGATACCGTAGTTTCATTGCTTCCTAAATTCAAAACTTGTGCCCAGGAAGCT 120
HvEC_gDNA_031      ACGAAGGCTGATACCGTAGTTTCATTGCTTCCTAAATTCAAAACTTGTGCCCAGGAAGCT 120
                   ************************************************************

HvEC_cDNA_031      TTTTTCAGGGCATATGTTGACGCTGGAGCAAATTGTGGT--------------------- 159
HvEC_gDNA_031      TTTTTCAGGGCATATGTTGACGCTGGAGCAAATTGTGGTGTAAGTAAATGTGTGGAGAAA 180
                   ***************************************

HvEC_cDNA_031      ------------------------------------------ATTGATTTGAAGTGCCTT 177
HvEC_gDNA_031      AGATATTTATGAGCGGTTTCTGACACAAATCTTTCGCTACAGATTGATTTGAAGTGCCTT 240
                                                             ******************

HvEC_cDNA_031      TGTCAAAGCCAGGCTTTCATTTGCGCAAGTATGAACCGAATCACCTCCAAATGTCCATCA 237
HvEC_gDNA_031      TGTCAAAGCCAGGCTTTCATTTGCGCAAGTATGAACCGAATCACCTCCAAATGTCCATCA 300
                   ************************************************************

HvEC_cDNA_031      TTTTTTGACCAACTTGGAGCATTGAGTACCTGTACTCAAG-------------------- 277
HvEC_gDNA_031      TTTTTTGACCAACTTGGAGCATTGAGTACCTGTACTCAAGTAAGTCATCCTCTCATAAAT 360
                   ****************************************

HvEC_cDNA_031      ----------------------------------------------------GATGTCGA 285
HvEC_gDNA_031      TCTAACTCTTCAATAATCACGGTACGTTAATCATATACTACTGTTAATTAGGGATGTCGA 420
                                                                       ********

HvEC_cDNA_031      TCTGTTGGAGTTTCTAGCGTCAATGTAAACTCATGCTATTGA 327
HvEC_gDNA_031      TCTGTTGGAGTTTCTAGCGTCAATGTAAACTAGTGCTATTGA 458
                   ******************************************
```

HvEC-032 alignment

```
CLUSTAL 2.1 multiple sequence alignment


HvEC_cDNA_032      ATGTCGGTCCTCTTCCCCACAATCCTCATCCTCCTCATCCTCCTAAAAAACTCAAAC--- 57
HvEC_gDNA_032      ATGTCGGTCCTCTTCCCCACAATCCTCATCCTCCTCATCCTCCTAAAAAACTCAAACGTC 60
                   *********************************************************

HvEC_cDNA_032      ------------------------------------------------------------
HvEC_gDNA_032      GGCCTCTTCTCTATCTTTCAATACTCAACCATCTACCAACTAACTTATCCCTCTTCTTCT 120


HvEC_cDNA_032      ----CCATCCCTCATCTCAATTAATATCTCAATCAAATCCCTTCCGCTCATCGCTACTAC 113
HvEC_gDNA_032      TCAGTCATCCCTCATCTCAATTAATATCTCAATCAAATCCCTTCCGCTCATCGCTACTAC 180
                       *******************************************************

HvEC_cDNA_032      AGATCCTTCATGCATCGTGATCTCCTTTCTTGGCTCTCCGTCACTTG------------- 0160
HvEC_gDNA_032      AGATCCTTCATGCATCGTGATCTCCTTTCTTGGCTCTCCGTCACTTGGTCCTATCCTCTT 240
                   ***********************************************

HvEC_cDNA_032      ------------------------------------------------------------
HvEC_gDNA_032      CTTCTTCTTCTATCTTCTTCTACTTACTCTTCCCCTGCTCATATCCTCTCCTTCTCTTCT 300


HvEC_cDNA_032      ----------CACTCACTTTCTCATCTCCGCTTCCTTTGACGGTCACATCAAATTTTAGA 210
HvEC_gDNA_032      CTCTACACAGCACTCACTTTCTCATCTCCGCTTCCTTTGACGGTCACATCAAATTTTAGA 360
                             **************************************************

HvEC_cDNA_032      AAAAGAACGAACCTCATTCTTCCCCCGGCATCGAATTAG 249
HvEC_gDNA_032      AAAAGAACGAACCTCATTCTTCCCCCGGCATCGAATTAG 399
                   ***************************************
```

HvEC-033 alignment

```
CLUSTAL 2.1 multiple sequence alignment


HvEC_cDNA_033      ATGAGATATTTTAGTTGTTGGCTGTTATTGGCCCCCTTTTCTTTCTCTATTAAAAATGCG 60
HvEC_gDNA_033      ATGAGATATTTTAGTTGTTGGCTGTTATTGGCCCCCTTTTCTTTCTCTATTAAAAATGCG 60
                   ************************************************************

HvEC_cDNA_033      TTGGCTGGTTCTTCTCCGGTGGAATATGACACGGAGCAACAGCTACAATCCACTGCACAG 120
HvEC_gDNA_033      TTGGCTGGTTCTTCTCCGGTGGAATATGACACGGAGCAACAGCTACAATCCACTGCACAG 120
                   ************************************************************

HvEC_cDNA_033      TTAACGTTGAATGATTGGAACTCTGTTCAATCTGCTTTCACAATTGAGACTCCTCCTCAA 180
HvEC_gDNA_033      TTAACGTTGAATGATTGGAACTCTGTTCAATCTGCTTTCACAATTGAGACTCCTCCTCAA 180
                   ************************************************************

HvEC_cDNA_033      ACTTCACCTTCGCCTGAAAACCCAAAAATCAAAAATAACTCTCGACCTGTATCTATTATA 240
HvEC_gDNA_033      ACTTCACCTTCGCCTGAAAACCCAAAAATCAAAAATAACTCTCGACCTGTATCTATTATA 240
                   ************************************************************

HvEC_cDNA_033      AGTCTCACTCCTTCCTTCCTCAAGTGGTTAA 270
HvEC_gDNA_033      AGTCTCACTCCTTCCTTCCTCAAGTGGTTAA 270
                   *******************************
```

HvEC-034 alignment

```
CLUSTAL 2.1 multiple sequence alignment


HvEC_cDNA_034      ATGAAATTATTCTATTACACTCTTTTATCGCTCGTGTTGGCCTTCTCCTTAATTTCAAAC 60
HvEC_gDNA_034      ATGAAATTATTCTATTACACTCTTTTATCGCTCGTGTTGGCCTTCTCCTTAATTTCAAAC 60
                   ************************************************************

HvEC_cDNA_034      ACGAAGGCTGATACCGTAGTTTCATTGCTTCCTAAATTCAAAACTTGTGCCCAAGAAGCA 120
HvEC_gDNA_034      ACGAAGGCTGATACCGTAGTTTCATTGCTTCCTAAATTCAAAACTTGTGCCCAAGAAGCA 120
                   ************************************************************

HvEC_cDNA_034      TTTTTCAGAGCCTATGTTGATGCGGGAGCAAATTGTGGT--------------------- 159
HvEC_gDNA_034      TTTTTCAGAGCCTATGTTGATGCGGGAGCAAATTGTGGTGTAAGTAAATGTCTAACGAAT 180
                   ***************************************

HvEC_cDNA_034      ---------------------------------------ATTGATTTGAAGTGTCTTTGT 180
HvEC_gDNA_034      AATTTATGAGCGGATTCTGACGCAAATCTTTCGCTACAGATTGATTTGAAGTGTCTTTGT 240
                                                          *********************

HvEC_cDNA_034      CAAAGCCAGGCTTTCATTTGCGCAAGTGCGAACCGAATCACCTCCAAATGTTCATCATTT 240
HvEC_gDNA_034      CAAAGCCAGGCTTTCATTTGCGCAAGTGCGAACCGAATCACCTCCAAATGTTCATCATTT 300
                   ************************************************************

HvEC_cDNA_034      TTTGACCAACTTGGGGCATTGAGTACCTGTACTCAAG----------------------- 277
HvEC_gDNA_034      TTTGACCAACTTGGGGCATTGAGTACCTGTACTCAAGTAAGTCATCTTATCGTAAACTCT 360
                   *************************************

HvEC_cDNA_034      -------------------------------------------------GATGTCGATCT 288
HvEC_gDNA_034      AACTCTTCGATAATCACGGTGCGTTACTCATATATTACTTTTAATTAGGGATGTCGATCT 420
                                                                    ***********

HvEC_cDNA_034      GTTGGTGTTTCTAGCATCAATGTAAATTCGTGCTATTGA 327
HvEC_gDNA_034      GTTGGTGTTTCTAGCATCAATGTAAATTCGTGCTATTGA 459
                   ***************************************
```

HvEC-035 alignment

```
CLUSTAL 2.1 multiple sequence alignment


HvEC_cDNA_035      ATGTTTTTGCGGTCTATAATTTTTTTTACTCTGAGCT----------------------- 37
HvEC_gDNA_35A      ATGTTTTTGCGGTCTATAATTTTTTTTACTCTGAGCT----------------------- 37
HvEC_gDNA_35B      ATGTTTTTGCGGTCTATAATTTTTTTTACTCTGAGCTGTTAGTTGACCCCTCATATATCG 60
                   *************************************

HvEC_cDNA_035      ---------------------------------------TCATTGGCTTTTCCCATGCTG 58
HvEC_gDNA_35A      ---------------------------------------TCATTGGCTTTTCCCATGCTG 58
```

```
HvEC_gDNA_35B      TTTCGTGTTACTTCTGATACTCATTTTTATTTTATTTAGTCATTGGCTTTTCCCATGCTG 120
                                                          *********************

HvEC_cDNA_035      CTCTACAACAAGGACTTAAAAG-------------------------------------- 80
HvEC_gDNA_35A      CTCTACAACAAGGACTTAAAAG-------------------------------------- 80
HvEC_gDNA_35B      CTCTACAACAAGGACTTAAAAGGTATGCAAATTCAATAGCGAAGAAAATATTCATCTTTC 180
                   **********************

HvEC_cDNA_035      ------------------------------CCTTGGTGATTTCTCGGATGCTTCTACCAA 110
HvEC_gDNA_35A      ------------------------------CCTTGGTGATTTCTCGGATGCTTCTACCAA 110
HvEC_gDNA_35B      TAATTAACTGTGTATTTTCAAAATTTGAAGCCTTGGTGATTTCTCGGATGCTTCTACCAA 240
                                                 ******************************

HvEC_cDNA_035      TTTGGGGGCCTATTCCAATCCTTTAGACCTAATTCATTTGGAGCGAAGAACTTATAGTCC 170
HvEC_gDNA_35A      TTTGGGGGCCTATTCCAATCCTTTAGACCTAATTCATTTGGAGCGAAGAACTTATAGTCC 170
HvEC_gDNA_35B      TTTGGGGGCCTATTCCAATCCTTTAGACCTAATTCATTTGGAGCGAAGAACTTATAGTCC 300
                   ************************************************************

HvEC_cDNA_035      AAGGTCAAATGG------------------------------------------------ 182
HvEC_gDNA_35A      AAGGTCAAATGG------------------------------------------------ 182
HvEC_gDNA_35B      AAGGTCAAATGGGTGAAGCCTCTAACTCTTTCTTCTCGTTGCACTCTAAGTTATGCTAAA 360
                   ************

HvEC_cDNA_035      ------------------------------------------------------------
HvEC_gDNA_35A      ------------------------------------------------------------
HvEC_gDNA_35B      CGGAAACATCTTCTACACGGCTTTACATTATATATATATATTGCCTGTGGTATGATTTTT 420


HvEC_cDNA_035      ------------ATTGAAGCCCCATAAAAGACCTCTGAAAGAGTCAAGAGCTTTGAGCTT 230
HvEC_gDNA_35A      ------------ATTGAAGCCCCATAAAAGACCTCTGAAAGAGTCAAGAGCTTTGAGCTT 230
HvEC_gDNA_35B      TCCTTTTCCCAGATTGAAGCCCCATAAAAGACTTCTGAAAGAGTCAAGAGCTTTGGGCTT 480
                               ******************** **********************.****

HvEC_cDNA_035      ATCCATACCGAGTCAAACTTTATATGAACTGACCCAGAACCAAAAGGATAACAGTGGTCC 290
HvEC_gDNA_35A      ATCCATACCGAGTCAAACTTTATATGAACTGACCCAGAACCAAGAGGATAACAGTGGTCC 290
HvEC_gDNA_35B      ATCCATACCGAGTCAAACTTTATATGAACTGTCCCAGAACCAAGAGGATAACAGTGGTCC 540
                   *******************************:***********.****************

HvEC_cDNA_035      TTTCACTATAATTGATAATCTTTCATCAAAACTCGACTCTAGTTCCTTTATAGAGATAGG 350
HvEC_gDNA_35A      TTTCACTATAATTGATAATCTTTCATCAAAACTCGACTCTAGTTCCTTTATAGAGATAGG 350
HvEC_gDNA_35B      TTTCACTATAATTGATAATCTTTCATCAAAACTCGACTCTAGTTCCTTTGTAAAGATTGG 600
                   *************************************************.**.****.**

HvEC_cDNA_035      GGGTCAAGTTTTAAGCAAATCTTGA 375
HvEC_gDNA_35A      GGGTCAAGTTTTAAGCAAATCTTGA 375
HvEC_gDNA_35B      GGGTCAAGTTTTAAGCAAATCTTGA 625
                   *************************
```

HvEC-036 alignment

```
CLUSTAL 2.1 multiple sequence alignment


HvEC_cDNA_036      ATGAGATTTTCATTATATTCTATATCTTATTCTGCTATACTGCTGCTATCATTCCTTTTA 60
HvEC_gDNA_036      ATGAGATTTTCATTATATTCTATATCTTATTCTGCTATACTGCTGCTATCATTCCTTTTA 60
                   ************************************************************

HvEC_cDNA_036      AACGTCCAAATACAAAGTGTATTGGCTTTAAGTTCAAGCAATCAACTGGAGAATCTACTT 120
HvEC_gDNA_036      AACGTCCAAATACAAAGTGTATTGGCTTTAAGTTCAAGCAATCAACTGGAGAATCTACTT 120
                   ************************************************************

HvEC_cDNA_036      CTACAACAAGGAAACAACCAGGATAATCATCATCATTATGAGATCAGTAAAACAAAGCGG 180
HvEC_gDNA_036      CTACAACAAGGAAACAACCAGGATAATCATCATCATTATGAGATCAGTAAAACAAAGCGG 180
                   ************************************************************

HvEC_cDNA_036      AGTAAATTTTTGGATCGCATAAAGAGGAATCAAGTAAGCATTGAACAACTCATTAAGCAA 240
HvEC_gDNA_036      AGTAAATTTTTGGATCGCATAAAGAGGAATCAAGTAAGCATTGAACAACTCATTAAGCAA 240
                   ************************************************************

HvEC_cDNA_036      AACCTAAGGAGACAACTCCGTAAAAGAAATGACGGCGAAGCTTCAGTTCCTTATCATACT 300
HvEC_gDNA_036      AACCTAAGGAGACAACTCCGTAAAAGAAATGACGGCGAAGCTTCAGTTCCTTATCATACT 300
                   ************************************************************

HvEC_cDNA_036      GGATCACAAACTTAA 315
HvEC_gDNA_036      GGATCACAAACTTAA 315
                   ***************
```

HvEC-037 alignment

```
CLUSTAL 2.1 multiple sequence alignment


HvEC_cDNA_037      ATGAAGTTGCTCTTATCGTTTGTTTCTGCCACACTTTGCATTTTGCAAGCAACTAGTCAA 60
HvEC_gDNA_037      ATGAAGTTGCTCTTATCGTTTGTTTCTGCCACACTTTGCATTTTGCAAGCAACTAGTCAA 60
                   ************************************************************

HvEC_cDNA_037      AGAATTAGAATTGTTTCACCCACATCTGGACAAACCATTACCCTAGGATCAACGCTAAAT 120
HvEC_gDNA_037      AGAATTAGAATTGTTTCACCCACATCTGGACAAACCATTACCCTAGGATCAACGCTAAAT 120
                   ************************************************************

HvEC_cDNA_037      GTTACAATCGAACAAGAAGGT--------------------------------------- 141
HvEC_gDNA_037      GTTACAATCGAACAAGAAGGTGCGACTGTTCGTTTCTTTAATGTAAATCCACGTCCAGTA 180
                   *********************

HvEC_cDNA_037      ------------------------------------AGTTCGACGAACGTTGAGCCAATC 0165
HvEC_gDNA_037      ATATTTACATGGCAAAATATTTTTTTAATTTCAGGTAGTTCGACGAACGTTGAGCCAATC 240
                                                       ************************

HvEC_cDNA_037      GCTTTGACTGCGGGATTGAGTAACTTTTTTGATAAAGCGCCACAGTCTTTGGGAGGAAGA 225
HvEC_gDNA_037      GCTTTGACTGCGGGATTGAGTAACTTTTTTGATAAAGCGCCACAGTCTTTGGGAGGAAGA 298
                   ************************************************************

HvEC_cDNA_037      AATCCTAACTTGGGCCAGTTTGCCAAAAAGATCAGTTGA 264
HvEC_gDNA_037      AATCCTAACTTGGGCCAGTTTGCCAAAAAGATCAGTTGA 332
                   ***************************************
```

HvEC-038 alignment

```
CLUSTAL 2.1 multiple sequence alignment


HvEC_cDNA_038      ATGAGGATGCATTTAATAGTCTCACTTGCATTGGTACTGCGTGTTTATTCGGAGGATGGT 60
HvEC_gDNA_038      ATGAGGATGCATTTAATAGTCTCACTTGCATTGGTACTGCGTGTTTATTCGGAGGATGGT 60
                   ************************************************************

HvEC_cDNA_038      GTGACAATATTCCCTTCTTCCGATTGCACTGGCCATAATTTTGACAGACATCCAATAATT 120
HvEC_gDNA_038      GTGACAATATTCCCTTCTTCCGATTGCACTGGCCATAATTTTGACAGACATCCAATAATT 120
                   ************************************************************

HvEC_cDNA_038      GGAGTGGAGATGCCATACGATTCTGGCTGCATTCCAGTACCCCCATCAAGCCGCTCAGCT 180
HvEC_gDNA_038      GGAGTGGAGATGCCATACGATTCTGGCTGCATTCCAGTACCCCCATCAAGCCGCTCAGCT 180
                   ************************************************************

HvEC_cDNA_038      GATAGCCGCTTCACTGGAAAAAATGCGAAATCTTGTAGGTTGGCAGTATTCCGCGAAAGT 240
HvEC_gDNA_038      GATAGCCGCTTCACTGGAAAAAATGCGAAATCTTGTAGGTTGGCAGTATTCCGCGAAAGT 240
                   ************************************************************

HvEC_cDNA_038      GATTGTCTTACCCAGATCCAGTTATCTAATGTAACCGGTCATTTGGGCTGCGGCCGTTCT 300
HvEC_gDNA_038      GATTGTCTTACCCAGATCCAGTTATCTAATGTAACCGGTCATTTGGGCTGCGGCCGTTCT 300
                   ************************************************************

HvEC_cDNA_038      AACATACCAATCCAAGCATATGTGATAACATGTATATGA 339
HvEC_gDNA_038      AACATACCAATCCAAGCATATGTGATAACATGTATATGA 339
                   ***************************************
```

HvEC-039 alignment

```
CLUSTAL 2.1 multiple sequence alignment


HvEC_cDNA_039      ATGCGGTTTACCTTGTGTCAACAGTTTCCCGTTTTCATAAGTTTTCTACAAAGCCTCTTT 60
HvEC_gDNA_039      ATGCGGTTTACCTTGTGTCAACAGTTTCCCGTTTTCATAAGTTTTCTACAAAGCCTCTTT 60
                   ************************************************************

HvEC_cDNA_039      GCTTTACCTGTGCCCGAACTTGCTTATTCAGGGAATAAAATGTATGCTAGAAATGGAAAA 120
HvEC_gDNA_039      GCTTTACCTGTGCCCGAACTTGCTTATTCAGGGAATAAAATGTATGCTAGAAATGGAAAA 120
                   ************************************************************

HvEC_cDNA_039      ATTCTGGCTGCATTGAATCCAGGAACATCAAATTTGACGAAAAACCATACCACAGATGAC 180
HvEC_gDNA_039      ATTCTGGCTGCATTGAATCCAGGAACATCAAATTTGACGAAAAACCATACCACAGATGAC 180
                   ************************************************************
```

```
HvEC_cDNA_039      AGAGAAGCAGAAACGCCAACAGT------------------------------------- 203
HvEC_gDNA_039      AGAGAAGCAGAAACGCCAACAGTGTAAGCGTAATCACTACTCGGGATGACATATTTTCGC 240
                   ***********************

HvEC_cDNA_039      -------------------------------------------TGTAGATCACTTTGTTG 220
HvEC_gDNA_039      GGTGCAAAACGAACTAAAAAAATTCATTTCTTTCTATACTTAGTGTAGATCACTTTGTTG 300
                                                              *****************

HvEC_cDNA_039      ATGCTTGGGGCAAAGTGAATGGGGAGTGTACCCACGAAAAAATATTTTGA 270
HvEC_gDNA_039      ATGCTTGGGGCAAAGTGAATGGGGAGTGTACCCACGAAAAAATATTTTGA 350
                   **************************************************
```

HvEC-040 alignment

```
CLUSTAL 2.1 multiple sequence alignment


HvEC_cDNA_040      ATGAATTTTGATCTTCTTCTTCTTCTTCTTCTCGGCTTTTTGATCTCCGTCTGTTTTCAT 60
HvEC_gDNA_040      ATGAATTTTGATCTTCTTCTTCTTCTTCTTCTCGGCTTTTTGATCTCCGTCTGTTTTCAT 60
                   ************************************************************

HvEC_cDNA_040      CGCTCAGCCAACGCCTTCCAATTCCCTTTTTTATACCACAACAATATCCATAACACTTTC 120
HvEC_gDNA_040      CGCTCAGCCAACGCCTTCCAATTCCCTTTTTTATACCACAACAATATCCATAACACTTTC 120
                   ************************************************************

HvEC_cDNA_040      CAGCTAACTCATGCCATCCGTGCTCGTTTCACCAATGGGAAGCTCAAAGAAGACACAACA 180
HvEC_gDNA_040      CAGCTAACTCATGCCATCCGTGCTCGTTTCACCAATGGGAAGCTCAAAGAAGACACAACA 180
                   ************************************************************

HvEC_cDNA_040      TCCTTCTCAGTCTTGCCGCTAGAGGTTCAAAAAACGCTTCGGACACGTTGCTCATACAGG 240
HvEC_gDNA_040      TCCTTCTCAGTCTTGCCGCTAGAGGTTCAAAAAACGCTTCGGACACGTTGCTCATACAGG 240
                   ************************************************************

HvEC_cDNA_040      ATTCATTCCAGCCACTCAAATGGAAATCTTTTCCTATCCGTGGCCCTGATGTCCAGCATC 300
HvEC_gDNA_040      ATTCATTCCAGCCACTCAAATGGAAATCTTTTCCTATCCGTGGCCCTGATGTCCAGCATC 300
                   ************************************************************

HvEC_cDNA_040      TCAACACTCTTTCAACACTCGCTAAGATGA 330
HvEC_gDNA_040      TCAACACTCTTTCAACACTCGCTAAGATGA 330
                   ******************************
```

HvEC-041 alignment

```
CLUSTAL 2.1 multiple sequence alignment


HvEC_cDNA_041      ATGCAGTTGCTTCCTTTGGTCTTATTTGTATTTCTCTTGATTGGCGGTTCTAAGGAAGGA 60
HvEC_gDNA_041      ATGCAGTTGCTTCCTTTGGTCTTATTTGTATTTCTCTTGATTGGCGGTTCTAAGGAAGGA 59
                   ************************************************************

HvEC_cDNA_041      TATGCGACAGTTTACAGTGACGGGAAATGCTCAGGAAATGATTTAGGAAACACGTCAATC 120
HvEC_gDNA_041      TATGCGACAGTTTACAGTGACGGGAAATGCTCAGGAAATGATTTAGGAAACACGTCAATC 119
                   ************************************************************

HvEC_cDNA_041      ACAGCAAATACACAAGTTTATACTGAATGTATCCCGACATCCGGTCCAGGAAAATCGATA 180
HvEC_gDNA_041      ACAGCAAATACACAAGTTTATACTGAATGTATCCCGACATCCGGTCCAGGAAAATCGATA 179
                   ************************************************************

HvEC_cDNA_041      AGGTTATATTTGGAGGATATTGATATTAGTTGGTGCGCAGTGAAGCCATACAACGACACT 240
HvEC_gDNA_041      AGGTTATATTTGGAGGATATTGATATTAGTTGGTGCGCAGTGAAGCCATACAACGACACT 239
                   ************************************************************

HvEC_cDNA_041      AACTGCATCAAAGATTACCCAGCCCTCAGAGGTATTGTACCTTTTGGATGTCAAGATAAT 300
HvEC_gDNA_041      AACTGCATCAAAGATTACCCAGCCCTCAGAGGTATTGTACCTTTTGGATGTCAAGATAAT 299
                   ************************************************************

HvEC_cDNA_041      GATAGGATTATGGGATTTCGAGTTTCGTGTAGGAATTGA 339
HvEC_gDNA_041      GATAGGATTATGGGATTTCGAGTTTCGTGTAGGAATTGA 338
                   ***************************************
```

HvEC-043 alignment

```
CLUSTAL 2.1 multiple sequence alignment


HvEC_cDNA_043      ATGAACTTCATCGTACTTTGTGCCTTAATCTTTAATACTGCAGTATTTACCCAGGCTGCT 60
HvEC_gDNA_043      ATGAACTTCATCGTACTTTGTGCCTTAATCTTTAATACTGCAGTATTTACCCAGGCTGCT 60
                   ************************************************************

HvEC_cDNA_043      CCAGGAGCATTTGATCCATCATCCTTGCAAAGAAGAGGCATACTTACTTTTGGCGACGGA 120
HvEC_gDNA_043      CCAGGAGCATTTGATCCATCATCCTTGCAAAGAAGAGGCATACTTACTTTTGGCGACGGA 120
                   ************************************************************

HvEC_cDNA_043      AATAAAGTTTCCATGGCCAGTACAATTAGCCGTCGAAGTGATGATGGCGAGAATCCAGAT 180
HvEC_gDNA_043      AATAAAGTTTCCATGGCCAGTACAATTAGCCGTCGAAGTGATGATGGCGAGAATCCAGAT 180
                   ************************************************************

HvEC_cDNA_043      CCTATGAGTCAAAAGCTCGGCCGTCGATTTGGTTAA 2016
HvEC_gDNA_043      CCTATGAGTCAAAAGCTCGGCCGTCGATTTGGTTAA 2016
                   ************************************
```

HvEC-045 alignment

```
CLUSTAL 2.1 multiple sequence alignment


HvEC_cDNA_045      ATGTTACAGGTTACTGCGTTTAAACTTGTGCTCCTAGTCGCTTGTGTCTTAATAGATAAA 60
HvEC_gDNA_045      ATGTTACAGGTTACTGCGTTTAAACTTGTGCTCCTAGTCGCTTGTGTCTTAATAGATAAA 60
                   ************************************************************

HvEC_cDNA_045      ACGATCTCAACCACTTTTGGACGTTCTCGAGGTATTGGAGAGATGGGGATCAATTGTGGA 120
HvEC_gDNA_045      ACGATCTCAACCACTTTTGGACGTTCTCGAGGTATTGGAGAGATGGGGATCAATTGTGGA 120
                   ************************************************************

HvEC_cDNA_045      ATCATTATGCCCGGGTCGGAAAATAAGAAGCCTAGCTCCTCAGAGGATTGCATCCG---- 176
HvEC_gDNA_045      ATCATTATGCCCGGGTCGGAAAATAAGAAGCCTAGCTCCTCAGAGGATTGCATCCGGTAA 180
                   ********************************************************

HvEC_cDNA_045      ------------------------------------------------------------
HvEC_gDNA_045      AATATAATTTACATTGTCTACCGACTCTCTCTCCTAGAAAAACTTACCACTGCGTTAACG 240


HvEC_cDNA_045      --------TGCTCTAGAATTATATAGGGATTCCGAGAATCCCCAGTTTTTATTGCCTACT 228
HvEC_gDNA_045      ATGCAAAGTGCTCTAGAATTATATAGGGATTCCGAGAATCCCCAGTTTTTATTGCCTACT 300
                           ****************************************************

HvEC_cDNA_045      GGCTTGTATGAATTTCGCTCGTTTGGAAGTTGTGAAGT---------------------- 266
HvEC_gDNA_045      GGCTTGTATGAATTTCGCTCGTTTGGAAGTTGTGAAGTAAATTTTAATTAGACTTAAAGT 360
                   **************************************

HvEC_cDNA_045      --------------------------------------------------TCGTTTGTTA 276
HvEC_gDNA_045      GCTTTACAACTTCATTTACTCCTGTCTCAATATTATTGAATGATGTAGGTTCGTTTGTTA 420
                                                                     **********

HvEC_cDNA_045      CATGAAAGAGGAAAAGAGGAGGT------------------------------------- 299
HvEC_gDNA_045      CATGAAAGAGGAAAAGAGGAGGTGTGAGTGTCTCTTCAACAGGCTGCCATAATAATTCAG 480
                   ***********************

HvEC_cDNA_045      -----------------------------------CGATTATCAATCAGTTATAAAAAAA 324
HvEC_gDNA_045      TAAAAGCCTCTTACATTTCATGTATGTTTTTGCAGCGATTATCAATCAGTTATAAAAAAA 540
                                                      *************************

HvEC_cDNA_045      GTGAAAACTATTACTGAAGCATGTCAGGTGGCT--------------------------- 357
HvEC_gDNA_045      GTGAAAACTATTACTGAAGCATGTCAGGTGGCTGTAAGCTTGCATATTAGCTCCCATCTT 600
                   *********************************

HvEC_cDNA_045      -------------------------------------AACGGTCAAGGAGACGTGGACAG 380
HvEC_gDNA_045      TGCAGGTGTATACAAATTATCATTTTTGTTATTTCAGAACGGTCAAGGAGACGTGGACAG 659
                                                        ***********************

HvEC_cDNA_045      ATGA 384
HvEC_gDNA_045      ATGA 663
                   ****
```

HvEC-046 alignment

```
CLUSTAL 2.1 multiple sequence alignment


HvEC_cDNA_046      ATGCATTTTTCACTTGCTTCCCTCATCGTTCTTAGTG----------------------- 37
HvEC_gDNA_046      ATGCATTTTTCACTTGCTTCCCTCATCGTTCTTAGTGGTTAGTTATGTTGATTCTATGAA 60
                   *************************************

HvEC_cDNA_046      ----------------------------------------------------CCCACATT 45
HvEC_gDNA_046      AAGATGATTTAAACTAATTAATCATATATATCTCTACATTTGATTGTAAAAGCCCACATT 120
                                                                       ********

HvEC_cDNA_046      TTGAACATCTCCGCACTTCCTTATGTTGAGGAAATAAATGCTTCCAA------------- 92
HvEC_gDNA_046      TTGAACATCTCCGCACTTCCTTATGTTGAGGAAATAAATGCTTCCAAGTCAGTTGATCAG 180
                   ***********************************************

HvEC_cDNA_046      ---------------------------------------------------------TTA 95
HvEC_gDNA_046      GTATTAATCCCTTACAGAGATTTGAACTGAAATTTTTTATCTTTTGTATAAATCTAGTTA 240
                                                                            ***

HvEC_cDNA_046      CCTTCAACCACGAACCCTCAGAGATACTTATTCGTCCCCTAAACCTAGTTACTCGTCACC 155
HvEC_gDNA_046      CCTTCAACCACGAACCCTCAGAGATACTTATTCGTCCCCTAAACCTAGTTACTCGTCACC 300
                   ************************************************************

HvEC_cDNA_046      TGGCGCTGGTTATTCGTCACCTAGCGCTGGTTATTCGTCTCCTAATCCTGGTTACGTGTC 215
HvEC_gDNA_046      TGGCGCTGGTTATTCGTCACCTAGCGCTGGTTATTCGTCTCCTAATCCTGGTTACGTGTC 360
                   ************************************************************

HvEC_cDNA_046      TCCTACGCCTGGCTATGTGTCTCCATCTCCCTCCAAGCCCGATTCCTATTCAACAAATCC 275
HvEC_gDNA_046      TCCTACGCCTGGCTATGTGTCTCCATCTCCCTCCAAGCCCGATTCCTATTCAACAAATCC 420
                   ************************************************************

HvEC_cDNA_046      TTACTCTGTAGATAATGTCTTCAATCGCGGAGTAGGTTTGATACCTGGTTTCGGTACAGG 335
HvEC_gDNA_046      TTACTCTGTAGATAATGTCTTCAATCGCGGAGTAGGTTTGATACCTGGTTTCGGTACAGG 480
                   ************************************************************

HvEC_cDNA_046      TGTAGGAGCTACTGTGAAAACTGGTGTAACATACGATCTCATGATTAAAAGTAGTGCGCA 395
HvEC_gDNA_046      TGTAGGAGCTACTGTGAAAACTGGTGTAACATACGATCTCATGATTAAAAGTAGTGCGCA 540
                   ************************************************************

HvEC_cDNA_046      AATCAGTGTTGGCTTACAAAGTACAGCCGAAATCGGATCAGCTATTGGAGCTCAAGTGAT 455
HvEC_gDNA_046      AATCAGTGTTGGCTTACAAAGTACAGCCGAAATCGGATCAGCTATTGGAGCTCAAGTGAT 600
                   ************************************************************

HvEC_cDNA_046      CTCTCAATTTTATCTCTTATTGGGGCTAAATGCTCAATCTGCTGCGACTCTTGATGTGCA 515
HvEC_gDNA_046      CTCTCAATTTTATCTCTTATTGGGGCTAAATGCTCAATCTGCTGCGACTCTTGATGTGCA 660
                   ************************************************************

HvEC_cDNA_046      AGGTTTAAGTCAATTAGCTGTTAAATCTGGTTTATCAGTTTAA 558
HvEC_gDNA_046      AGGTTTAAGTCAATTAGCTGTTAAATCTGGTTTATCAGTTTAA 703
                   *******************************************
```

HvEC-047 alignment

```
CLUSTAL 2.1 multiple sequence alignment


HvEC_cDNA_047      ATGTTGGCTTCGGACTTTATCGCTGTCACATTATTAG----------------------- 37
HvEC_gDNA_047      ATGTTGGCTTCGGACTTTATCGCTGTCACATTATTAGGTTAGTACTCCAATATTACTTCA 60
                   *************************************

HvEC_cDNA_047      ---------------------------------CTTTCTTTGGCTTTGCTCTATCATC-- 62
HvEC_gDNA_047      ACATTGAGTGTAAACTGAAAATTTTCCCTACAGCTTTCTTTGGCTTTGCTCTATCATCGT 120
                                                    *************************

HvEC_cDNA_047      ---------------------------------------------------TCCCGAACC 71
HvEC_gDNA_047      AAGCTGTTAAAGTACTTCCATAAACAGGGGAATCGCTCATTTTTCTTGTAGTCCCGAACC 180
                                                                      *********

HvEC_cDNA_047      ATGCCCAACCTGCCTACCACCCGTTCAATTCCCACCTAACCCATGCGGCCAATCAGCTTG 131
HvEC_gDNA_047      ATGCCCAACCTGCCTACCACCCGTTCAATTCCCACCTAACCCATGCGGCCAATCAGCTTG 240
                   ************************************************************

HvEC_cDNA_047      CCCAGACAAATGTGATGCCTGTTCCCAACCCCCCCCACCAGCCTGTAAAGTTTGCTCTGG 191
HvEC_gDNA_047      CCCAGACAAATGTGATGCCTGTTCCCAACCCCCCCCACCAGCCTGTAAAGTTTGCTCTGG 300
                   ************************************************************
```

```
HvEC_cDNA_047      TCTTGGCCCTGACCAAGGTGGCTACCCGTCATACGGAGGGGGACAAGGCGGTTATGGTGG 251
HvEC_gDNA_047      TCTTGGCCCTGACCAAGGTGGCTACCCGTCATACGGAGGGGGACAAGGCGGTTATGGTGG 360
                   ************************************************************

HvEC_cDNA_047      TCAACCCGGTATCGGCGGTGGCTTTGGTCCACCCGGTCTCGACGGCTTTGGCGGTCAAAC 311
HvEC_gDNA_047      TCAACCCGGTATCGGCGGTGGCTTTGGTCCACCCGGTCTCGACGGCTTTGGCGGTCAAAC 419
                   ************************************************************

HvEC_cDNA_047      CCGGTGGTGGTCCTCCTCCAGGCCTTTTGGACGGACTTTTGGGTCGTGGTTTGCTACAAG 371
HvEC_gDNA_047      CCGGTGGTGGTCCTCCTCCAGGCCTTTTGGACGGACTTTTGGGTCGTGGTTTGCTACAAG 478
                   ************************************************************

HvEC_cDNA_047      AGGTCCAAGGCCGAGTCTAATTAATGTTGTGTTTCCCTATCTTGCGTCCTCCAGATTTTC 431
HvEC_gDNA_047      AGGTCCAAGGCCGAGTCTAATTAATGTTGTGTTTCCCTATCTTGCGTCCTCCAGATTTTC 538
                   ************************************************************

HvEC_cDNA_047      GTTGTATGGCTTTTTATGA 450
HvEC_gDNA_047      GTTGTATGGCTTTTTATGA 557
                   *******************
```

HvEC-054 alignment

```
CLUSTAL multiple sequence alignment by MUSCLE (3.8)

HvEC_cDNA_054      ATGGTATCCTACAGACCAGTATTATACTTATTGCTAGCCGTAGCTATGATTTCTGTATCA
HvEC_gDNA_054      ATGGTATCCTACAGACCAGTATTATACTTATTGCTAGCCGTAGCTATGATTTCTGTATCA
                   ************************************************************

HvEC_cDNA_054      TCGGCGCAAGAGCAATCTGGTACTAGTAATACCAGCACATCCAAAGCTCCTACA------
HvEC_gDNA_054      TCGGCGCAAGAGCAATCTGGTACTAGTAATACCAGCACATCCAAAGCTCCTACAGTTAGT
                   ******************************************************

HvEC_cDNA_054      ------------------------------------------------------------
HvEC_gDNA_054      ATTTGCATAGAGCTATTTGCGTTTCTTACCCAAACGTCGTTCTTAAGCTGATTCCGTTGC


HvEC_cDNA_054      --------GCCCCAGGTGCATCAGTAACCAGCTCCCTCGGCAACACCACTGCCAATACCA
HvEC_gDNA_054      AACTCTAGGCCCCAGGTGCATCAGTAACCAGCTCCCTCGGCAACACCACTGCCAATACCA
                           ****************************************************

HvEC_cDNA_054      CAGCTGGTTCCACCCTTAACACTACCGAATCAGTTTCTCCTACATCTGGTTCCAACATGA
HvEC_gDNA_054      CAGCTGGTTCCACCCTTAACACTACCGAATCAGTTTCTCCTACATCTGGTTCCAACATGA
                   ************************************************************

HvEC_cDNA_054      CAAACATCACTGGTACGGCTGGTGGGGCTAGTCTTGGAGACGCCAGTCATTCTGCCTCAG
HvEC_gDNA_054      CAAACATCACTGGTACGGCTGGTGGGGCTAGTCTTGGAGACGCCAGTCATTCTGCCTCAG
                   ************************************************************

HvEC_cDNA_054      GAGGTAATAAAACATCCAACACCAGCACCTCAAGTACCCGCCCCACTACCTCAACCAGTA
HvEC_gDNA_054      GAGGTAATAAAACATCCAACACCAGCACCTCAAGTACCCGCCCCACTACCTCAACCAGTA
                   ************************************************************

HvEC_cDNA_054      GCACCACCTCTCCAGCCTCAAACACCTCTACCACTACCGATTCA----------------
HvEC_gDNA_054      GCACCACCTCTCCAGCCTCAAACACCTCTACCACTACCGATTCAGTAAGTCTATTGCTGC
                   ********************************************

HvEC_cDNA_054      ------------------------------------------------------------
HvEC_gDNA_054      TAAAACTTTCTAATACTGTTAGATTACAGCTGTAATAACTCGATTGTTCTTGACCCTCCT


HvEC_cDNA_054      -------AAACAACCAGGCGCTGCATCTCACCGCGGACCACAAGTTGCTGCTGCTGC-AG
HvEC_gDNA_054      TCAACAGAAACAACCAGGCGCTGCATCTCACCGCGGACCACAAGTTGCTGCTGCTGCTAG
                          ************************************************** **

HvEC_cDNA_054      TTGTTGCTGGTCTATCTGTGCTTATTCTTTAA
HvEC_gDNA_054      TTGTTGCTGGTCTATCTGTGCTTATTCTTTAA
                   ********************************
```

HvEC-057 alignment

```
CLUSTAL multiple sequence alignment by MUSCLE (3.8)


HvEC_cDNA_057      ATGTCTTTACTAAGATTCTTTCTTTTCGTGAGCTTAGCATGTTTATGGTACAATGCGAAA
HvEC_gDNA_057      ATGTCTTTACTAAGATTCTTTCTTTTCGTGAGCTTAGCATGTTTATGGTACAATGCGAAA
                   ************************************************************

HvEC_cDNA_057      GCATATGATTTGGGCTGCGATTCCTTTAGGAAGGACAGCCAAACACAAC-----------
HvEC_gDNA_057      GCATATGATTTGGGCTGCGATTCCTTTAGGAAGGACAGCCAAACACAACGTAAGTAGAAG
                   *************************************************

HvEC_cDNA_057      ------------------------------------------------------------
HvEC_gDNA_057      CCTTTATGATTTCTGGCCGGAAATCTCCCCTTGATATCTTGTTTGTATCCCTTTTTATTT

HvEC_cDNA_057      ------------------------------------AGTACAATTGTTCAAATGGAGGTA
HvEC_gDNA_057      TTATTTTTATATTTTTATTTTTATTCGCTTTTTCAGAGTACAATTGTTCAAATGGAGGTA
                                                       ************************

HvEC_cDNA_057      AAACAGTCCAATGTGGATCCTGTCAATATCCCT---------------------------
HvEC_gDNA_057      AAACAGTCCAATGTGGATCCTGTCAATATCCCTGTGAGTTCTACAGTTAATCATTTCCGA
                   *********************************

HvEC_cDNA_057      --------------------------------------CGGCAAACCATTGCGTTTACCC
HvEC_gDNA_057      TTAGAACTACTACCAATAATATTTTGGATGATGATCAGCGGCAAACCATTGCGTTTACCC
                                                         **********************

HvEC_cDNA_057      CGGCAATACCAAACATAATATCAATAGCCTTAAGTGTGAATGGGGTTACTCCACAGCAAG
HvEC_gDNA_057      CGGCAATACCAAACATAATATCAATAGCCTTAAGTGTGAATGGGGTTACTCCACAGCAAG
                   ************************************************************

HvEC_cDNA_057      TGCAAACAGAGGCACGCCGGAGGCAT----------------------------------
HvEC_gDNA_057      TGCAAACAGAGGCACGCCGGAGGCATGTAAGATGAAAATGTTTTACTTACCAARACCGTC
                   **************************

HvEC_cDNA_057      ------------------------------------------TATGTCGCACAAGTAATG
HvEC_gDNA_057      AATTTTTTAAAATGTTTTAATAACCACCAATCACCATTTTAGTATGTCGCACAAGTAATG
                                                             ******************

HvEC_cDNA_057      ATAAAGTAGCTTACTGTACCGCCCCAACCAGTCCAATTGTCATTC---------------
HvEC_gDNA_057      ATAAAGTAGCTTACTGTACCGCCCCAACCAGTCCAATTGTCATTCGTAAGTGAATAACTG
                   *********************************************

HvEC_cDNA_057      ------------------------------------------------------------
HvEC_gDNA_057      TTTTTTTTCAGAAAAGAGTAAATTAATTCACCAATATTCACTTTTTTTGAATCCCATGTA

HvEC_cDNA_057      ---AATGTACTCGCTGCGACAACAATTTGCCCCCTCCCGCCAAATAG
HvEC_gDNA_057      CAGAATGTACTCGCTGCGACAACAATTTGCCCCCTCCCGCCAAATAG
                      ********************************************
```

HvEC-071 alignment

```
CLUSTAL multiple sequence alignment by MUSCLE (3.8)

HvEC_cDNA_071      ATGATTTATTTGTCTTTGCTCCCCTTACTATTGAGTTCAATTGCCAATCCCTCCTTCCAG
HvEC_gDNA_071      ATGATTTATTTGTCTTTGCTCCCCTTACTATTGAGTTCAATTGCCAATCCCTCCTTCCAG
                   ************************************************************

HvEC_cDNA_071      GGGCAATTGTTCTTCTACAATGTTTCCTCTTGTATAGGCGCGCCGAATACTGCAAACTAT
HvEC_gDNA_071      GGGCAATTGTTCTTCTACAATGTTTCCTCTTGTATAGGCGCGCCGAATACTGCAAACTAT
                   ************************************************************

HvEC_cDNA_071      AACGAATCTCCCCAGGGCACTTCTCGGTGCTTCACCATCGAAGGCAGTGCTCCCCACGCT
HvEC_gDNA_071      AACGAATCTCCCCAGGGCACTTCTCGGTGCTTCACCATCGAAGGCAGTGCTCCCCACGCT
                   ************************************************************

HvEC_cDNA_071      GTTGTCATGTTGCCTGATCGATTTCATCCCGGTAATTGCTTGTCTAGCTTTTTCCATAAT
HvEC_gDNA_071      GTTGTCATGTTGCCTGATCGATTTCATCCCGGTAATTGCTTGTCTAGCTTTTTCCATAAT
                   ************************************************************

HvEC_cDNA_071      AATAAATGTTCCGATCTATTAGGAGAAGTAAATGGGAGTACAGGCAGCTTCTGCCATCCC
HvEC_gDNA_071      AATAAATGTTCCGATCTATTAGGAGAAGTAAATGGGAGTACAGGCAGCTTCTGCCATCCC
```

```
                    ************************************************************

HvEC_cDNA_071       TTTAATGGCATTTGGTCTTACCGAATCCATTGTCTATAG
HvEC_gDNA_071       TTTAATGGCATTTGGTCTTACCGAATCCATTGTCTATAG
                    ***************************************
```

HvEC-087 alignment

```
CLUSTAL multiple sequence alignment by MUSCLE (3.8)

HvEC_cDNA_087       ATGTACCCATATTTTTT-AATGTGTTTATTTTTTTTAATTCTGACT-CAT-AAAGTTGAG
HvEC_gDNA_087       ATGTACCCATATTTTTTAAATGTGTTTATTTTTTTTCACTCTGACTCCATAAAAGTTGAG
                    ***************** ****************** * ******* *** *********

HvEC_cDNA_087       TCATACGACTCACAATGGATTAGTGTTTCGATCAAGCC--GATTACTGTTCAAAGCTTTT
HvEC_gDNA_087       TCATACGACTCACAATGGATTAGTGTTTCGATCAAGCCCGAATTACTGTTCAAAGCTTTT
                    **************************************   *******************

HvEC_cDNA_087       TGGGAAAATGTCTTCAGTTGGACGATGAGCAACAGAGATGCATACGCTTAGATCGAATAT
HvEC_gDNA_087       TGGGAAAATGTCTTCAGTTGGACGATGAGCAACAGAGATGCATACGCTTAGATCGAATAT
                    ************************************************************

HvEC_cDNA_087       GCCAATTTCCTAATCGTCAATTCACTATGACAACAATCAAGGATGGGAAGATCTTGGGCG
HvEC_gDNA_087       GCCAATTTCCTAATCGTCAATTCACTATGACAACAATCAAGGATGGGAAGATCTTGGGCG
                    ************************************************************

HvEC_cDNA_087       CAGATCTTAGCATGATGATAAATTTTCTCAATGCTACTGCTGCATGTTGGAATGCCATTC
HvEC_gDNA_087       CAGATCTTAGCATGATGATAAATTTTCTCAATGCTACTGCTGCATGTTGGAATGCCATTC
                    ************************************************************

HvEC_cDNA_087       AAACACAGGGGGCCATTCGCAAAACAGGTGGGAGAATAGACGCATCAGATGTGATTGTAA
HvEC_gDNA_087       AAACTCAGGGGGCCATTCGCAAAACAGGTGGGAGAGTAGATGCGTCAGATGTGATTTTAA
                    **** ****************************** **** ** ************ ***

HvEC_cDNA_087       AATGTAAAGCTATAAAAGATCCAAGCAAAGAAATGTTGAAACTTGTAACCTCACGAGTGA
HvEC_gDNA_087       AATGTAAAGCTATAAAAGATCCAAGCAAAGAAATGTTGAAACTTGTAACCTCACGAGTGA
                    ************************************************************

HvEC_cDNA_087       ACATAGCTTCTCGAGACAACTTGAAGGAAGCGACAGAGACGCTTACAACTTATCTCAATT
HvEC_gDNA_087       ACATAGCTTCTCGAGACAACTTGAAGGAAGCGACAGAGACGCTTACAACTTATCTCAATT
                    ************************************************************

HvEC_cDNA_087       TCCTTAT-----------------------------------------------------
HvEC_gDNA_087       TCCTTATGTTGGTGATTTCATATATTCTACAGTACTATTGTACAAGTAGTTGACATGTTT
                    *******

HvEC_cDNA_087       -----------AAAATTGTTTCCAATATTAGAAAATCAAGGCT-GGTTGGAGAGTACTCT
HvEC_gDNA_087       AGTATAAACAGAAAATTGTTTCCAATATTAGAAAATCAAGGCTGGGTTGGAGAGTACTCT
                               ******************************** ****************

HvEC_cDNA_087       ATGGCGTGGYGTAAATTATGCATTGGATATATGCTTCTCT--------------------
HvEC_gDNA_087       ATGGCCGGGTGTAAATTATGCATTGGATATATGCTTCTCTGTAAGTGATACCACCTTTTA
                    *****  ** ******************************

HvEC_cDNA_087       ------------------------------------------------------------
HvEC_gDNA_087       CACGTCATACGAGTTGTGATGAGAAACCCTGATGAACTACAAATGTCTCCGAATATTACC


HvEC_cDNA_087       -----------GTTGTAATGATTCCTAGCGAGCAAGCATTGCGATATTAA
HvEC_gDNA_087       CACATTTACAGGTTGTAATGATTCCTAGCGAGCAAGCATTGCGATATTAA
                               ***************************************
```

Notes S5 Coffee genotypes carrying S_H1 used to determine the effect of HvEC-016 on multiplication of *Pseudomonas syringae* pv. *garcae* 1202.

**Resistance genes**	**CIFC* code - coffee selection**	**Coffee physiological group**	**Species**	**Reference**
S_H1	128/2 - Dilla & Algue	α	*Coffea arabica*	Bettencourt & Noronha-Wagner (1971)
$S_H1,4$	134/4 - S12 Kaffa	I	*Coffea arabica*	Noronha-Wagner & Bettencourt (1967); Bettencourt & Noronha-Wagner (1971)
$S_H1,5$	87/1 - Geisha	C - (α × E)	*Coffea arabica*	Noronha-Wagner & Bettencourt (1967); Bettencourt & Noronha-Wagner (1971)
$S_H1,2,5$	1006/10 - KP 532	L - (C × D)	*Coffea arabica*	Noronha-Wagner & Bettencourt (1967); Bettencourt & Noronha-Wagner (1971)
$S_H1,3,5$	H153/2 - (87/1 Geisha X 33/1 S 228-23)	Z - (α × G)	*C. arabica* x *C. liberica*	Bettencourt (1981)
$S_H1,4,5$	635/3 - S12 Kaffa	W - (C × ɣ)	*Coffea arabica*	Bettencourt & Noronha-Wagner (1971)
$S_H1,2,4,5$	HW 17/12 - (134/4 S12 Kaffa X 35/2 S 286-7)	O - (I × D)	*Coffea arabica*	Bettencourt (1981)

*****Center for Research into Coffee Rusts, Portugal.

References

Altschul SF, Madden TL, Schäffer AA, Zhang J, Zhang Z, Miller W, Lipman, DJ. 1997. Gapped BLAST and PSI-BLAST: a new generation of protein database search programs. *Nucleic Acids Research* **25**:3389–3402.

Badel JL, Piquerez SJM, Greenshields D, Rallapalli G, Fabro G, Ishaque N, Jones JDG. 2013. In planta effector competition assays detect *Hyaloperonospora arabidopsidis* effectors that contribute to virulence and localize to different plant subcellular compartments. *Molecular Plant-Microbe Interactions* **26**:745–757.

Bettencourt AJ, Noronha-Wagner M. 1971. Genetic factors conditioning resistance of *Coffea arabica* L. to *Hemileia vastatrix* Berk. and Br. *Agronomia Lusitana* **31**:285–292.

Bettencourt AJ. 1981. *Melhoramento genético do cafeeiro: transferência de factores de resistência à Hemileia vastatrix Berk. and Br. para as principais cultivares de Coffea arabica L.* PhD thesis, Junta de Investigações científicas de ULTRAMAR/Centro de Ivestigação das Ferrugens do Cafeeiro, Oeiras, Portugal.

Bilgin DD, DeLucia EH, Clough SJ. 2009. A robust plant RNA isolation method suitable for Affymetrix GeneChip analysis and quantitative real-time RT-PCR. *Nature Protocols* **4**:333–340.

Carper-Lindley C, Dahlbeck D, Clark ET, Staskawicz BJ. 2002. Direct biochemical evidence for type III secretion-dependent translocation of the AvrBs2 effector into plant cells. *Proceedings of the National Academy of Sciences, USA* **11**:8336–8341.

Catanzariti AM, Dodds PN, Lawrence GJ, Ayliffe MA, Ellis JG. 2006. Haustorially-expressed secreted proteins from flax rust are highly enriched for avirulence elicitors. *Plant Cell* **18**:243–256.

Chevreux B, Pfisterer T, Drescher B, Driesel AJ, Muller WE, Wetter T, Suhai S. 2004. Using the miraEST assembler for reliable and automated mRNA transcript assembly and SNP detection in sequenced ESTs. *Genome Research* **14**:1147–1159.

Cristancho MA, Botero-Rozo DO, Giraldo W, Tabima J, Riaño-Pachón DM, Escobar C, Rozo Y, Rivera LF, Durán A, Restrepo S *et al.* 2014. Annotation of a hybrid partial genome of the coffee rust (*Hemileia vastatrix*) contributes to the gene repertoire catalog of the Pucciniales. *Frontier in Plant Science* **5**:594.

Emanuelsson O, Nielsen H, Brunak S, von Heijne G. 2000. Predicting subcellular localization of proteins based on their N-terminal amino acid sequence. *Journal of Molecular Biology* **300**:1005–1016.

Ewing B, Green P. 1998. Base-calling of automated sequencer traces using phred. II. Error probabilities. *Genome Research* **8**:186–194.

Fabro G, Steinbrenner J, Coates M, Ishaque N, Baxter L, Studholme DJ, Körner E, Allen RL, Piquerez SJM, Rougon-Cardoso A *et al.* 2011. Multiple candidate effectors from the oomycete pathogen *Hyaloperonospora arabidopsidis* suppress host plant immunity. *PLoS Pathogens* **7**:e1002348.

Fernandez D, Tisserant E, Talhinha P, Azinheira H, Vieira A, Petitot AS, Loureiro A, Poulain J. 2012. 454-pyrosequencing of *Coffea arabica* leaves infected by the rust fungus *Hemileia vastatrix* reveals *in planta*-expressed pathogen-secreted proteins and plant functions in a late compatible plant-rust interaction. *Molecular Plant Pathology* **13**:17–37.

Finn RD, Mistry J, Tate J, Coggill P, Heger A, Pollington JE, Gavin OL, Gunasekaran P, Ceric G, Forslund K. 2010. The Pfam protein families database. *Nucleic Acids Research* **38**:D211–222.

Gietz R, Woods R. 2002. Transformation of yeast by lithium acetate/single-stranded carrier DNA/polyethylene glycol method. *Methods in Enzymology* **350**:87–96.

Horton P, Park KJ, Obayashi T, Fujita N, Harada H, Adams-Collier CJ, Nakai K. 2007. WoLF PSORT: protein localization predictor. *Nucleic Acids Research* **35**:W585–587.

Huang X, Madan A. 1999. CAP 3: A DNA sequence assembly program. *Genome Research* **9**:868–877.

Kemen E, Kemen AC, Rafiqi M, Hempel U, Mendgen K, Hanh M, Voegele R. 2005. Identification of a protein from rust fungi transferred from haustoria into infected plant cells. *Molecular Plant-Microbe Interactions* **18**:1130–1139.

Krogh A, Larsson B, von Heijne G, Sonnhammer EL. 2001. Predicting transmembrane protein topology with a hidden Markov model: application to complete genomes. *Journal of Molecular Biology* **305**:567–580.

Lee SJ, Kelley BS, Damasceno CMB, Jonh BS, Kim BS, Kim BD, Rose JKC. 2006. A functional screen to characterize the secretomes of eukaryotic pathogens and their host *in planta*. *Molecular Plant-Microbe Interactions* **19**:1368–1377.

Livak KJ, Schmittgen TD. 2001. Analysis of relative gene expression data using real-time quantitative PCR and the $2^{-\Delta\Delta CT}$ method. *Methods* **25**:402–408.

Maia TA, Maciel-Zambolim E, Caixeta ET, Mizubuti ESG, Zambolim L. 2013. The population structure of *Hemileia vastatrix* in Brazil inferred from AFLP. *Australasian Plant Pathology* **42**:533–542.

Noronha-Wagner M, Bettencourt AJ. 1967. Genetic study of the resistance of *Coffea* spp. to leaf rust. Identification and behavior of four factors conditioning disease reaction in *Coffea arabica* to twelve physiologic races of *Hemileia vastatrix. Canadian Journal of Botany* **45**:2021–2031.

Petersen TN, Brunak S, von Heijne G, Nielsen H. 2011. SignalP 4.0: discriminating signal peptides from transmembrane regions. *Nature Methods* **8**:785–786.

Ramiro DA, Escoube J, Petitot AS, Nicole M, Maluf MP, Fernandez D. 2009. Biphasic haustorial differentiation of coffee rust (*Hemileia vastatrix* race II) associated with defence responses in resistant and susceptible coffee cultivars. *Plant Pathology* **58**:944–955.

Talhinhas P, Azinheira HG, Vieira B, Loureiro A, Tavares S, Batista D, Morin E, Petitot AS, Paulo OS, Poulain J *et al.* 2014. Overview of the functional virulent genome of the coffee leaf rust pathogen *Hemileia vastatrix* with an emphasis on early stages of infection. *Frontier in Plant Science* **5**:1–17.

Thompson JD, Higgins DG, Gibson TJ. 1994. CLUSTAL W: Improving the sensibility of progressive multiple sequence alignment through sequence weighting, position-specific gap penalties and weight matrix choice. *Nucleic Acids Research* **22**:4673–4680.

Upadhyaya NM, Mago R, Staskawicz BJ, Ayliffe MA, Ellis JG, Dodds PN. 2014. A bacterial type III secretion assay for delivery of fungal effector proteins into wheat. *Molecular Plant-Microbe Interactions* **27**:255–264.

Vieira A, Talhinhas P, Loureiro A, Duplessis S, Fernandez D, Silva MC, Paulo OS, Azinheira HG. 2011. Validation of RT-qPCR reference genes for *in planta* expression studies in *Hemileia vastatrix*, the causal agent of coffee leaf rust. *Fungal Biology* **115**:891–901.
